# Design, Synthesis, and Characterization of Dichlorobiphenyl-Derived Inhibitors of the Proprotein Convertase Furin

**DOI:** 10.1021/acs.jmedchem.5c02157

**Published:** 2025-11-30

**Authors:** Roman W. Lange, Charlotte Boller, Michael Loresch, Konstantin Bloch, Eva Böttcher-Friebertshäuser, Hans Brandstetter, Sven O. Dahms, Torsten Steinmetzer

**Affiliations:** † Institute of Pharmaceutical Chemistry, 9377 Philipps University, Marbacher Weg 10, D-35032 Marburg, Germany; ‡ Institute of Virology, Philipps University, Hans-Meerwein-Str. 2, 35043 Marburg, Germany; § Department of Biosciences and Medical Biology, Structural Biology Lab, Paris Lodron University, Hellbrunner Straße 34, A-5020 Salzburg, Austria; ∥ Center for Tumor Biology and Immunology (CTBI), 27257 University of Salzburg, Hellbrunner Straße 34, A-5020 Salzburg, Austria

## Abstract

The proprotein convertase (PC) furin emerged as promising drug target for the treatment of numerous infectious diseases, cancer and cystic fibrosis. A recently described nonpeptidic lead structure served as template to develop a new series of PC inhibitors containing a dichlorobiphenyl-derived core segment decorated with a left and right inhibitor arm. The compounds were tested for their inhibitory potency against furin and the structurally related PC7. The most potent compounds inhibited furin with *K*_i_ values <5 nM, whereas most of them were significantly weaker inhibitors of PC7. Only for one compound, a significant potency with a *K*_i_ value of 7.3 nM against PC7 was found. Furthermore, crystal structures of six inhibitors in complex with furin were determined. Selected inhibitors were additionally tested for their antiviral potency against the furin-dependent H7N7 influenza A strain SC35M; a significant antiviral potency was found for compound **9**.

## Graphical abstract

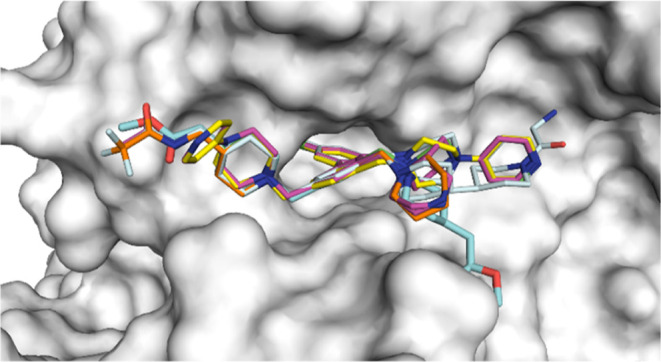

## Introduction

Furin is the most prominent member of the human proprotein convertases (PCs), a family of 9 serine proteases possessing a Ca^2+^ dependent subtilisin-like protease domain. The PCs catalyze the posttranslational maturation of many proproteins thereby contributing to a variety of physiological processes. Furin and six additional PCs (PC1, PC2, PC4, PACE4, PC5, and PC7) cleave their substrates after multibasic sequences and, therefore, are often named as basic PCs. The two other family members S1P, also known as SKI-1, and PCSK9 possess a different substrate specificity and preferably cleave after nonbasic residues.^1^ Despite an autocatalyzed cleavage between the inhibitory prodomain and the serine protease domain of PCSK9, its prodomain is not released and PCSK9 remains in an inactive state.

The host protease furin also activates numerous bacterial toxins like Shiga toxin, the protective antigen of anthrax toxin, diphtheria toxin, *Pseudomonas aeruginosa* exotoxin A or *Clostridium septicum* α-toxin.^2^ Furthermore, it cleaves several viral surface glycoproteins of pathogenic viruses, an essential step for virus propagation. For example, furin activates the hemagglutin of highly pathogenic avian influenza virus strains H5N1 and H7N1, the spike (S) protein of several corona viruses (CoV) including the S of the pandemic SARS-CoV-2, the prM precursor of the membrane protein M of all flaviviruses, like Dengue virus, West-Nile virus and Zika virus, or the F_0_ protein of measles virus, mumps virus and respiratory syncytial virus and many others.^3^ Furthermore, furin contributes to the processing of the glycoprotein GP from Marburg and Ebola virus and the cleavage of the gp160 of HIV. An activation of the gp160 of HIV was also demonstrated by PC7, consistent with furin and PC7 being the only basic PCs present in lymphatic cells and tissues.^4,5^ Furin is also described as target for the personalized treatment of certain cancer types, whereas other types of cancer do not benefit from an inhibition of furin.^6,7^ An additional furin substrate is the epithelial sodium channel (ENaC). Its activation enables the inward conductance of Na^+^ ions from the airway surface liquid (ASL) into epithelial cells. This process is normally regulated by the cAMP-dependent chloride channel cystic fibrosis transmembrane regulator (CFTR).^8^ Mutations in the gene that encodes for CFTR lead to a reduced chloride secretion and concomitant deregulation of ENaC resulting in a hyperabsorption of Na^+^ via ENaC. This causes a dehydration of the ASL and contributes to cystic fibrosis (CF).^9^ The use of highly effective furin inhibitors could therefore offer new therapeutic options for the treatment of these diseases.

Many different furin inhibitors have been described in recent years.^10^ We had focused on the development of furin inhibitors as potential antiviral agents. For this purpose, we had designed numerous substrate-analogue peptidic inhibitors of the basic PCs like inhibitor MI-1851 (Figure 1) containing C-terminal arginine mimetics such as 4-amidinobenzylamid (Amba)^11–13^ or 3-aminoisoindol-6-methylamid (Amia).^14^ Several derivatives of this type inhibit furin with K_i_ values ≤10 pM. A new type of highly effective nonpeptidic furin inhibitor has recently been described, which contain a dichlorophenylpyridine-derived core segment substituted with two inhibitor arms on the pyridine ring.^9,15^ Their crystal structures in complex with furin revealed an unexpected binding mode, where the dichlorophenylpyridine segment occupies a cryptic hydrophobic binding pocket never seen in previous crystal structures of furin that was opened by a rotation of the Trp254 side chain by nearly 180 deg.^9,16^ One of these inhibitors, BOS-318 (Figure 1), was tested in a CF model and caused a significant suppression of ENaC activation, leading to an enhanced airway hydration and thus, to an increased mucociliary clearance and improved removal of inhaled pathogens.^9^ The same inhibitor and two structurally related analogs (BOS-981 and BOS-857, exact structures undisclosed) were additionally tested for their antiviral potential against SARS-CoV-2, where they could suppress the activation of the S protein and subsequent entry of the virus into lung cells.^17^

**Figure 1:**
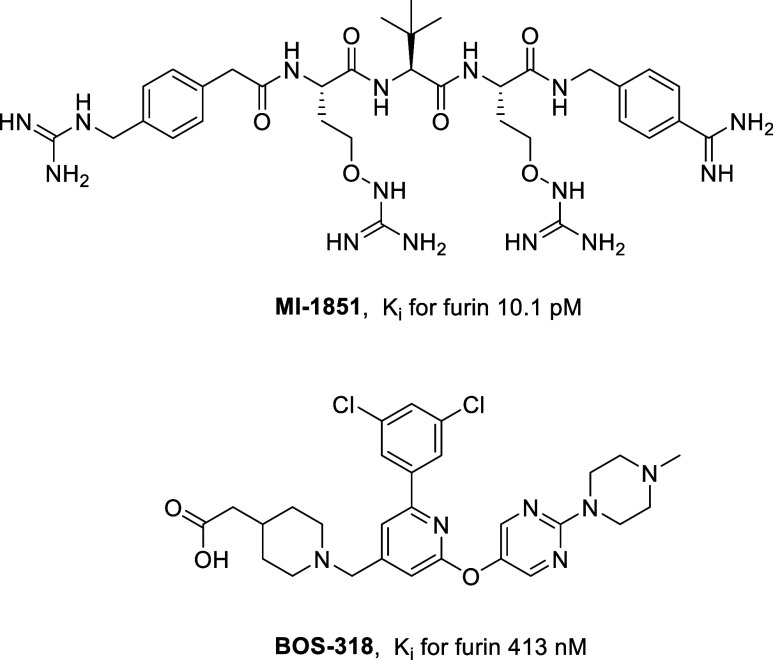
Structures of the known peptidic furin inhibitor MI-1851 ^13^ and of the nonpeptidic dichlorophenylpyridine-derived inhibitor BOS-318.^9^

Therefore, we prepared a first series with new analogs of this inhibitor type starting from a symmetrical dichlorobiphenyl segment. Their inhibitory potency was determined in enzyme kinetic studies with furin, some of them were additionally tested as inhibitors of PC7. Selected compounds have been used for crystal structure determination in complex with furin. Furthermore, their antiviral potency against the furin-dependent H7N7 influenza A strain SC35M was tested in cell culture.

## Results

### Inhibitor Design and Synthesis

The known dichlorophenylpyridine-derived inhibitors contain an aryl-oxyaryl or aryl-aminoaryl-link between the central pyridine and the first ring of the right inhibitor arm (the designation right arm is deduced from the known crystal structures of these inhibitors in complex with furin in standard orientation, whereby the right arm is directed to the east toward the catalytic triad).^9,15,16^ This suggested that it might be possible to replace the sp^3^ hybridized oxygen by a methylene group. Therefore, the biphenyl-3,5-dicarboxylic acid derivative **3** was prepared from 5-bromoisophthalic acid **1** and boronic acid **2** by Suzuki coupling (Scheme 1). As a first attempt, intermediate **3** was directly coupled with Boc-*p*-xylenediamine^18^ and after deprotection, the symmetrical diamide **4** was obtained. In analogy to a previously described synthesis of nonpeptidic PC2 inhibitors,^19^ the amides were reduced with borane-THF providing compound **5** and the singly reduced derivative **6** as minor side product (Scheme 1).

**Scheme 1:**
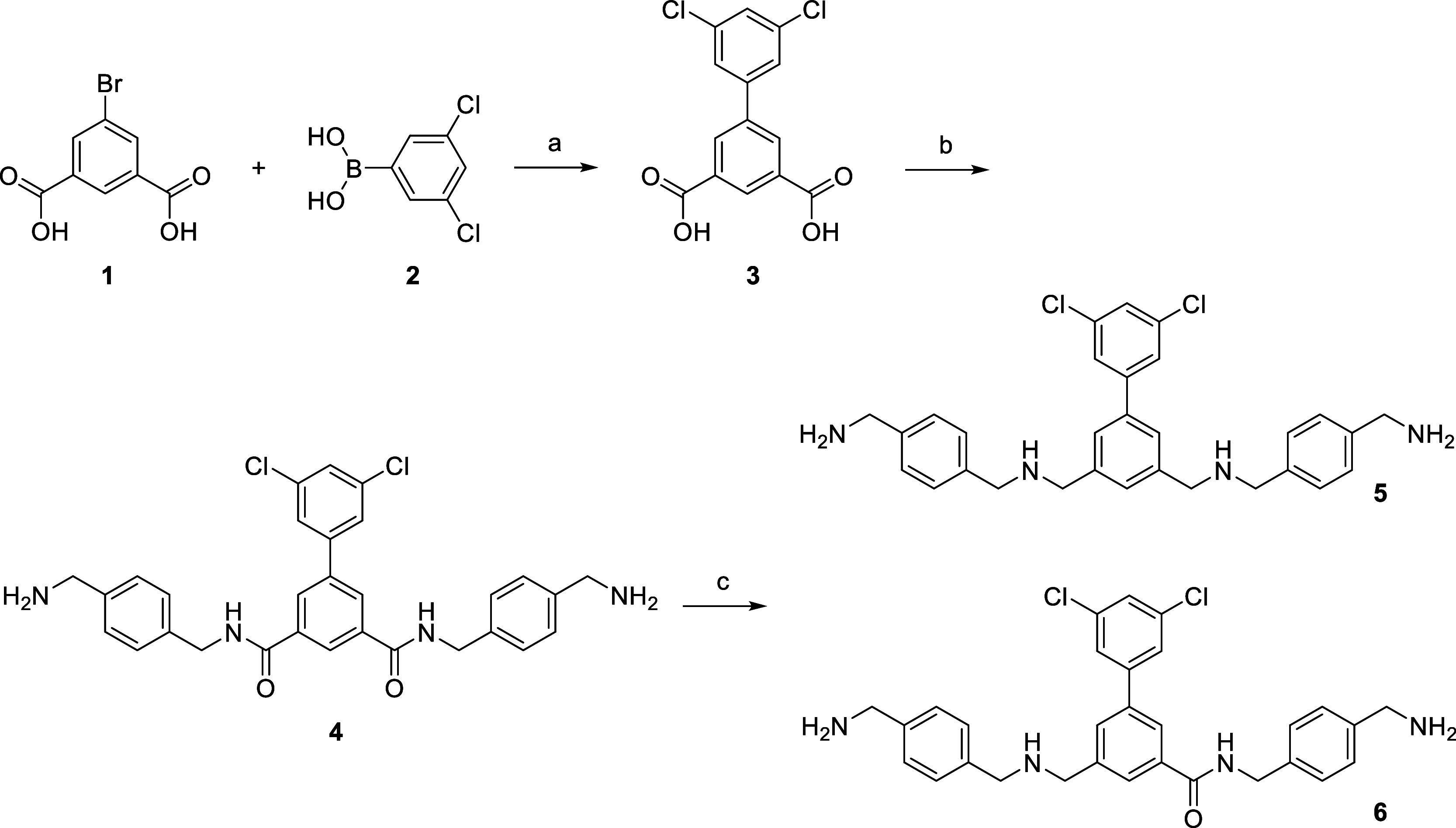
Synthesis of Compounds **4**-**6**.^a^

a Reagents and conditions: (a) 1.0 equiv of **1**, 2.2 equiv **2**, 0.03 equiv Pd­(PPh_3_)_4_, 2 equiv Na_2_CO_3_ in dioxane/water under reflux, 91% of **3**; (b) (i) 1.0 equiv of **3**, 2.4 equiv *tert*-Butyl (4-(aminomethyl)­benzyl)­carbamate, 2.4 equiv HATU, 4.8 equiv DIPEA, 3 h rt in DCM; (ii) TFA 1 h rt and preparative HPLC, 62% of **4**; (c) 1.0 equiv of **4**, 8.0 equiv BH_3_-THF in THF, 27 h at 65 °C, purification by preparative HPLC, 21% of **5** and 3% of **6**.

Compounds **4**–**6** were tested as furin inhibitors (Table 1). No inhibition was observed for the diamide **4**, whereas *K*_i_ values of 122 nM and 562 nM were determined for analogs **5** and **6** using eq 1 for reversible competitive inhibitors, respectively. This suggested that at least one amide has to be converted to a protonatable amine to achieve a furin inhibition. Most likely, this is required on the left inhibitor arm to establish a salt bridge to the side chain of Glu236, as described for the complex with inhibitor BOS-318 and related analogues.^9,16^ Due to many side products when reducing other diamides with BH_3_-THF, the synthesis strategy was changed and compound **3** was reduced to the dihydroxy-derivative **7**, which was subsequently converted into the dibromide **8** (Scheme 2). Compound **8** was used for the simultaneous alkylation of two different amines providing always two symmetrical and one asymmetrical product, because the symmetrical structure of the starting material **8** prevented the formation of two different asymmetrical products.

**Table 1:**
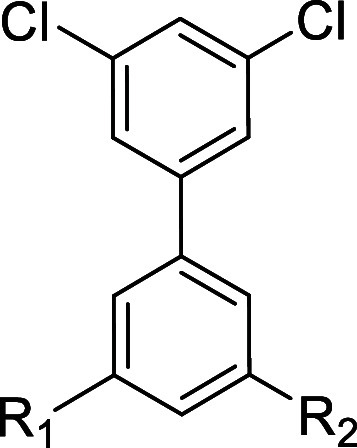

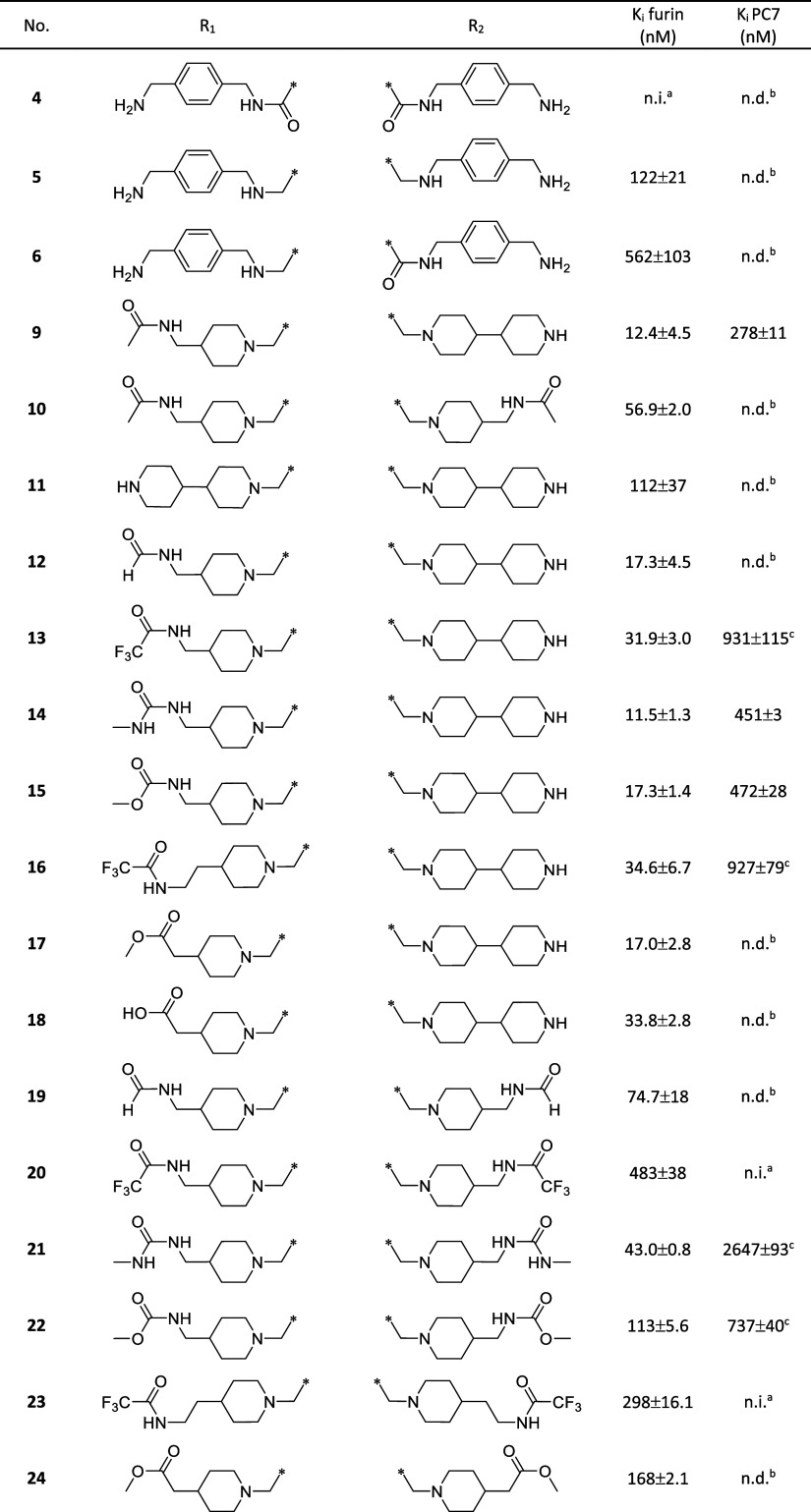

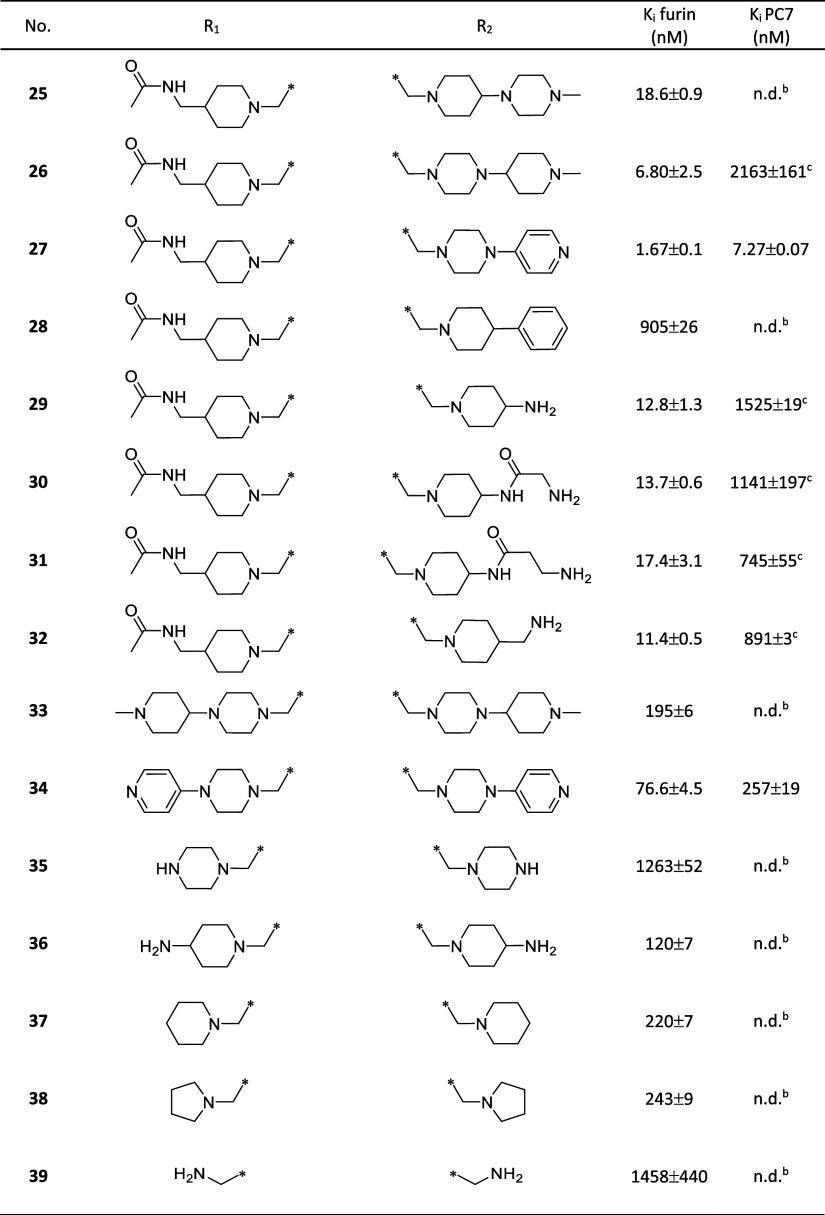
Structures and Potencies of the Synthesized Inhibitors

a n.i., no inhibition at 10 μM inhibitor concentration.

b n.d., not determined.

c Determination by preassay using inhibitor concentrations of 100 μM, 10 μM, and 1 μM for inhibitors with *K*_i_ values >700 nM.

**Scheme 2:**
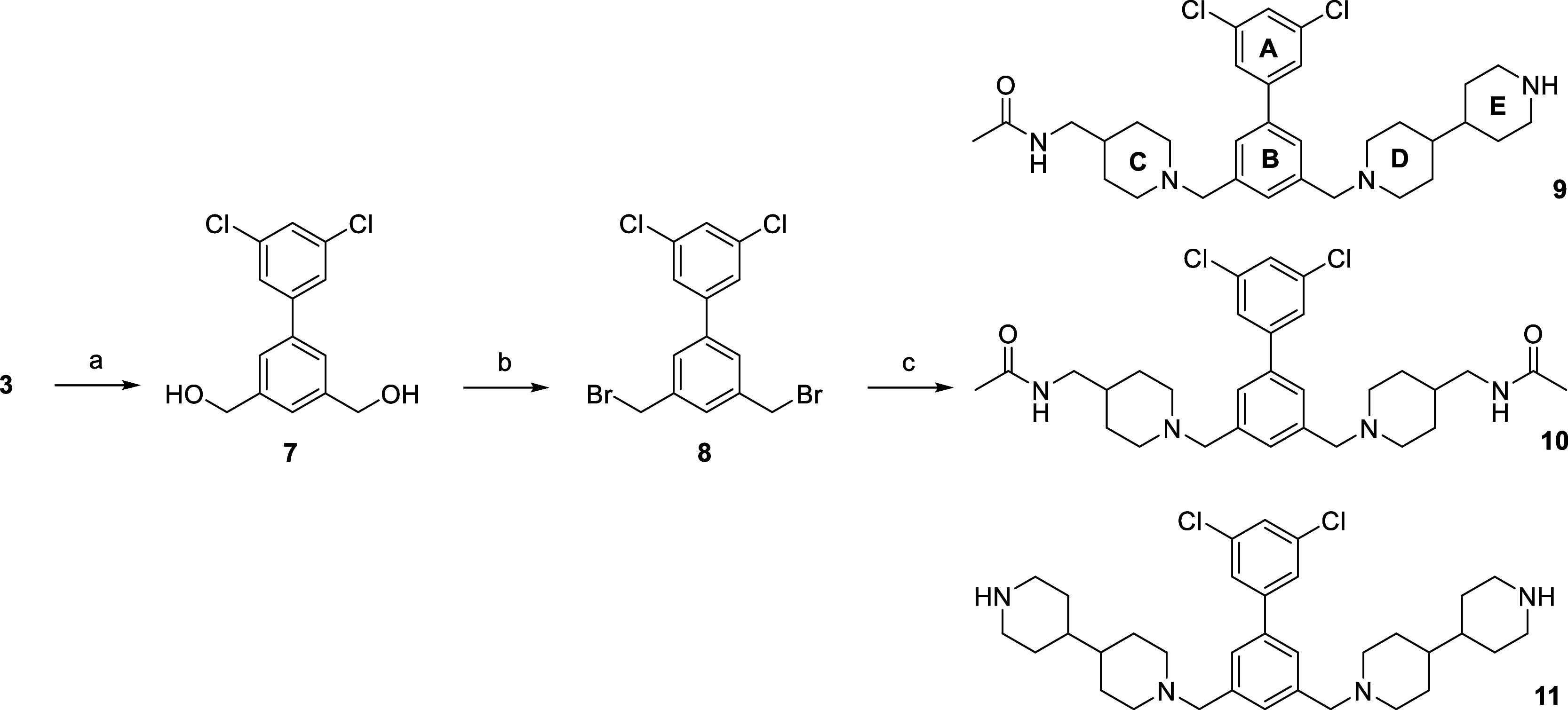
Synthesis of Inhibitors 9-11.^a^

a Reagents and conditions: (a) 1.0 equiv of **3**, 4.0 equiv BH_3_-THF in THF at 0 °C over 10 min, room temperature overnight, 89%; (b) 1.0 equiv of **7** and 1.9 equiv PBr_3_ in toluene, 1 h at 80 °C, 92% (**8**); (c) (i) 1.0 equiv of **8**, 1.0 equiv *N*-(Piperidin-4-ylmethyl)­acetamide × HCl,^15^ 1.0 equiv Boc-4,4′-Bipiperidine, 3.0 equiv DIPEA in acetonitrile, 2 h 70 °C; (ii) TFA, 1 h room temperature, precipitation in diethyl ether, preparative HPLC, 20% (**9**), 10% (**10**), 18% (**11**).

During analytical RP-HPLC analysis, the asymmetric derivative eluted always as second main product between the two symmetric derivatives modified twice with either the more hydrophilic or more hydrophobic amine, an example is given in Figure S1. When using *N*-(piperidine-4-ylmethyl)­acetamide for the incorporation of the left inhibitor arm, as known from previous compounds,^15,16^ and Boc-4,4′-bipiperidine as second amine, inhibitors **9**–**11** were obtained after removal of the Boc protecting group (Scheme 2).

Compared with inhibitor **5**, a considerably improved potency was found for the asymmetric compound **9** (*K*_i_ = 12.4 nM), whereas a reduced inhibition with K_i_ values of 56.9 nM and 112 nM were determined for the symmetric derivatives **10** and **11**, respectively. Since most asymmetric inhibitors contain five ring structures, we have named them A to E for differentiation, as shown for compound **9** in Scheme 2. The following inhibitors **12**–**34** shown in Table 1 were prepared by the same strategy through reaction of the dibromide **8** with two different amines providing one asymmetric and two symmetric products. The four symmetric compounds **35**–**38** were prepared by alkylation of intermediate **8** with only one amine (Scheme S1). The modified synthesis of analog **39** is described in Scheme S2.

Slightly reduced or similar potencies were obtained by replacing the terminal acetyl group on the left arm of inhibitor **9** by a formyl or trifluoroacetyl group, after incorporating a methylureido- or methoxycarbonylamide group and using an elongated Tfa-NH-ethyl group as substituent on ring C (**12**–**16**). The incorporation of methyl piperidylacetate and piperidylacetic acid as used in the left arm of BOS-318 provided two additional asymmetric inhibitors (**17**–**18**). The synthesis of these inhibitors provided also several symmetric compounds (**19**–**24**) possessing a reduced potency compared with inhibitor **9**. Further modifications on the right inhibitor arm yielded the asymmetric inhibitors **25**–**32** and the symmetric analogues **33**–**34**. The strongest furin inhibition was found for compound **27** containing a piperazine and 4-pyridyl group as rings D and E, respectively.

During the enzyme kinetic analysis of this inhibitor, we observed biphasic progress curves containing a pronounced initial curved segment followed by a linear steady-state part, which is typical for a slow-binding inhibition (Figure 2A). Fitting the data to eq 2 provided a first order rate constant *k*_obs_ and a steady-state velocity *v*_s_ for each progress curve. During fitting, the initial rate *v*_0_ was used as constant parameter corresponding to the rate in absence of inhibitor, which is typical for a single-step slow-binding mechanism,^20^ whereas the parameter *d* corresponds to the initial fluorescence at time point zero. The *K*_i_ value of 1.67 nM for inhibitor **27** was calculated by fitting the steady-state rates as a function of the inhibitor concentration using eq 1 (Figure 2B) and the dependence of *k*_obs_ from the inhibitor concentration provided an association rate constant *k*_on_ of 4.59 × 10^5^ M^–1^ s^–1^ (eq 3, Figure 2C). The dissociation rate constant *k*_off_ of 7.66·× 10^–4^ s^–1^ was calculated from the determined *K*_i_ and *k*_off_ values using eq 4.

**Figure 2:**
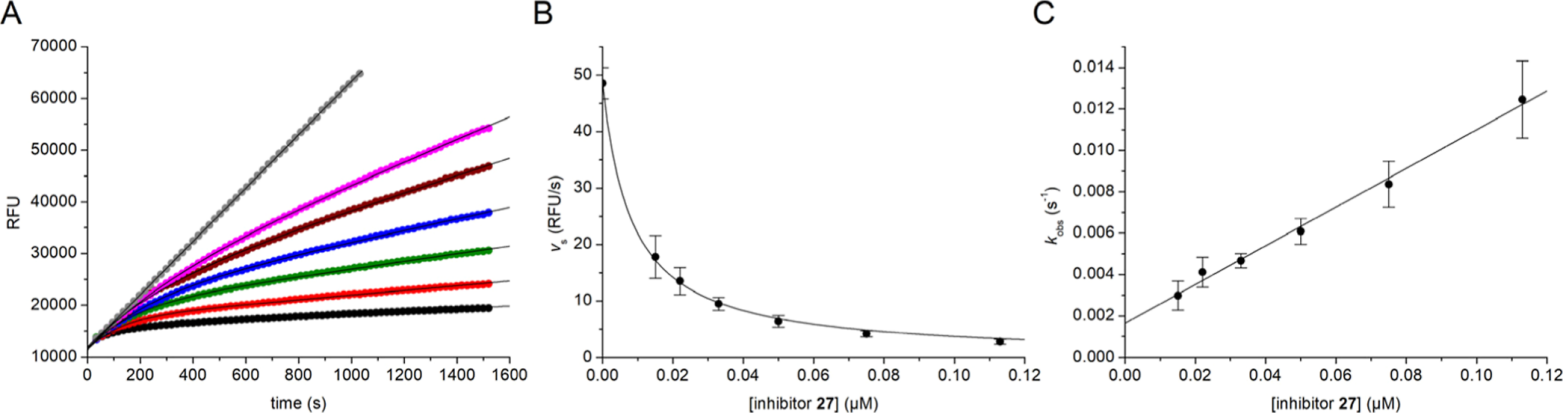
Inhibition of furin (0.95 nM) by inhibitor **27** (*n* = 5 for the data shown in panels (B,C), in panel (A) the progress curves from a single measurement are shown). (A) The progress curves were obtained in the presence of the substrate Phac-Arg-Val-Arg-Arg-AMC (25 μM) at inhibitor concentrations of 112.5 nM (black symbols), 75 nM (red), 50 nM (green), 33 nM (blue), 22 nM (brown), 15 nM (magenta) and the data were fitted to eq 2. The linear control measurement in absence of inhibitor is shown in gray. (B) The *K*_i_ value was calculated by fitting the *v*_s_ values as a function of the inhibitor concentrations with eq 1. (C) The dependence of the *k*_obs_ values from the inhibitor concentrations provided the *k*_on_ value from the slope of the fit using eq 3. The *k*_off_ value was calculated from the determined *K*_i_ and *k*_on_ values with eq 4.

A very similar *K*_i_ value for inhibitor **27** of 1.69 nM was obtained without considering the initial curved phase of the progress curves, when the *v*_s_ values were calculated from the terminal linear part of the progress curves after 1200 s. Hence, this simpler and faster method was used to calculate the *K*_i_ values of all other inhibitors, as the curved initial phases were less pronounced or not visible at all with the weaker inhibitors.

Inhibitor **9** was further modified on the terminal piperidine ring E by alkylation or acylation providing derivatives **40**–**46** (Table 2, Scheme S3). Most of these compounds inhibit furin with inhibition constants <10 nM.

**Table 2:**
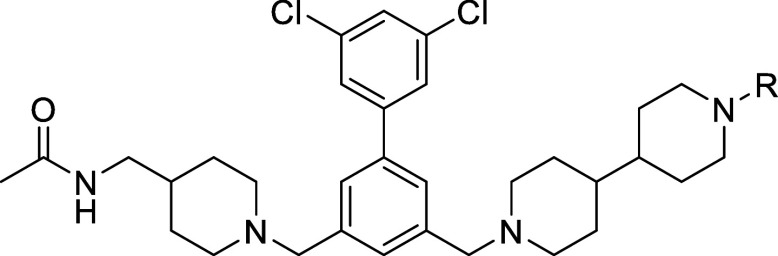

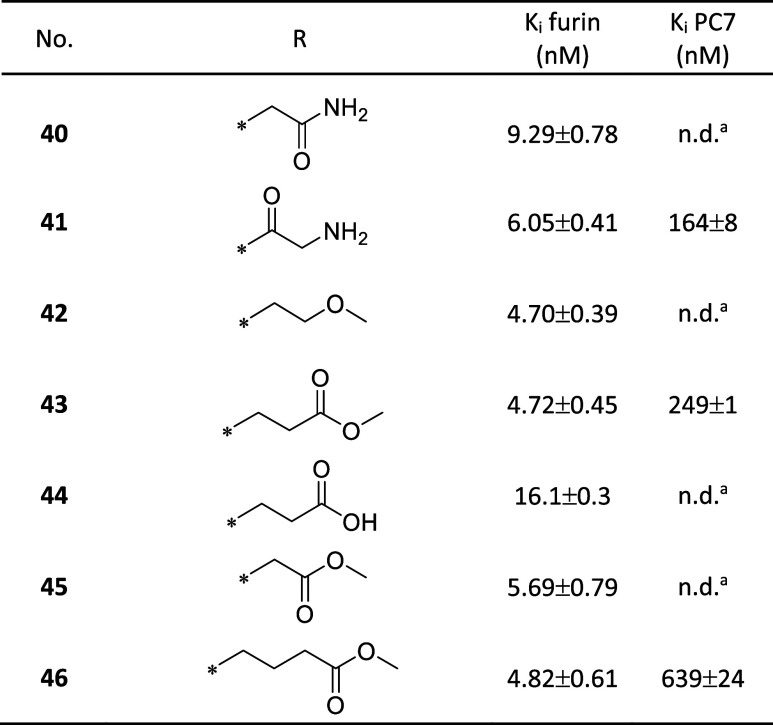
Elongated Derivatives of Inhibitor **9**

a n.d., not determined.

### Inhibition of PC7

Enzyme kinetic studies with selected inhibitors revealed that most of these compounds exhibit a weak inhibition of the related proprotein convertase PC7. A significant PC7 inhibition with a *K*_i_ value of 7.27 nM was only found for compound **27**, which was also the most potent furin inhibitor. All other tested compounds possess inhibition constants >150 nM for PC7. Since no crystal structure for PC7 is yet available, we cannot explain why only compound **27** shows a significant inhibition of this protease. However, due to high sequence similarity between both proprotein convertases, PC7 should be inhibited by a similar binding mode as described for furin in the following paragraph.

### Crystal Structures

The binding mode of selected symmetric (**24**, **34**) and asymmetric inhibitors (**13**, **26**, **27**, **41**) was determined by X-ray crystallography after soaking into crystals of ligand-free furin. In Table S1, the statistics of the data collection and refinement of the complexes are summarized. The central segment with ring A and B all well as the left arm with ring C was well-defined in the electron density map of all investigated inhibitors (Figure S3). In all complexes, the central dichlorobiphenyl-segment occupied a hydrophobic pocket in a similar manner, as described for furin in complex with various dichlorophenylpyridine-derived inhibitors.^9,16^ This cryptic binding pocket was opened after a flip of the side chain of the furin residue Trp254 by approximately 180° and lined by the hydrophobic side chains of furin residues Leu152, Met226, Leu227, Val231, Leu240, Ala252 and Trp291 (Figure S2). Additional contacts found in all complexes were a characteristic salt bridge between the piperidyl nitrogen on the left inhibitor arm (ring C) and the carboxylate of Glu236, as well as a polar interaction from the same nitrogen to the hydroxy group of Tyr308.

Well-defined binding modes were found for the two symmetric inhibitors **24** (Figure 3A) and **34** (Figure 3B). Inhibitor **24** contains two piperidinyl-methyl acetate arms. Its carbonyl oxygen on the left arm was bound to the carboxylate of Asp264 via a bridging water molecule, whereas the right arm was oriented downward thereby enabling two polar contacts from its carbonyl oxygen to the amide NH of Asp258 and to the carbonyl oxygen of Gly255 via a water molecule. An additional water was bound to the piperidinyl nitrogen of the right arm.

**Figure 3:**
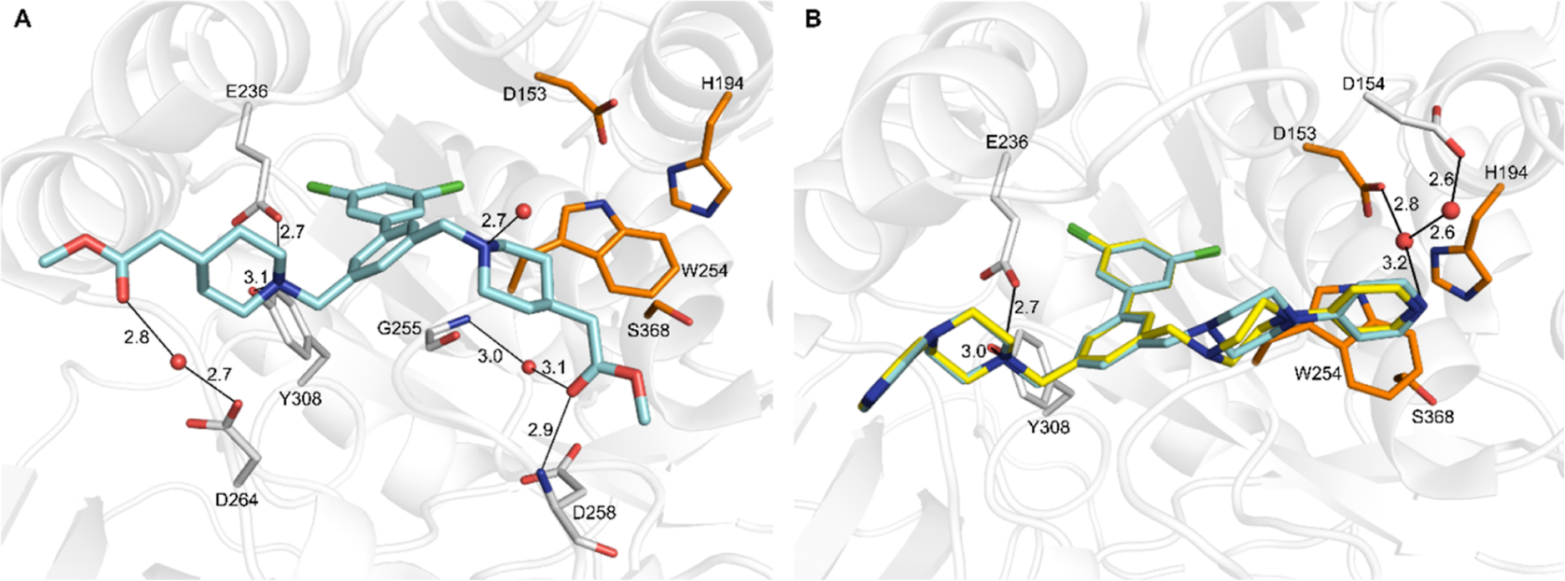
Crystal structures of furin in complex with the symmetric inhibitors (A) **24** (PDB: 9QWG) with carbon atoms in cyan and (B) **34** (PDB: 9QWD), which was modeled in two conformations (carbon atoms in yellow and cyan). These conformations differ only in the orientation of the piperazine ring on the right inhibitor arm. Furin residues involved in polar contacts (black lines) are shown with carbons in white. The side chains of the residues of the catalytic triad (Ser368, His194, and Asp153) and of Trp254 are shown with carbons in orange for orientation, water molecules are given as red spheres.

In contrast, the dichlorobiphenyl-core of inhibitor **34** is substituted with two 4-pyridyl-piperazine groups and was modeled in two conformations (Figure 3B). The piperazine ring on the left arm was similarly placed as the piperidine ring C of inhibitor **24**, whereas the 4-pyridyl group was oriented into the solvent and not involved in any contacts to furin. The piperazine on the right arm occupied a different position compared to the piperidine ring of inhibitor **24**. The different conformations of the piperazine ring D of inhibitor **34** indicate high structural flexibility while the position of its terminal pyridyl group was always nearly identical. The terminal ring E mediated a polar contact to the side chains of Asp153 and Asp154 via a water network. Furthermore, the pyridyl group was positioned nearly perpendicular above the indole side chain of Trp254 making an edge to face interaction between these ring systems.

Binding modes could be also determined for the asymmetric inhibitors **13**, **26**, **27**, and **41**. The electron densities of the central biphenyl core and the structurally similar left arms of inhibitors **13** and **26** were well-defined. The trifluoroacetamide and acetamide groups were involved in two identical water mediated polar contacts between their amide NH and the carboxyl group of Asp264 and its carbonyl oxygen to the backbone NH of Asp233 (Figure 4). In contrast, in both structures a sufficient electron density could be only obtained for the first ring D of the right inhibitor arm (Figure S3A,C). This suggests a high flexibility of the terminal ring E in these inhibitors.

**Figure 4:**
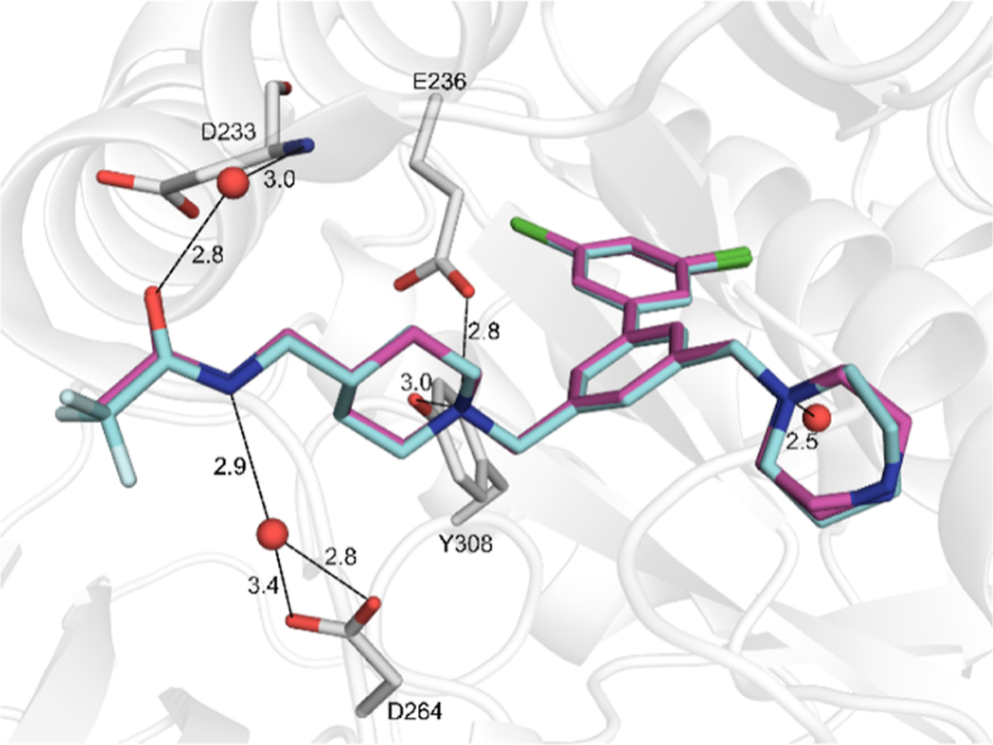
Crystal structures of furin in complex with the asymmetric inhibitors **13** (carbon atoms in cyan, PDB: 9QWF) and **26** (carbons in magenta, PDB: 9QWB). The inhibitors are shown as superposition. The furin was taken from the complex with inhibitor **13** and is shown as transparent cartoon. Furin residues involved in polar contacts (black lines, taken from the complex with inhibitor **13**) are given as sticks with white carbon atoms. In both complexes, no electron density could be found for the terminal ring on the right inhibitor arm (Figure S3A,C). The bound conformation of both inhibitors was nearly identical.

In the complex with the most potent inhibitor **27** (Figure 5), the left arm was placed as described above for compound **26** (Figure 4), its acetamide group and the nitrogen of the piperidyl ring made identical interactions. Again, two conformations were observed for the right inhibitor arm, similar as seen with the symmetric compound **34** (Figure 3B) containing an identical 4-pyridyl-piperazine group on this side. In both conformations, the position of the terminal pyridyl ring was very similar to that described for inhibitor **34**, its edge was placed over the indole of Trp254. However, slight differences existed in the water network. Here, three water molecules were found and the distance of the pyridyl nitrogen to the first water (3.4 Å) was somewhat longer suggesting a relatively weak polar contact. This water also interacts with the side chain of Asp153 from the catalytic triad and a second water, which is involved in contacts to the side chain of Asp154, the carbonyl oxygen of Asp191, and a third bridging water that binds to the side chain of Asn192 (Figure 5).

**Figure 5:**
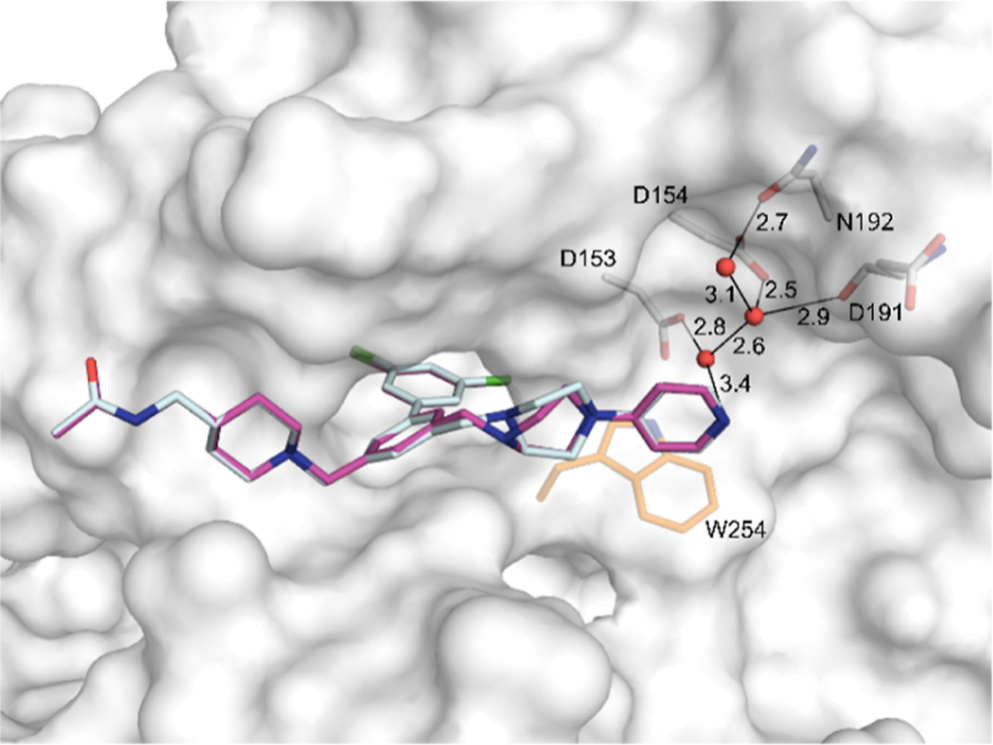
Crystal structure of furin shown with a transparent surface in yellow orange in complex with inhibitor **27** (PDB: 9QWE), which was refined in two conformations (carbon atoms in pale cyan or magenta) differing only in the placement of the piperazine ring D. The contacts of the left inhibitor arm are not shown for simplicity, as they are identical to the structures with inhibitors **13** and **26** described above in Figure 4. The furin residues involved in contacts to the terminal pyridyl group via a network of three water molecules (red spheres) are shown as sticks with carbons in yellow. Polar contacts are shown as black lines, distances are given in Å. The side chain of Trp254 is shown for orientation.

The acylation of the bipiperidyl group of inhibitor **9** with glycine stabilized a single conformation of inhibitor **41** in complex with furin leading to a direct salt bridge between its terminal amino group and the side chain of Asp154 (Figure 6). The same amino group is further involved in polar contacts to the backbone carbonyl of Asp191 and bridging water molecules, mediating polar contacts to the carbonyl oxygen of Leu227 and the side chain carbonyl of Asn192. In addition, a complex water network was found below the inhibitor between the acetamide of the left inhibitor arm and the piperidyl nitrogen of ring D on the right arm involving residues Asp264, Thr262, and Glu257. The ring E of inhibitor **41** is positioned on top of the displaced Trp254 and mediates CH−π-interactions with its indole ring system.

**Figure 6:**
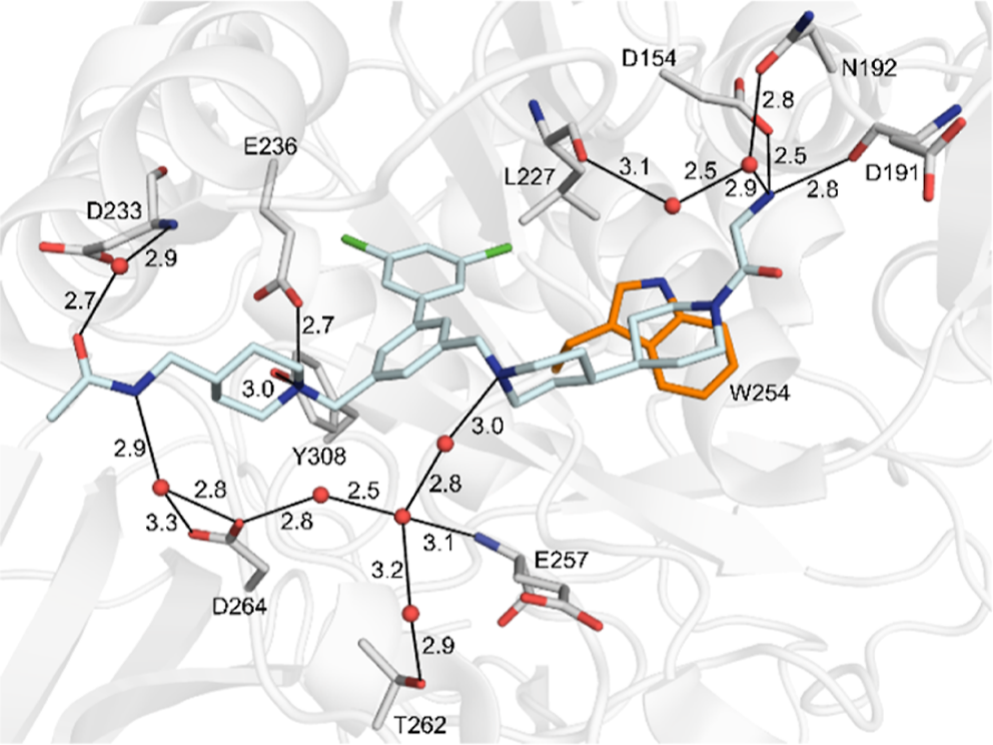
Crystal structure of furin in complex with inhibitor **41** (PDB: 9QWC). The protease and the inhibitor are shown as cartoon (white) and stick model (carbons in cyan), respectively. Furin residues involved in polar contacts (black lines, distances given in Å) are labeled and shown as sticks, Trp254 is additionally provided for orientation. Water molecules are given as red spheres.

### Antiviral Activity

Furin cleaves and thereby activates surface glycoproteins of numerous pathogenic viruses, e.g., the hemagglutinin (HA) of highly pathogenic avian influenza A viruses (HPAIV). The HA cleavage is required for the fusion of the virus membrane with host cell membranes within endosomes and crucial for virus replication. HPAIV infections are the cause of bird flu (fowl plague), which can cause enormous damage in chicken populations. So far, only very few HPAIV infections of humans with the subtype H5N1 occurred when they had direct contact with infected birds. The mouse adapted SC35M is a different HPAIV strain of the subtype H7N7, which was originally isolated from seals on the coast of New England in 1980. It efficiently replicates in mammalian cells and is a suitable model strain to test the antiviral efficacy of furin inhibitors, as done previously with our peptidic inhibitors.^14^ Therefore, we used the same system to test the antiviral activity of the newly synthesized nonpeptidic inhibitors. At the beginning, nearly all compounds with inhibition constants <20 nM have been screened at a single concentration of 5 μM and the most promising five compounds were further tested at different concentrations. Notably, at the used inhibitor concentrations of ≤5 μM, a slight cytotoxic effect on the A549 cells after 72 h incubation was only observed for inhibitor **27** (Figure S4). After infection by SC35M, A549 cells were incubated in the presence of these inhibitors for 72 h. Cell supernatants were collected at different time points and their virus titers determined by plaque formation assay (Figure 7A–E). The peptidic inhibitor MI-1851 (4-(guanidinomethyl)­Phac-Cav-Tle-Cav-4-amidinobenzylamide, originally described as compound 8 in our previous publication^13^), structure shown in Figure 1 served as reference compound (Figure 7F).

**Figure 7:**
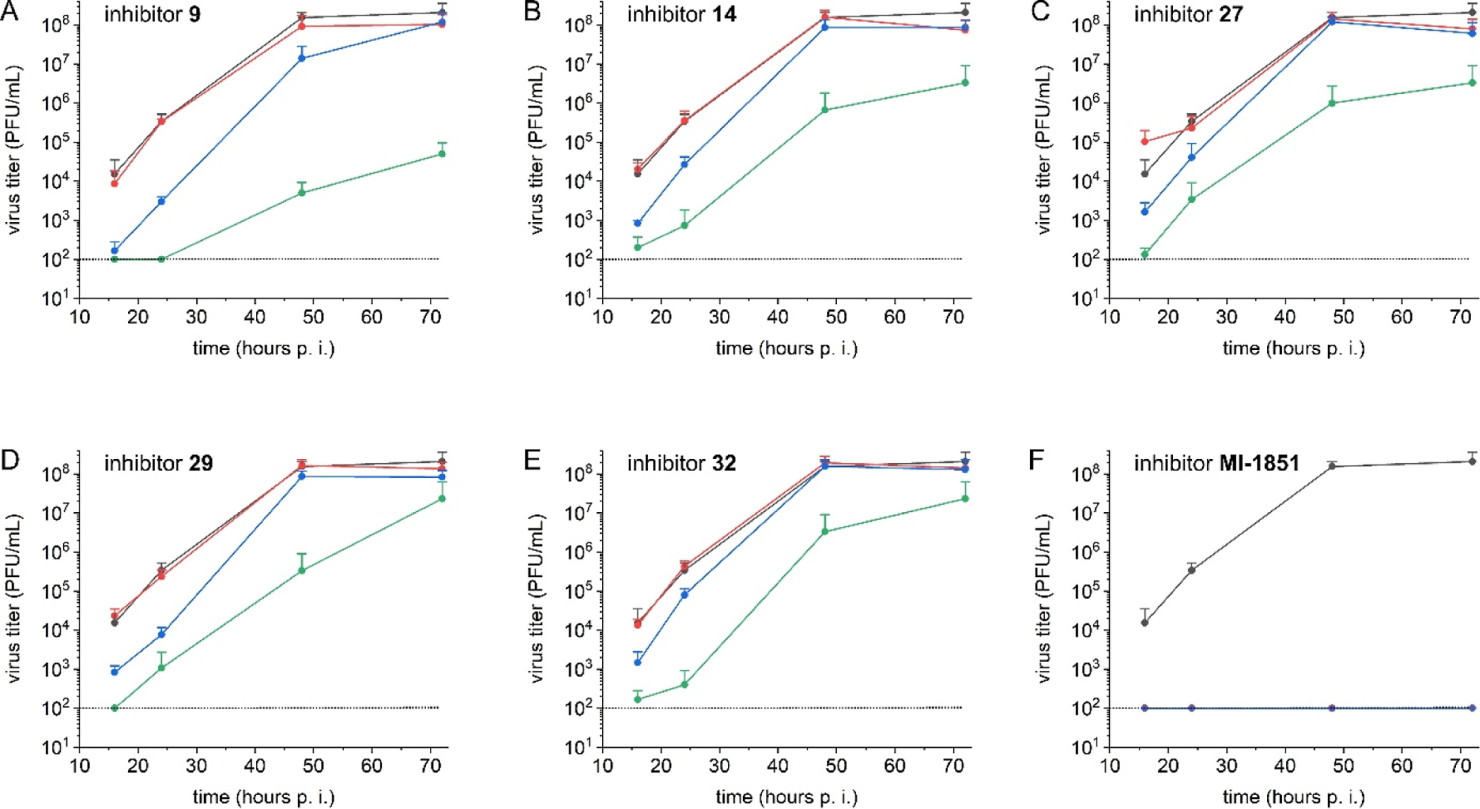
Multicycle replication of influenza A strain SC35 M in inhibitor-treated A549 human lung cells. The cells were inoculated with virus at a MOI of 0.0001 for 1 h, washed and incubated in the presence of selected inhibitors (panels (A–E) for inhibitors **9**, **14**, **27**, **29**, and **32**) at the indicated concentrations ((●) control in absence of inhibitor, (●—red) 1 μM, (●—blue) 2.5 μM, and (●—green) 5 μM) for 72 h. At 16, 24, 48, and 72 h postinfection (p.i.) cell supernatants were collected and viral titers were analyzed by plaque formation assay. Data are mean values +SD of three independent experiments. The dotted line at 10^2^ PFU/mL represents the limit of detection in this assay. In case of the control inhibitor MI-1851 (F), a complete inhibition of virus replication was observed at the two lower concentrations of 2.5 μM (●—blue) and 1 μM (●—red), therefore, the higher concentration of 5 μM was not tested.

At the lowest concentration of 1 μM, we did not observe an antiviral effect of the selected inhibitors against SC35M. At the next higher dose of 2.5 μM, a significant inhibition was obtained at the two earlier time points at 16 and 24 h post infection (h p.i.), whereas after 48 and 72 h p.i. the virus titers were similar as found for the control. In contrast, over the whole period of 72 h a significant inhibition of virus replication was found for all nonpeptidic inhibitors at a concentration of 5 μM. The strongest antiviral efficacy was found for compound **9**, which reduced the virus titers nearly 100′000- and 10′000-fold after 48 and 72 h, respectively. However, the antiviral efficacy of these new inhibitors was significantly weaker when compared with our previously described peptidic reference inhibitor MI-1851, which yielded a complete inhibition at concentrations of 1 and 2.5 μM.

## Discussion and Conclusions

Furin has been recognized for many years as a potential target for the treatment of infectious diseases, various types of cancer and cystic fibrosis.^1,6,9^ Initial efforts for the development of furin inhibitors were focused on the design of peptidic substrate-analog structures. Many of these multibasic compounds inhibit furin in the low picomolar range, but usually require concentrations around 0.5–10 μM in order to achieve a significant antiviral efficacy against furin-dependent viruses in cell culture.^12,13^ Since furin cleaves most substrates, including the viral glycoproteins, in the TGN, it was assumed that only very small amounts of these peptidic structures can reach the intracellular furin, resulting in the large discrepancy between the effectiveness of the inhibitors in enzyme kinetic measurements and cell culture studies. Peptides often serve as starting point for the design of nonpeptidic compounds, which often possess an improved bioavailability. In the field of furin inhibitors,^10^ the 2,5-dideoxystreptamine derivatives substituted with several guanidine groups were the first potent small molecule inhibitors possessing *K*_i_ values in the low nanomolar range.^21^ However, their strongly basic character was similar to that of the peptidic inhibitors, which limited their further development. The recently described dichlorophenylpyridine-derived PC inhibitors, for which a significant cell permeability was shown in initial studies,^9,17^ are a more promising lead structure for drug development. For this reason, we have developed a new inhibitor series with a similar central dichlorobiphenyl group, where both inhibitor arms are linked via a methylene group to this core segment. In comparison to the more complex synthesis of the described dichlorophenylpyridine derivatives,^9,15^ this modification allowed the rapid synthesis of numerous derivatives according to Scheme 2, although we always obtained a mixture of the desired asymmetric inhibitor and two symmetric byproducts, which limits the yield of the reaction. However, this is not an issue at this early stage of development, as these compounds could usually be easily separated from each other by preparative RP-HPLC, either at the stage of the protected intermediates or at the final step, after the removal of protecting groups (Figure S1).

For the most potent inhibitor **27** of this series, which inhibits furin with an inhibition constant of approximately 1.7 nM according to a slow-binding mechanism, we could determine the individual rate constants *k*_on_ and *k*_off_ by enzyme kinetic measurements. Compared to the inhibitor BOS-318 (*K*_i_ = 0.431 nM, *k*_on_ = 2.15 × 10^5^ M^–1^ s^–1^, *k*_off_ = 4.38 × 10^–5^ s^–1^),^9^ which is an approximately 4-fold stronger furin inhibitor, the association rate constant of compound **27** is approximately twice as high (*k*_on_ = 4.59 × 10^5^ M^–1^ s^–1^), but in a similar range. We assume that the association step of these inhibitors is primarily influenced by the flexibility of the Trp254 side chain and the rate of its flip to the outside, which opens a cryptic hydrophobic binding pocket essential for the binding of these structures. This spontaneous conformational change in the active site of furin should be rather independent of the specific structure of these nonpeptide inhibitors, leading to an equilibrium between the standard conformation of ligand-free furin with the S1 pocket accessible for substrate binding^22^ and a second conformation with the open cryptic binding pocket. Only this second furin conformation is suited to bind the dichlorophenyl group of the nonpeptide inhibitors, while substrate-analog structures address the standard conformation.

In contrast, larger differences exist in the dissociation rate constants of the furin complexes with inhibitors **27** and BOS-318. The different *k*_off_ values suggest that the furin complex with inhibitor BOS-318 is significantly more stable compared to the complex with inhibitor **27** (*k*_off_ = 7.66 × 10^–4^ s^–1^). This results in a more than 10-fold longer residence time of approximately 380 min (calculated as 1/*k*_off_ ^23,24^ ) for BOS-318 compared to approximately 22 min for inhibitor **27**. This is still a disadvantage of compound **27**, because several reports suggest that the pharmacological efficacy of a drug correlates better with a longer residence time than with its inhibition constants.^23^ Further studies are therefore needed to investigate whether more suitable nucleophilic structures can be coupled as right inhibitor arm to the bis-bromide intermediate **8** (Scheme 2).

The determined six structures of the furin/inhibitor complexes revealed a constant binding mode for the central biphenyl scaffold and the left inhibitor arm, which is very similar to that observed in the crystal structure with inhibitor BOS-318 ^9^ and its analogs.^16^ In contrast, a considerable flexibility was found for the right inhibitor arm, especially the first ring D was oriented in different directions. In two complexes with inhibitors **13** and **26**, a clear electron density was lacking for the terminal ring E. However, the coupling of a glycine residue on the terminal piperidine ring of inhibitor **9** led to a fixation of the right inhibitor arm in case of inhibitor **41** due to the formation of polar contacts from the protonated amino group of this glycine to the side chain of Asp154, to the carbonyl oxygen of Asp191, and via a bridging water to the side chain of Asn192 (Figure 6). These three additional contacts provide only a moderate 2-fold improved furin potency of inhibitor **41** (*K*_i_ = 6.05 nM) compared with analog **9** (*K*_i_ = 12.4 nM). Somewhat surprising, a further 4-fold stronger furin inhibition was found for compound **27** (*K*_i_ = 1.72 nM), although only a single and relatively long polar contact (3.4 Å) was found between the terminal pyridine nitrogen to a surrounding water molecule, which mediates a contact to the Asp153 side chain. Notably, this water belongs to a more complex water network involved in additional polar contacts to furin (Figure 5). Probably, also the edge to face contact between the terminal pyridine ring to the indole of Trp254 contributes to the stronger binding activity of inhibitor **27**. Inhibitor **27** is also the only compound of this series, which possesses a considerable inhibitory potency against the related PC7 with a *K*_i_ value <10 nM.

As a model system, the mouse adapted HPAIV strain SC35M was used to determine the antiviral potency of these compounds in infected human airway epithelial cells. In this assay, inhibitor concentrations between 2.5 and 5 μM were necessary to obtain significant antiviral effects. However, we found a stronger effect when using our previously described peptidic reference inhibitor MI-1851 (Figure 7). In our previous study with the SC35M strain, the inhibitor MI-1851 showed a significant antiviral effect even at a low concentration of 0.1 μM.

In summary, we were able to synthesize new nonpeptidic furin inhibitors. The best compounds from this series inhibit furin with inhibition constants below 5 nM. Furthermore, we could determine the binding mode of several inhibitors in complex with furin. However, initial experiments investigating the antiviral efficacy in cell culture against the influenza strain SC35M required inhibitor concentrations in the range of 2.5–5 μM to achieve significant effects. Therefore, these new nonpeptidic compounds are, at least in this assay, less effective than our previously described peptidic inhibitors. Nonetheless, this nonpeptidic inhibitor type offers numerous opportunities for a further optimization.

## Experimental Section

### General

Reagents and solvents were obtained from Alfa Aesar (Kandel, DE), BLDPharm (Kaiserslautern, DE), Carbolution Chemicals (St. Ingbert, DE), Carl Roth (Karlsruhe, DE), Thermo Fisher Scientific (Dreieich, DE), Fluorochem (Frankfurt, DE), Iris Biotech (Marktredwitz, DE), Merck KGaA (Darmstadt, DE), TCI (Eschborn, DE) or VWR international (Darmstadt, DE) and were used without further purification. Analytical HPLC measurements were performed on a Primaide system (VWR, Hitachi, column: NUCLEODUR C18 ec, 5 μm, 100 Å, 4.6 mm × 250 mm, Macherey-Nagel, Düren, Germany) with 0.1% TFA in water (solvent A) and 0.1% TFA in acetonitrile (solvent B) as eluents using a linear gradient with an increase of 1% solvent B/min at a flow rate of 1 mL/min and detection at 220 nm. Purifications via preparative HPLC were performed on a Knauer Azura system (pump P 2.1 L equipped with pump head E4099AB, detector UVD 2.1L, Knauer GmbH, Berlin, Germany, column: NUCLEODUR C18 ec, 5 μm, 100 Å, 32 mm × 250 mm, Macherey-Nagel, Düren, Germany) using the same solvents as described above for analytical HPLC and a linear gradient with an increase of 0.5% solvent B/min at a flow rate of 20 mL/min (detection at 220 nm, in few cases with larger amounts at 254 nm). After preparative HPLC, the inhibitors were obtained as lyophilized TFA-salts in a purity >95% (based on the analytical HPLC detection at 220 nm) using a freeze-drier (Martin Christ GmbH, Osterode am Harz, Germany). Certain inhibitors were converted into their less hygroscopic HCl salts by thrice lyophilization from aqueous 100 mM HCl (about 4–5-fold excess of HCl for each lyophilization step), followed by lyophilization from deionized water.^25^ ESI mass spectra were measured on a QTrap 2000 ESI spectrometer (Applied Biosystems, now part of Thermo Fischer Scientific), HR-ESI mass spectra were determined using a micrOTOF-Q III spectrometer (Bruker Daltonics, Billerica, MA), HR-APCI mass spectra were measured with a LTQ FT Ultra mass spectrometer (Thermo Fischer Scientific), and HR-EI mass spectra were acquired with a AccuTOF GCv 4G (JEOL) Time of Flight (TOF) mass spectrometer. An internal or external standard was used for drift time correction. NMR-spectra were measured on an ECA500 (^1^H: 500 MHz, ^13^C: 126 MHz) spectrometer (Jeol Germany, Freising, Germany) using the respective deuterated solvents as internal standard.

### Synthesis

The synthesis of all compounds shown in Schemes 1 and 2 is described below, as well as the synthesis of the most potent inhibitor **25** from Table 1 and of compounds **41**–**44** from Table 2. The synthesis of all other precursors and inhibitors is described in the Supporting Information.

#### 3′,5′-Dichloro-[1,1′-biphenyl]-3,5-dicarboxylic Acid (**3**)

A solution of 5-bromoisophthalic acid (2.00 g, 8.16 mmol, 1 equiv), (3,5-dichlorophenyl)­boronic acid (1.87 g, 9.79 mmol, 1.2 equiv), and Na_2_CO_3_ (1.73 g, 16.32 mmol, 2 equiv) in a mixture of 20 mL water and 70 mL 1,4-dioxane was treated with Pd­(PPh_3_)_4_ (0.283 g, 0.24 mmol, 0.03 equiv) under an argon atmosphere. The suspension was stirred 1 h under reflux. Because of incomplete conversion (reaction control by HPLC), the suspension was treated with additional (3,5-dichlorophenyl)­boronic acid (1.56 g, 8.16 mmol, 1 equiv) and the mixture was further stirred for 5 h under reflux. The suspension was filtered and the basic filtrate was washed three times with EtOAc. The aqueous layer was acidified with concentrated HCl, the precipitated product was isolated by centrifugation, washed twice with water and dried in vacuo. Yield: 2.311 g (7.43 mmol, 90.9%) of compound **3** as a colorless solid. HPLC: 35.3 min, start at 20% B (purity 83.0%). ^1^H NMR (500 MHz, DMSO-*d*_6_): δ = 13.42 (s, 2H), 8.50 (t, *J* = 1.5 Hz, 1H), 8.39 (d, *J* = 1.5 Hz, 2H), 7.83 (d, *J* = 1.7 Hz, 2H), 7.68 (t, *J* = 1.7 Hz, 1H) ppm. ^13^C NMR (126 MHz, DMSO-*d*_6_): δ = 166.25, 142.04, 138.38, 134.82, 132.23, 131.74, 129.76, 127.69, 125.87 ppm. MS (ESI, negative): calcd, 309.98; *m*/*z*, 309.19 [M – H]^−^.

#### *N* ^3^,*N* ^5^-Bis­(4-(aminomethyl)­benzyl)-3′,5′-dichloro-[1,1′-biphenyl]-3,5-Dicarboxamide × 2 TFA (**4**)

Compound **3** (80 mg, 0.257 mmol, 1.0 equiv) and *tert* butyl (4-(aminomethyl)­benzyl)­carbamate × HCl (168 mg, 0.617 mmol, 2.4 equiv) were dissolved in 10 mL DCM and treated with 235 mg (0.617 mmol, 2.4 equiv) HATU and 210 μL (1.24 mmol, 4.8 equiv) DIPEA. The mixture was stirred for 3 h at room temperature. The solvent was removed in vacuo and the residue was treated with 3 mL of TFA and stirred for 1 h at room temperature. After precipitation in cold diethyl ether, the crude product was obtained by centrifugation, purified by preparative HPLC and lyophilized. Yield: 123 mg (0.159 mmol, 62%) of product **4** as a colorless solid. HPLC: 20.92 min, start at 20% B (purity >99%). ^1^H NMR (500 MHz, DMSO-*d*_6_): δ = 9.33 (t, *J* = 6.0 Hz, 2H), 8.45 (t, *J* = 1.6 Hz, 1H), 8.37 (d, *J* = 1.6 Hz, 2H), 8.14 (s, 6H), 7.93 (d, *J* = 1.9 Hz, 2H), 7.70 (t, *J* = 1.8 Hz, 1H), 7.43–7.38 (m, 8H), 4.55 (d, *J* = 5.9 Hz, 4H), 4.02 (s, 4H) ppm. ^13^C NMR (126 MHz, DMSO-*d*_6_): δ = 165.3, 142.4, 139.9, 137.4, 135.3, 134.8, 132.4, 128.8, 128.0, 127.5, 127.4, 127.2, 125.7, 66.9, 42.5, 42.0, 31.3 ppm and signals of TFA: 157.8 (q, *J* = 31.1 Hz) ppm. HRMS (APCI, positive): calcd, 546.1589; *m*/*z*, 547.1663 [M + H]^+^.

#### 1,1′-(3′,5′-Dichloro-[1,1′-biphenyl]-3,5-diyl)­bis­(*N*-(4-(aminomethyl)­benzyl)­methanamine) × 4 TFA (**5**)

Compound **4** (122 mg, 0.157 mmol, 1.0 equiv) was dissolved under argon atmosphere in 1 mL of dry THF and treated dropwise with 1 M BH_3_-THF solution (1.260 mL, 1.260 mmol, 8.0 equiv). The mixture was heated to 65 °C and stirred for 27 h. After the addition of 2 mL water/THF (1:1, v/v), the solvent was removed in vacuo and the crude product purified by preparative HPLC. Yield: 31.4 mg (0.032 mmol, 21%) of product **5** as a colorless lyophilized solid. HPLC: 21.78 min, start at 10% B (purity >99%). ^1^H NMR (500 MHz, DMSO-*d*_6_): δ = 9.64 (s, 4H), 8.31 (s, 6H), 8.01–7.92 (m, 2H), 7.77 (d, *J* = 1.9 Hz, 2H), 7.70 (t, *J* = 1.9 Hz, 1H), 7.61–7.58 (m, 1H), 7.57–7.45 (m, 8H), 4.38–4.12 (m, 8H), 4.10–4.02 (m, 4H) ppm. ^13^C NMR (126 MHz, DMSO-*d*_6_): δ = 142.4, 137.5, 134.9, 134.8, 133.0, 132.0, 131.9, 130.1, 129.0, 129.0, 127.5, 125.2, 49.8, 49.7, 41.8 and signals of TFA at 158.2 (q, *J* = 31.7 Hz) ppm. HRMS (ESI, positive): calcd, 518.2004, *m*/*z*, 519.2064 [M + H]^+^.

#### *N*-(4-(Aminomethyl)­benzyl)-5-(((4-(aminomethyl)­benzyl)­amino)­methyl)-3′,5′-dichloro-[1,1′-biphenyl]-3-carboxamide × 3 TFA (**6**)

Compound **6** was isolated as a byproduct during the preparative HPLC purification of compound **5**. Yield: 4.1 mg (0.005 mmol, 3%) of compound **6** as a colorless lyophilized solid. HPLC: 25.85 min, start at 10% B (purity >99%). ^1^H NMR (500 MHz, DMSO-*d*_6_): δ = 9.45 (s, 2H), 9.28 (t, *J* = 5.9 Hz, 1H), 8.42–7.98 (m, 9H), 7.86 (d, *J* = 1.9 Hz, 2H), 7.70 (t, *J* = 1.9 Hz, 1H), 7.57–7.48 (m, 4H), 7.46–7.34 (m, 4H), 4.56 (d, *J* = 6.0 Hz, 2H), 4.39–4.18 (m, 4H), 4.11–3.83 (m, 4H) ppm. HRMS (APCI, positive): calcd, 532.1797; *m*/*z*, 533.1873 [M + H]^+^.

#### 3′,5′-(Dichloro-[1,1′-biphenyl]-3,5-diyl)­dimethanol (**7**)

Compound **3** (1.37 g, 4.42 mmol, 1.0 equiv) was suspended in 20 mL of anhydrous THF under argon atmosphere and cooled to 0 °C. The mixture was stirred and treated dropwise with 1 M BH_3_-THF solution in THF (17.70 mL, 17.70 mmol, 4.0 equiv) over 10 min. The ice bath was removed, the mixture was stirred overnight at room temperature and subsequently carefully treated with water leading to the formation of a solid precipitate. The suspension was filtered and the precipitate was washed with water. The remaining solid was dissolved in EtOAc and dried with MgSO_4_, followed by evaporation of the solvent in vacuo. Yield: 1.11 g (3.93 mmol, 89%) of product **7** as a colorless solid. HPLC: 22.5 min, start at 30% B (purity 97.3%). ^1^H NMR (500 MHz, DMSO-*d*_6_): δ = 7.69 (d, *J* = 1.9 Hz, 2H), 7.59 (t, *J* = 1.9 Hz, 1H), 7.55–7.48 (m, 2H), 7.40–7.34 (m, 1H), 5.22 (t, *J* = 5.8 Hz, 2H), 4.57 (d, *J* = 5.7 Hz, 4H) ppm. ^13^C NMR (126 MHz, DMSO-*d*_6_): δ = 143.9, 136.6, 134.6, 126.7, 125.2, 124.9, 123.3, 62.7 ppm. HRMS (EI, positive): calcd, 282.0214; *m*/*z*, 282.0227 [M]^+•^.

#### 3,5-Bis­(bromomethyl)-3′,5′-dichloro-1,1′-biphenyl (**8**)

Compound **7** (1.11 g, 3.93 mmol, 1.0 equiv) was suspended in 55 mL of toluene and heated to 80 °C. PBr_3_ (710 μL, 7.48 mmol, 1.9 equiv) was dissolved in 15 mL of toluene, added dropwise over a period of 10 min and stirred for 1 h. The warm solution was poured into ice water and extracted three times with diethyl ether. The combined organic phases were washed once with brine and dried with MgSO_4_. The solvent was removed in vacuo. Yield: 1.47 g (3.60 mmol, 92%) of compound **8** as a colorless solid. HPLC: 54.5 min, start at 30% B (purity 91.5%). ^1^H NMR (500 MHz, DMSO-*d*_6_): δ = 7.83 (d, *J* = 1.6 Hz, 2H), 7.77 (d, *J* = 1.9 Hz, 2H), 7.64 (t, *J* = 1.9 Hz, 1H), 7.62–7.58 (m, 1H), 4.76 (s, 4H) ppm. ^13^C NMR (126 MHz, DMSO-*d*_6_): δ = 142.3, 139.5, 137.7, 134.7, 130.2, 127.7, 127.3, 125.3, 33.4 ppm. HRMS (EI, positive): calcd, 405.8526; *m*/*z* 405.8518 [M]^+•^.

#### *N*-((1-((5-([4,4′-Bipiperidin]-1-ylmethyl)-3′,5′-dichloro-[1,1′-biphenyl]-3-yl)­methyl)­piperidin-4-yl)­methyl)­acetamide 3 × TFA (**9**)

Compound **8** (1.112 g, 2.72 mmol, 1.0 equiv) was dissolved with *N*-(piperidin-4-ylmethyl)­acetamide × HCl (524 mg, 2.72 mmol, 1.0 equiv) and 1-Boc-Bipiperidine (730 mg, 2.72 mmol, 1.0 equiv) in 70 mL acetonitrile, treated with DIPEA (1.4 mL, 8.16 mmol, 3.0 equiv) and stirred for 2 h at 70 °C. The solvent was removed in vacuo. Ten mL of TFA were added to the residue, the mixture was stirred for 1 h at RT, and the remaining solution was precipitated in diethyl ether. The crude product was purified by preparative HPLC and lyophilized. Yield: 500 mg (0.547 mmol, 20%) of compound **9** as a colorless solid. HPLC: 24.27 min, start at 10% B (purity >99%). ^1^H NMR (500 MHz, DMSO-*d*_6_): δ = 10.16 (br s, 1H), 9.94 (br s, 1H), 8.63 (d, *J* = 11.1 Hz, 1H), 8.45–8.29 (m, 1H), 8.07–7.94 (m, 2H), 7.93 (t, *J* = 6.0 Hz, 1H), 7.78 (d, *J* = 1.9 Hz, 2H), 7.70 (t, *J* = 1.8 Hz, 1H), 7.63–7.58 (m, 1H), 4.35 (s, 4H), 3.44 (t, *J* = 11.5 Hz, 4H), 3.33–3.10 (m, 3H), 3.01–2.88 (m, 5H), 2.88–2.73 (m, 2H), 1.92–1.74 (m, 9H), 1.71–1.60 (m, 1H), 1.51–1.20 (m, 8H) ppm. ^13^C NMR (126 MHz, DMSO-*d*_6_): δ = 169.2, 142.1, 137.9, 134.9, 134.2, 131.1, 131.1, 130.9, 127.6, 125.4, 58.8, 58.8, 51.7, 51.6, 43.3, 43.2, 37.2, 37.1, 33.5, 26.7, 25.8, 25.4, 22.5 ppm and signals of TFA: 158.4 (q, *J* = 34.2 Hz), 116.3 (q, *J* = 294.8 Hz) ppm. HRMS (ESI, positive): calcd, 570.2892, *m*/*z*, 571.2964 [M + H]^+^.

#### *N*,*N*′-((((3′,5′-Dichloro-[1,1′-biphenyl]-3,5-diyl)­bis­(methylene))­bis­(piperidine-1,4-diyl))­bis­(methylene))­diacetamide × 2 TFA (**10**)

Compound **10** was isolated as additional product during the synthesis and preparative HPLC purification of compound **9**. Yield: 212 mg (0.269 mmol, 10%) of compound **10** as a colorless solid. HPLC: 15.05 min, start at 20% B (purity >99%). ^1^H NMR (500 MHz, DMSO-*d*_6_): δ = 10.14–9.68 (m, 2H), 8.04–7.99 (m, 2H), 7.92 (t, *J* = 6.0 Hz, 2H), 7.79 (d, *J* = 1.8 Hz, 2H), 7.70 (t, *J* = 1.8 Hz, 1H), 7.58 (s, 1H), 4.34 (s, 4H), 3.43 (d, *J* = 13.0 Hz, 3.5H), 3.32–3.08 (m, 2H), 3.02–2.83 (m, 6.5H), 1.89–1.71 (m, 10H), 1.72–1.57 (m, 2H), 1.46–1.28 (m, 4H) ppm. ^13^C NMR (126 MHz, DMSO-*d*_6_): δ = 169.2, 142.1, 137.9, 134.9, 134.2, 131.1, 130.9, 127.6, 125.4, 58.8, 51.6, 43.2, 40.0, 33.5, 26.7, 22.5 ppm and signals of TFA: 158.3 (q, *J* = 34.1 Hz), 117.5, 115.2 ppm. HRMS (ESI, positive): calcd, 558.2528; *m*/*z*, 559.2590 [M + H]^+^.

#### 1,1’’-((3′,5′-Dichloro-[1,1′-biphenyl]-3,5-diyl)­bis­(methylene))­di-4,4′-bipiperidine × 4 TFA (**11**)

Compound **11** was isolated as additional product during the synthesis and preparative HPLC purification of compound **9**. Yield: 511 mg (0.492 mmol, 18% yield) of compound **11** as a colorless solid. HPLC: 23.22 min, start at 10% B (purity >99%). ^1^H NMR (500 MHz, DMSO-*d*_6_): δ = 10.20 (br s, 2H), 8.63 (d, *J* = 11.3 Hz, 2H), 8.49–8.19 (m, 2H), 8.01 (d, *J* = 1.1 Hz, 2H), 7.78 (d, *J* = 1.8 Hz, 2H), 7.70 (t, *J* = 1.8 Hz, 1H), 7.64–7.59 (m, 1H), 4.35 (s, 4H), 3.45 (d, *J* = 11.7 Hz, 4H), 3.28 (d, *J* = 12.6 Hz, 4H), 3.00–2.87 (m, 4H), 2.88–2.69 (m, 4H), 2.04–1.66 (m, 8H), 1.56–1.21 (m, 12H) ppm. ^13^C NMR (126 MHz, DMSO-*d*_6_): δ = 142.1, 137.9, 134.9, 134.3, 131.1, 130.9, 127.6, 125.4, 58.8, 51.7, 43.3, 37.2, 37.1, 25.7, 25.3 ppm and signals of TFA: 158.3 (q, *J* = 33.4 Hz), 116.5 (q, *J* = 296.0 Hz) ppm. HRMS (ESI, positive): calcd, 582.3256; *m*/*z*, 583.3339 [M + H]^+^.

#### *N*-((1-((3′,5′-Dichloro-5-((4-(pyridin-4-yl)­piperazin-1-yl)­methyl)-[1,1′-biphenyl]-3-yl)­methyl)­piperidin-4-yl)­methyl)­acetamide × 3 TFA (**27**)

Compound **8** (100 mg, 0.245 mmol, 1 equiv) was heated under stirring with 1-(pyridin-4-yl)­piperazine (40 mg, 0.245 mmol, 1 equiv), *N*-(piperidin-4-ylmethyl)­acetamide × TFA (66 mg, 0.245 mmol, 1 equiv), and NEt_3_ (68 μL, 0.489 mmol, 2 equiv) in 5 mL acetonitrile for 1 h at 70 °C. After cooling to room temperature, the solution was stirred overnight. The solvent was removed in vacuo, the product purified by preparative HPLC, and lyophilized. Yield: 32 mg (0.031 mmol, 13%) of compound **27** as a white solid. HPLC: 13.10 min, start at 20% B (purity >99%). ^1^H NMR (500 MHz, DMSO-*d*_6_): δ = 10.25–9.80 (m, 1H), 8.37 (d, *J* = 7.6 Hz, 2H), 8.00 (s, 2H), 7.94 (t, *J* = 5.8 Hz, 1H), 7.78 (d, *J* = 1.9 Hz, 2H), 7.70 (t, *J* = 1.8 Hz, 1H), 7.59 (s, 1H), 7.27 (d, *J* = 7.7 Hz, 2H), 4.52–4.18 (m, 4H), 3.95 (br s, 4H), 3.51–3.08 (m, 6H), 3.04–2.76 (m, 4H), 1.93–1.73 (m, 5H), 1.72–1.55 (m, 1H), 1.45–1.29 (m, 2H) ppm. ^13^C NMR (126 MHz, DMSO-*d*_6_) δ = 169.2, 156.7, 142.2, 140.2, 138.0, 134.9, 133.9, 131.2, 130.8, 130.6, 127.6, 125.4, 108.0, 58.8, 58.7, 51.5, 50.1, 43.2, 33.5, 26.7, 22.5 ppm and signals of TFA: 158.4 (q, *J* = 34.5 Hz), 117.4, 115.0 ppm. HRMS (APCI, positive): calcd, 565.2375; *m*/*z*, 566.2438 [M + H]^+^.

#### *N*-((1-((3′,5′-Dichloro-5-((1′-glycyl-[4,4′-bipiperidin]-1-yl)­methyl)-[1,1′-biphenyl]-3-yl)­methyl)­piperidin-4-yl)­methyl)­acetamide × 3 TFA (**41**)

Compound **9** (70.0 mg, 0.077 mmol, 1.0 equiv) was dissolved in 2 mL DMF and treated with Boc-Gly-OH (13.4 mg, 0.077 mmol, 1.0 equiv), HATU (29.1 mg, 0.077 mmol, 1.0 equiv), and DIPEA (78 μL, 0.460 mmol, 6.0 equiv). The solution was stirred overnight at room temperature, the solvent was removed in vacuo, and the remaining oily intermediate was subsequentely treated with 2 mL of TFA and stirred at room temperature for 1 h. The crude product was precipitated in cold diethyl ether and the product was purified by preparative HPLC and lyophilized. Yield: 60.3 mg (0.062 mmol, 81%) of compound **41** as a colorless solid. HPLC: 14.86 min, start at 20% B, (purity >99%). ^1^H NMR (500 MHz, DMSO-*d*_6_): δ = 10.30–9.66 (m, 2H), 8.10–7.96 (m, 5H), 7.92 (t, *J* = 6.0 Hz, 1H), 7.78 (d, *J* = 1.9 Hz, 2H), 7.70 (t, *J* = 1.8 Hz, 1H), 7.59 (s, 1H), 4.50–4.27 (m, 6H), 4.00–3.73 (m, 2H), 3.69 (d, *J* = 13.0 Hz, 1H), 3.44 (t, *J* = 10.9 Hz, 4H), 3.23–3.11 (m, 1H), 3.05–2.84 (m, 6H), 2.65–2.55 (m, 1H), 1.95–1.74 (m, 7H), 1.74–1.59 (m, 3H), 1.53–1.23 (m, 5H), 1.19–1.03 (m, 1H), 1.02–0.88 (m, 1H) ppm. ^13^C NMR (126 MHz, DMSO-*d*_6_): δ = 169.2, 164.0, 142.1, 137.9, 134.9, 134.2, 131.1, 130.9, 127.6, 125.4, 58.8, 58.8, 51.8, 51.6, 44.2, 43.2, 41.7, 40.0, 37.5, 33.5, 28.7, 28.2, 26.7, 26.0, 22.5 ppm and signals of TFA: 158.2 (q, *J* = 34.4 Hz), 117.4, 115.0 ppm. HRMS (APCI, positive): calcd, 627.3107; *m*/*z*, 628.3166 [M + H]^+^.

#### *N*-((1-((3′,5′-Dichloro-5-((1′-(2-methoxyethyl)-[4,4′-bipiperidin]-1-yl)­methyl)-[1,1′-biphenyl]-3-yl)­methyl)­piperidin-4-yl)­methyl)­acetamide × 3 TFA (**42**)

Compound **9** (70.0 mg, 0.077 mmol, 1.0 equiv) was dissolved with 1-bromo-2-methoxyethane (8.6 μL, 0.092 mmol, 1.2 equiv) and K_2_CO_3_ (31.8 mg, 0.230 mmol, 3.0 equiv) in 300 μL DMF. The solution was stirred for 3 h at 80 °C, the product was purified by preparative HPLC and lyophilized. Yield: 54.1 mg (0.056 mmol, 73%) of compound **42** as a colorless solid. HPLC: 15.61 min, start 20% B (purity: >99%). ^1^H NMR (500 MHz, DMSO-*d*_6_): δ = 10.33–9.77 (m, 2H), 9.68–9.23 (m, 1H), 8.00 (s, 2H), 7.93 (t, *J* = 6.2 Hz, 1H), 7.78 (d, *J* = 1.8 Hz, 2H), 7.70 (t, *J* = 1.8 Hz, 1H), 7.59 (s, 1H), 4.35 (s, 4H), 3.69–3.61 (m, 2H), 3.54–3.39 (m, 5H), 3.36–3.08 (m, 7H), 3.03–2.79 (m, 7H), 1.91–1.70 (m, 9H), 1.72–1.59 (m, 1H), 1.50–1.28 (m, 8H) ppm. ^13^C NMR (126 MHz, DMSO-*d*_6_): δ = 169.3, 142.1, 137.9, 134.9, 134.2, 131.1, 131.1, 130.9, 127.6, 125.4, 65.6, 58.8, 58.8, 58.1, 55.3, 52.2, 51.7, 51.6, 43.2, 37.0, 36.9, 33.5, 26.7, 25.8, 22.5 ppm and signals of TFA: 158.3 (q, *J* = 35.2 Hz), 116.0 (q, *J* = 293.9 Hz) ppm. HRMS (APCI, positive): calcd, 628.3311; *m*/*z*, 629.3374 [M + H]^+^.

#### Methyl 3-(1′-((5-((4-(acetamidomethyl)­piperidin-1-yl)­methyl)-3′,5′-dichloro-[1,1′-biphenyl]-3-yl)­methyl)- [4,4′-bipiperidin]-1-yl)­propanoate × 3 TFA (**43**)

Compound **9** (68 mg, 0.074 mmol, 1.0 equiv) was dissolved with methyl 3-bromopropanoate (11 μL, 0.093 mmol, 1.3 equiv) and K_2_CO_3_ (31 mg, 0.225 mmol, 3.0 equiv) in 300 μL DMF. The solution was stirred for 2 h at 80 °C, the product was purified by preparative HPLC and lyophilized. Yield: 53 mg (0.053 mmol, 72%) of compound **43** as a colorless solid. HPLC: 15.21 min, start at 20% B, (purity >99%). ^1^H NMR (500 MHz, DMSO-*d*_6_): δ = 10.33–10.01 (m, 1H), 9.87 (br s, 1H), 9.72–9.37 (m, 1H), 8.00 (s, 2H), 7.92 (t, *J* = 6.0 Hz, 1H), 7.78 (d, *J* = 1.8 Hz, 2H), 7.70 (t, *J* = 1.8 Hz, 1H), 7.59 (s, 1H), 4.35 (s, 4H), 3.65 (s, 3H), 3.54–3.39 (m, 6H), 3.35–3.09 (m, 3H), 3.09–2.69 (m, 9H), 1.90–1.75 (m, 9H), 1.71–1.59 (m, 1H), 1.52–1.24 (m, 8H) ppm. ^13^C NMR (126 MHz, DMSO-*d*_6_): δ = 170.4, 169.2, 142.1, 137.9, 134.9, 134.2, 131.1, 131.1, 130.9, 127.6, 125.4, 58.8, 58.7, 52.0, 51.8, 51.7, 51.6, 51.3, 43.2, 37.0, 36.8, 33.5, 28.3, 26.7, 26.0, 25.8, 22.5 ppm and signals of TFA: 158.3 (q, *J* = 33.7 Hz) ppm. HRMS (APCI, positive): calcd, 656.3260; *m*/*z*, 657.3318 [M + H]^+^.

#### 3-(1′-((5-((4-(Acetamidomethyl)­piperidin-1-yl)­methyl)-3′,5′-dichloro-[1,1′-biphenyl]-3-yl)­methyl)-[4,4′-bipiperidin]-1-yl)­propanoic Acid × 3 TFA (**44**)

Compound **43** (30 mg, 0.030 mmol) was suspended in 2 mL dioxane and 1 mL acetone, treated with 1 mL of 1 M LiOH, and stirred for 3 h at room temperature. After acidification with TFA, the solvent was removed in vacuo. The remaining residue was purified by preparative HPLC and lyophilized. Yield: 22 mg (0.022 mmol, 73% yield) of compound **44** as a colorless, resinous solid. HPLC: 14.01 min, start at 20% B (purity >99%). ^1^H NMR (500 MHz, DMSO-*d*_6_): δ = 12.75 (br s, 1H), 10.31–10.00 (m, 1H), 9.88 (br s, 1H), 9.40 (br s, 1H), 8.00 (s, 2H), 7.92 (t, *J* = 6.1 Hz, 1H), 7.78 (d, *J* = 1.8 Hz, 2H), 7.70 (t, *J* = 1.8 Hz, 1H), 7.59 (s, 1H), 4.35 (s, 4H), 3.07–2.80 (m, 7H), 2.74 (t, *J* = 7.4 Hz, 2H), 1.81 (d, *J* = 17.3 Hz, 9H), 1.72–1.58 (m, 1H), 1.55–1.20 (m, 8H) ppm. The missing signals of aliphtic protons are probably covered by the water signal. ^13^C NMR (126 MHz, DMSO-*d*_6_): δ = 171.5, 169.3, 142.1, 137.9, 134.9, 134.2, 131.1, 130.9, 127.6, 125.4, 58.8, 52.0, 51.7, 51.6, 51.6, 43.2, 37.0, 36.8, 33.5, 28.6, 26.7, 26.0, 25.8, 22.5 ppm and signals of TFA: 158.3 (q, *J* = 32.2 Hz), 118.1, 115.7 ppm. MS (ESI, positive): calcd, 642.31; *m*/*z*, 643.27 [M + H]^+^.

### Kinetic Measurements

Furin measurements were performed as triplicates at room temperature in black 96-well FluoroNunc MaxiSorp plates (Nunc, Langenselbold, Germany) using a Tecan Spark microplate reader (λ_ex_ = 380 nm and λ_em_ = 460 nm, Tecan Group AG, Männedorf, Switzerland). For each well, 2 μL inhibitor solution dissolved in DMSO, 20 μL Phac-Arg-Val-Arg-Arg-AMC^12^ substrate solution dissolved in water (concentration in assay 12.5 μM), and 160 μL buffer (100 mM HEPES pH 7.0 containing 0.2% Triton X-100, 2 mM CaCl_2_, 0.02% NaN_3_, and 1 mg·mL^–1^ bovine BSA) were mixed and the measurement was started by adding 20 μL furin solution (concentration in assay 0.95 nM).^26^ Linear regression of the terminal linear part of the progress curves provided the steady-state rates *v*_s_, used for *K*_i_ calculation with eq 1.

For the slow-binding measurement with inhibitor **27**, the progress curves were fitted to eq 2 providing values for the first-order rate constants *k*_obs_ and *v*_s_ for each curve. Only for this measurement, a twice as high substrate concentration of 25 μM was used to achieve a larger value for the term 1 + *S*/*K*_M_ leading to a slower inhibition with smaller *k*_obs_ values for the initial first-order part of the progress curves (see eq 3). This provides a more pronounced and better visible slow-binding behavior, when a normal reader is used and the first data points obtained approximately 25 s after enzyme addition. Equation 3 was also used to calculate the association rate constant *k*_on_ for inhibitor **27**, and with the known *K*_i_ and *k*_on_ values, the dissociation rate constant *k*_off_ was calculated with eq 4.

1 $$v_{s}=V_{\max}\cdot \left[S\right]/\left[K_{m}\cdot \left(1+\left[I\right]/K_{i}\right)+\left[S\right]\right]$$

2 $$\left[P\right]=v_{S}t+\left(v_{0}-v_{S}\right)\left[1-\exp\left(-k_{obs}t\right)\right]/k_{obs}+d$$

3 $$k_{obs}=k_{on}\left[I\right]/\left(1+\left[S\right]/K_{M}\right)+k_{off}$$

4 $$K_{i}=k_{off}/k_{on}$$

Measurements with the related proprotein convertase PC7 (0.1 nM in assay, available from previous studies^27^) were performed with the same volumes, buffer, and plate reader conditions as described above for furin using 12.5 μM of the substrate H-Arg-Arg-Tle-Lys-Arg-AMC·5 TFA (MI-1823) described previously.^28^ The *v*_s_ values were fitted to eq 1 for calculating *K*_i_ values. PC7 *K*_i_ values >700 nM for weak inhibitors were only determined from preassays using inhibitor concentrations of 100 μM, 10 μM, and 1 μM in the presence of 12.5 μM of the substrate MI-1823.

### X-ray Crystallography

Expression of homogeneously glycosylated furin in stably transfected HEK293S-cells and the purification from conditioned medium was performed as described previously.^22,27,29^ For crystallization, equal volumes (∼10 mg/mL furin in 10 mM HEPES, pH 7.5, 100 mM NaCl, 2 mM CaCl_2_) and crystallization solution (100 mM MES, 200 mM K/NaH_2_PO_4_, pH 5.5–6.0 and 2 M NaCl) were mixed and equilibrated against the reservoir (3.0–3.2 M NaCl) in vapor diffusion experiments at 18–20 °C as described in previous studies.^22^

Crystals were soaked overnight in 1.0 M NaCl, 200 mM MES/NaOH pH 5.5, 1 mM CaCl_2_, 10% (w/v) PEG8000 and 20% (v/v) DMSO supplemented with 5 mM of each inhibitor.^16,27^ Crystals were flash cooled in liquid N_2_.

Diffraction data were collected at the European Synchrotron Radiation Facility (ESRF) beamline ID30A-3.^30,31^ The data were processed using XDS^32^ with XDSAPP (v2.9)^33^ and programs of the CCP4 program suite (v.7.1.001).^34^ Data collection statistics of the structures of furin bound with the inhibitors **13**, **24**, **26**, **27**, **34**, and **41** are shown in Table S1. COOT (v.0.8.8)^35^ was used for model building. Refinement was performed in PHENIX (v1.20.1)^36^ using the PDB-ID 7QY0 ^22^ as initial model. Rfree-reflections (initially generated up to 1.0 Å) were transferred to the data sets prior refinement start. Geometry restraints of the inhibitors were obtained with PHENIX eLBOW^37^ and from the GRADE Web server.^38^ Electron density omit maps were calculated in PHENIX (v1.20.1) after one round of torsion angle simulated annealing refinement with omitted ligands. PyMOL^39^ (v 2.0.7, http://www.pymol.org) was used for molecular graphics and structural alignments. Structure factors and coordinates of the complexes of furin bound with the inhibitors **13**, **24**, **26**, **27**, **34**, and **41** have been deposited as PDB entries 9QWF, 9QWG, 9QWB, 9QWE, 9QWD, 9QWC, respectively (Table S1).

### Determination of Antiviral Activity Against the HPAIV Strain SC35M

The used materials, cells, reagents, media, the infection procedure and inhibition of the multicycle replication by the furin inhibitors, as well as the virus titer titration by plaque formation assay was done in an identical manner, as described previously.^14^ The details are described again in the Supporting Information.

## Supplementary Material

















## Notes

Synthesis of additional precursors and inhibitors; HPLC chromatograms for the synthesis of inhibitor **32**; HPLC chromatograms of all inhibitors; structure determination of furin in complex with inhibitors **13**, **24**, **26**, **27**, **34**, **41**; structure of hydrophobic binding pocket for the dichloro-biphenyl segment; structures of furin in complex with inhibitors **13**, **24**, **26**, **27**, **34**, **41**; determination of antiviral activities against the HPAIV strain SC35M (PDF)
Compound molecular formula strings and properties (CSV)
Inhibitor_13_MI-3102_9QWF_model-review (CIF)
Inhibitor_24_MI-3140_9QWG_model-review (CIF)
Inhibitor_26_MI-2456_9QWB_model-review (CIF)
Inhibitor_27_MI-2471_9QWE_model-review (CIF)
Inhibitor_34_MI-2470_9QWD_model-review (CIF)
Inhibitor_41_MI-2464_9QWC_model-review (CIF)

## Abbreviations

Cav: canavanine
CF: cystic fibrosis
DCM: dichloromethane
DIPEA: *N*,*N*-diisopropylethylamine
DMF: *N*,*N*-dimethylformamide
ESI: electrospray ionization
HATU: [dimethylamino-(3-oxidotriazolo­[4,5-*b*]­pyridin-3-ium-1-yl)­methylidene]-dimethylazanium hexafluorophosphate
MOI: multiplicity of infection
PC: proprotein convertase
Phac: phenylacetyl
TFA: trifluoroacetic acid
Tle: *tert* leucine
