# Supplementary material for: Design, Synthesis, and Characterization of Dichlorobiphenyl-Derived Inhibitors of the Proprotein Convertase Furin
Source: J Med Chem. 2025 Nov 30;68(23):25157–70. doi: 10.1021/acs.jmedchem.5c02157 (PMC12703725; doi:10.1021/acs.jmedchem.5c02157)
Supplement: Supplementary file 1 [file jm5c02157_si_001.pdf]

# Supporting Information

**Design, synthesis, and characterization of dichlorobiphenyl-derived inhibitors of the proprotein convertase furin**

Roman W. Lange[1#], Charlotte Boller[1#], Michael Loresch[1], Konstantin Bloch[2], Eva Böttcher-Friebertshäuser[2], Hans Brandstetter[3,4], Sven O. Dahms[3,4], Torsten Steinmetzer[1*]

[1]Institute of Pharmaceutical Chemistry, Philipps University, Marbacher Weg 10, D-35032 Marburg, Germany

[2]Institute of Virology, Philipps University, Hans-Meerwein-Str. 2, 35043 Marburg, Germany

[3]Department of Biosciences and Medical Biology, Structural Biology Lab, Paris Lodron University, Hellbrunnerstraße 34, A-5020 Salzburg, Austria

[4] Center for Tumor Biology and Immunology (CTBI), University of Salzburg, Hellbrunner Straße 34, A-5020 Salzburg, Austria

[#]these authors contributed equally to the work

AUTHOR INFORMATION

*Phone: +49 6421 2825900. E-mail: steinmetzer@uni-marburg.de

**Content**


| | | |
|---|---|---|
| 1. | General information | S3 |
| 2. | Synthesis | S4 |
| 2.1 | Synthesis of precursors | S4 |
| 2.2 | Synthesis of inhibitors | S8 |
| 3. | Synthesis scheme and HPLC chromatograms for the preparation of inhibitor **32** | S28 |
| 4. | HPLC chromatograms of final inhibitors | S30 |
| 5. | Structure determination of furin in complex with inhibitors **13**, **24**, **26**, **27**, **34**, **41** | S51 |
| 6. | Structure of hydrophobic binding pocket for the dichloro-biphenyl segment | S52 |
| 7. | Structures of furin in complex with inhibitors **13**, **24**, **26**, **27**, **34**, **41** | S53 |
| 8. | Determination of antiviral activities against the HPAIV strain SC35M | S54 |
| 9. | References | S56 |

## 1. General information

### Methods

Reagents and solvents were obtained from Alfa Aesar (Kandel, DE), BLDPharm (Kaiserslautern, DE), Carbolution Chemicals (St. Ingbert, DE), Carl Roth (Karlsruhe, DE), Thermo Fisher Scientific (Dreieich, DE), Fluorochem (Frankfurt, DE), Iris Biotech (Marktredwitz, DE), Merck KGaA (Darmstadt, DE), TCI (Eschborn, DE) or VWR international (Darmstadt, DE) and were used without further purification.

Analytical reversed-phase HPLC measurements were performed on a Primaide system (VWR, Hitachi, column: NUCLEODUR C18 ec, 5 µm, 100 Å, 4.6 mm x 250 mm, Macherey-Nagel, Düren , Germany) with 0.1 % TFA in water (solvent A) and 0.1 % TFA in acetonitrile (solvent B) as eluents using a linear gradient with an increase of 1 % solvent B/min at a flow rate of 1 mL/min and detection at 220 nm. Purifications via preparative reversed-phase HPLC were performed on a Knauer Azura system (pump P 2.1 L equipped with pump head E4099AB, detector UVD 2.1L, Knauer GmbH, Berlin, Germany, column: NUCLEODUR C18 ec, 5 µm, 100 Å, 32 mm x 250 mm, Macherey-Nagel, Düren, Germany) using the same solvents as described above for analytical HPLC and a linear gradient with an increase of 0.5 % solvent B/min at a flow rate of 20 mL/min (detection at 220 nm, in few cases with larger amounts at 254 nm). After preparative HPLC, the inhibitors were obtained as lyophilized TFA-salts in a purity > 95 % using a freeze-drier (Martin Christ GmbH, Osterode am Harz, Germany).

Certain inhibitors were converted into their less hygroscopic HCl salts by thrice lyophilization from aqueous 100 mM HCl (about 4-5-fold excess of HCl for each lyophilization step), followed by lyophilization from deionized water.[1]

Some intermediates were analyzed by thin layer chromatography using silica gel 60 F254 TLC plates from (Merck KGaA). Compounds on TLC plates were either detected with UV light (λ = 254 nm) or by spraying with a ninhydrin solution. For column chromatography of few intermediates, silica gel 60 (Merck KGaA: 0.015-0.040 mm) was used.

ESI mass spectra were measured on a QTrap 2000 ESI spectrometer (Applied Biosystems, now part of Thermo Fischer Scientific) and HR-ESI mass spectra were determined using a micrOTOF-Q III spectrometer (Bruker Daltonics, Billerica, MA), whereas HR-APCI mass spectra were measured with a LTQ FT Ultra mass spectrometer (Thermo Fischer Scientific).

NMR-spectra were measured on ECA500 ($^{1}$H: 500 MHz, $^{13}$C: 126 MHz) or ECZ400S ($^{1}$H: 400 MHz, $^{13}$C: 100 MHz) spectrometers (Jeol Germany, Freising, Germany) using the respective deuterated solvents as internal standard.

## 2. Synthesis

Precursors and intermediates, which are only described in the supporting information, are labeled with the suffix s. For intermediates, which have been already described in the main manuscript, the same numbers without suffix as in the main manuscript have been used. Also, for inhibitors the same numbers as in the main manuscript are used in the supporting information.

### 2.1 Synthesis of precursors

*tert-butyl 4-(formamidomethyl)piperidine-1-carboxylate (**1s**).*

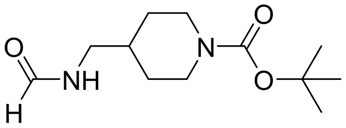

The synthesis was performed according to a literature procedure.[2] To a solution of imidazole (340.4 mg, 5.0 mmol, 2 eq) in 2.5 mL of DMF, *tert*-butyl 4-(aminomethyl)piperidine-1-carboxylate (535 mg, 2.5 mmol, 1 eq) was added and stirred at 140 °C for 24 h. The solvent was removed in vacuo, the remaining residue was dissolved in 5 % aq $KHSO_4$ and extracted three times with EtOAc. The organic layer was 3 × washed with brine and dried over $MgSO_4$. The solvent was removed in vacuo. Yield: 582 mg (2.4 mmol, 96 %) of compound **1s** as a red oil. TLC (DCM/MeOH, 9:1): $R_f$ = 0.375. $^1$H NMR (500 MHz, DMSO-$d_6$): δ = 8.02 (s, 1H), 3.93 (d, 4H), 2.98 (t, 2H), 2.73 (s, 1H), 1.61- 1.53 (m, 2H), 1.39 (s, 9H), 0.99-0.95 (m, 2H) ppm. MS (ESI, positive): calcd, 265.15; *m/z* 265.34 $[M+Na]^+$.

*N-(piperidin-4-ylmethyl)formamide × TFA (**2s**).*

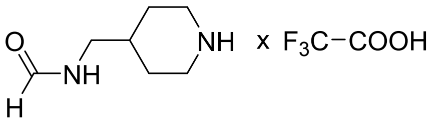

A solution of compound **1s** (580 mg, 2.4 mmol) in 10 mL 17 % TFA in $CH_2Cl_2$ was stirred for 2 h at rt. The solvent was removed in vacuo and the product was precipitated in diethyl ether. Yield: 330 mg (2.32 mmol, 96.7 %) of compound **2s** as an orange oil. TLC (n-Butanol/$H_2O$/acetic acid, 4:1:1): $R_f$ = 0.175. $^1$H NMR (500 MHz, DMSO-$d_6$): δ = 8.04 (s, 1H), 3.25 (d, 4H), 3.02 (t, 2H), 2.84 (d, 4H), 1.77-1.74 (d, 2H), 1.68 (m, 1H, m, 2H), 1.31-1.28 (m, 2H). MS (ESI, positive): calcd, 142.11; *m/z* 143.16 $[M+H]^+$.

*2,2,2-trifluoro-N-(piperidin-4-ylmethyl)acetamide × TFA (**3s**).*

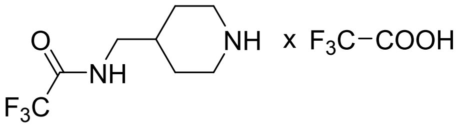

To a solution of 4-(aminomethyl)-piperidin (2.00 g, 17.51 mmol, 1 eq) in 15 mL DCM was added dropwise ethyl trifluoroacetate (2.3 mL, 19.27 mmol, 1.1 eq) dissolved in 25 mL DCM at 0 °C. The mixture was stirred at 0 °C for 2 h and at room temperature for 1.5 h. The solvent was removed in vacuo and the residue purified by preparative HPLC. Yield: 4.59 g (14.15 mmol, 80.8 %) of compound 3s as a colorless crystalline solid. HPLC: 13.3 min, start at 1 % B (purity 96 % ). $^1$H-NMR (500 MHz, DMSO-$d_6$): δ = 9.53 (t, J = 5.6 Hz, 1H), 8.71 (s, 1H), 8.37 (s, 1H), 3.27 (d, J = 12.8 Hz, 2H), 3.11 (t, J = 6.4 Hz, 2H), 2.92 – 2.77 (m, 2H), 1.88 – 1.68 (m, 3H), 1.35 – 1.25 (m, 2H) ppm. $^{13}$C-NMR (126 MHz, DMSO-$d_6$): δ = 158.6, 158.4, 158.1, 157.9, 157.0, 156.7, 156.4, 156.1, 120.3, 119.4, 117.9, 117.1, 115.6, 113.2, 112.5, 43.8, 42.7, 32.9, 26.0. ppm. MS (ESI, positive): calcd, 210.10, *m/z* 211.01 $[M+H]^+$.

*1-methyl-3-(piperidin-4-ylmethyl)urea × HCl (**4s**).*

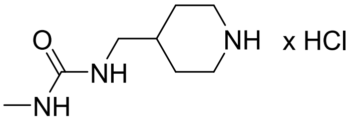

*tert*-butyl 4-(aminomethyl)piperidine-1-carboxylate (500 mg, 2.47 mmol, 1 eq), N-succinimidyl-N-methylcarbamat (638.6 mg, 3.71 mmol, 1.5 eq) and triethylamine (687.5 µL, 4.95 mmol, 2 eq) were dissolved in 30 mL DCM and stirred for 20 h at rt. The solvent was washed three times with 5 % aq $KHSO_4$, once with brine and the solvent was removed in vacuo. The remaining residue was treated with 1 mL 4 N HCl in dioxane and the product was precipitated after 30 min in diethylether. Yield: 405.8 mg (1.95 mmol, 78.9 %) white solid. $^1$H NMR (500 MHz, DMSO-$d_6$): δ = 9.12 (d, J = 8.0 Hz, 1H), 8.80 (d, J = 9.4 Hz, 1H), 4.94 (s, 0H), 3.20 (d, J = 12.7 Hz, 2H), 2.89 (d, J = 6.8 Hz, 2H), 2.82 – 2.64 (m, 2H), 2.53 (s, 3H), 1.73 (d, J = 14.2 Hz, 2H), 1.68 – 1.49 (m, 1H), 1.39 – 1.20 (m, 2H) ppm. $^{13}$C NMR (126 MHz, DMSO-$d_6$): δ = 158.9, 44.2, 42.7, 39.5, 34.3, 26.4, 26.1 ppm. MS (ESI, positive): calcd, 171.14; *m/z* 172.21 $[M+H]^+$.

*Methyl (piperidin-4-ylmethyl)carbamate × HCl (**5s**).*

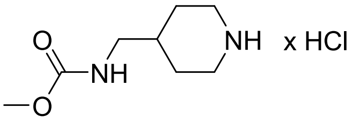


To a solution of 4-aminomethyl-1-Boc-piperidine (500 mg, 2.33 mmol, 1 eq) and DIPEA (812 µL, 4.67 mmol, 2 eq) in DCM, methyl chloroformate (198 µL, 2.57 mmol, 1.1 eq) was added at 0 °C. The solution was stirred for 30 min and then diluted with 25 ml DCM. The organic layer was washed three times each with 5 % aq $KHSO_4$, saturated aq $NaHCO_3$ and brine. The organic layer was dried over $MgSO_4$ and the solvent was removed in vacuo. The remaining oil was treated with 4 mL 4 N HCl in dioxane for 1 hour at room temperature followed by adding diethyl ether providing a viscous oily product. The solvent was decanted off and the remaining residue dried in a vacuum. Yield: 312 mg (1.5 mmol, 64.3 %) of compound **5s** as a yellow oil. $^1$H NMR (500 MHz, DMSO-*d$_6$*): δ = 9.14 (s, 1H), 8.86 (s, 1H), 7.33 – 7.11 (m, 1H), 3.51 (s, 3H), 3.20 (d, J = 12.8 Hz, 2H), 2.87 (t, J = 6.3 Hz, 2H), 2.82 – 2.69 (m, 2H), 1.78 – 1.67 (m, 2H), 1.69 – 1.58 (m, 1H), 1.38 – 1.23 (m, 2H) ppm. MS (ESI, positive): calcd, 172.12, *m/z* 173.15 $[M+H]^+$.

*2,2,2-trifluoro-N-(2-(piperidin-4-yl)ethyl)acetamide × TFA (**6s**).*

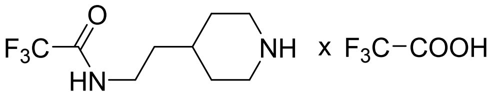


To a solution of 1 eq 4-(aminoethyl)-piperidin (1.00 g, 7.8 mmol) in 10 mL DCM was added dropwise 1.1 eq ethyl trifluoroacetate (1.02 mL, 8.58 mmol) dissolved in 5 mL DCM at 0 °C. The reaction mixture was stirred at 0 °C for 1 h and at room temperature for 3 h. The solvent was removed in vacuo and the remaining residue was purified by preparative HPLC. Yield: 0.456 g (1.35 mmol, 17.3 %) of compound **6s** as a crystalline solid. HPLC: 18.1 min, start at 1 % B (purity 95 %). $^1$H NMR (500 MHz, DMSO-*d$_6$*): δ = 9.42 (t, J = 5.1 Hz, 1H), 8.56 (s, 1H), 8.26 (s, 1H), 3.28 – 3.17 (m, 4H), 2.87 – 2.78 (m, 2H), 1.84 – 1.79 (m, 2H), 1.58 – 1.50 (m, 1H), 1.48 – 1.40 (m, 2H), 1.31 – 1.21 (m, 2H) ppm. MS (ESI, positive): calcd, 224.11, *m/z* 225.04 $[M+H]^+$.

*(5-bromo-1,3-phenylene)dimethanol (**7s**).*

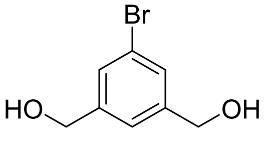


A solution of 5-bromoisophthalic acid (**1**) (1000 mg, 4.08 mmol, 1.0 equiv) in 20 mL of dry THF under argon atmosphere was cooled to 0 °C. Subsequently, a 1 M $BH_3$-THF solution in THF (16.50 mL, 16.50

mmol, 4.0 equiv) was added dropwise over 20 min at 0 °C and the mixture was stirred overnight at room temperature. A few mL of water were carefully added and stirred until effervescence ceased. The solvents were removed in vacuo and the crude product was purified by column chromatography (cyclohexane/ethyl acetate (1:1, v/v), $R_f$ = 0.24). Yield: 695 mg (3.20 mmol, 78 %) of product **X1** as a colorless solid. HPLC: 28 min, start at 1 % B (purity: 98.7 %). $^1$H-NMR (500 MHz, DMSO-$d_6$): δ = 7.37 – 7.32 (m, 2H), 7.26 – 7.23 (m, 1H), 5.34 – 5.20 (m, 2H), 4.50 – 4.47 (m, 4H) ppm. $^{13}$C-NMR (126 MHz, DMSO-$d_6$): δ = 145.1, 127.1, 123.1, 121.2, 62.1 ppm. MS (ESI, positive): calcd, 215.98; m/z, 239.08 $[M+Na]^+$.

*(5-bromo-1,3-phenylene)dimethanamine × 2 TFA (**8s**).*

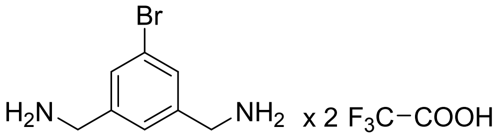


Compound **7s** (100 mg, 0.46 mmol, 1.0 equiv) was dissolved in 5 mL of ethyl acetate, treated with $NEt_3$ (256 µL, 1.84 mmol, 4.0 equiv), and cooled to -5 °C. Mesyl chloride (85.6 µL, 1.11 mmol, 2.4 equiv) was added dropwise. Afterwards, the solution was stirred for 10 min and then warmed to room temperature. 5 mL of DMF and $NaN_3$ (138 mg, 2.12 mmol, 4.6 equiv) were added and the mixture was stirred for 24 h. After dilution with water, the phases were separated. The aqueous phase was extracted three times with ethyl acetate. The combined organic phases were washed three times with water, once with saturated NaCl solution and were dried with $MgSO_4$. The organic phase was concentrated in vacuo to approximately 10 mL. $PPh_3$ (532 mg, 2.03 mmol, 4.4 equiv) was added and the solution was heated to 70 °C for 3.5 h. After cooling to room temperature, 20 mL of water/THF (1:1, v/v) were added and the mixture was stirred vigorously under phase mixing overnight. Subsequently, the phases were separated. The aqueous phase was acidified with 5 % $KHSO_4$ and washed three times with ethyl acetate. The organic phase was discarded. The solvent of the water phase was removed in vacuo and the product purified by preparative HPLC and lyophilized. Yield: 115 mg (0.26 mmol, 57 %) of compound **8s** obtained as a white solid. HPLC: 10.9 min, start at 1 % B (purity: > 99 %). $^1$H-NMR (500 MHz, DMSO-$d_6$): δ = 8.41 (br s, 6H), 7.75 (s, 2H), 7.52 (s, 1H), 4.05 (s, 4H) ppm. $^{13}$C-NMR (126 MHz, DMSO-$d_6$): δ = 136.7, 131.4, 128.7, 121.7, 41.4 ppm and signals of TFA: 158.0 (q, J =30.7 Hz), 118.4, 116.0 ppm. MS (ESI, positive): calcd, 214.01; m/z, 215.07 $[M+H]^+$.

## 2.2 Synthesis of inhibitors

The synthesis of inhibitors **4**-**6** and **9**-**11** have been described in the main manuscript.

The synthesis of inhibitors **12**-**38** is shown in Scheme S1 and described below.

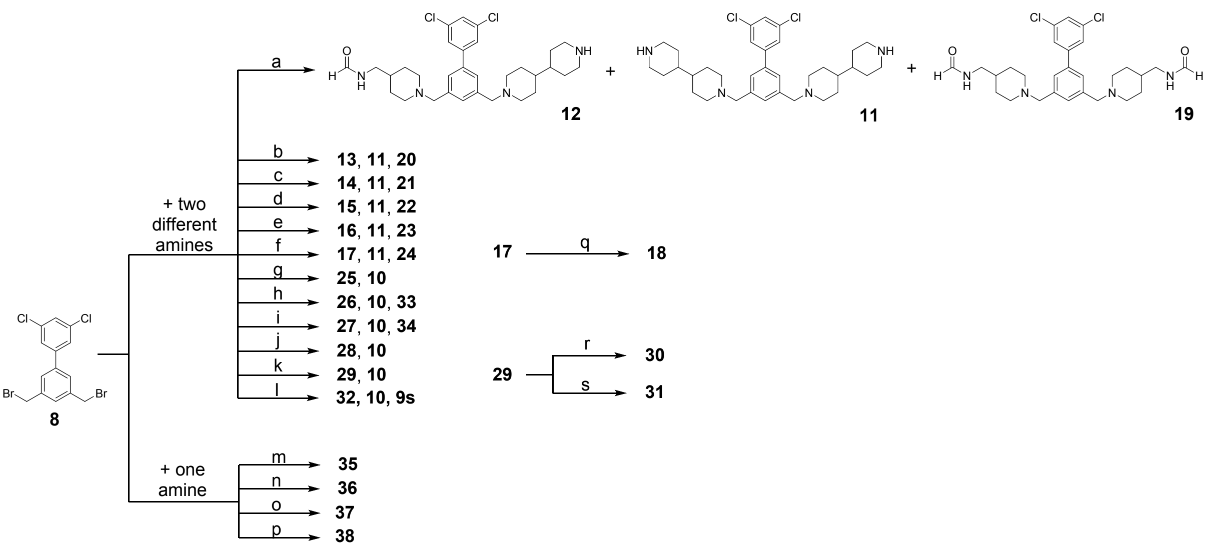


**Scheme S1.** Synthesis of the asymmetric inhibitors **12**-**18** and **25**-**32** by alkylating two different amines by intermediate **8** providing additional symmetric derivatives (**10**-**11**, **19-24**, **33-38**). The following amines have been used for the reaction with dibromide **8**: (a) *tert*-butyl-4,4′-bipiperidine-1-carboxylate, N-(piperidin-4-ylmethyl)formamide × TFA (**2s**); (b) *tert*-butyl-4,4′-bipiperidine-1-carboxylate, 2,2,2-trifluoro-N-(piperidin-4-ylmethyl)acetamide × TFA (**3s**); (c) *tert*-butyl-4,4′-bipiperidine-1-carboxylate, 1-methyl-3-(piperidin-4-ylmethyl)urea × HCl (**4s**); (d) *tert*-butyl-4,4′-bipiperidine-1-carboxylate, methyl (piperidin-4-ylmethyl)carbamate × HCl (**5s**); (e) *tert*-butyl-4,4′-bipiperidine-1-carboxylate, 2,2,2-trifluoro-N-(2-(piperidin-4-yl)ethyl)acetamide × TFA (**6s**); (f) *tert*-butyl-4,4′-bipiperidine-1-carboxylate, methyl 2-(piperidin-4-yl)acetate hydrochloride; (g) 1-methyl-4-(piperidin-4-yl)piperazine, *N*-(piperidin-4-ylmethyl)acetamide × TFA; (h) 1-(1-methylpiperidin-4-yl)piperazine, *N*-(piperidin-4-ylmethyl)acetamide × TFA; (i) 1-(pyridin-4-yl)piperazine, N-(piperidin-4-ylmethyl)acetamide × TFA; (j) 4-phenylpiperidine, *N*-(piperidin-4-ylmethyl)acetamide × HCl; (k) 4-(Boc-amino)-piperidine, *N*-(piperidin-4-ylmethyl)acetamide × HCl; (l) *tert*-butyl (piperidin-4-ylmethyl)carb-amate, *N*-(piperidin-4-ylmethyl)acetamide × HCl. For the synthesis of the symmetric inhibitors **35**-**38**, intermediate **8** was used to alkylate (m) 1-Boc-piperazine, (n) 4-(*N*-Boc-amino)piperidine, (o) piperidine, and (p) pyrrolidine. A subsequent Boc deprotection was done in cases of (a), (b), (c), (d), (e), (f), (k), (l), (m), and (n). (q) saponification of **17** with LiOH; r) HATU-coupling of **29** with Boc-Gly-OH, and subsequent Boc deprotection with TFA; (s) HATU-coupling of **29** with Boc-β-Ala-OH, and

subsequent Boc deprotection with TFA. The second symmetric products during the synthesis of the asymmetric inhibitors **25**, **28**, and **29** could not be isolated.

*N-((1-((5-([4,4'-bipiperidin]-1-ylmethyl)-3',5'-dichloro-[1,1'-biphenyl]-3-yl)methyl)piperidin-4-yl)methyl)formamide × 3 TFA (**12**).*

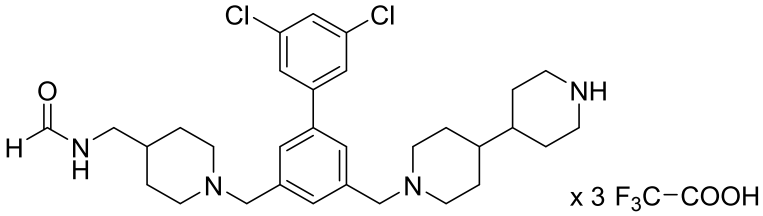


A solution of compound **8** (100 mg, 0.24 mmol, 1 eq), *tert*-butyl-4,4′-bipiperidine-1-carboxylate (65.6 mg, 0.24 mmol, 1 eq), and compound **2s** (62.7 mg, 0.24 mmol, 1 eq) in 5 mL MeCN was treated with DIPEA (128 µL, 0.73 mmol, 3 eq). The solution was stirred at 75 °C for 3 h. The solvent was removed in vacuo and the Boc-protected intermediate purified by preparative HPLC (anal. HPLC: 28.9 min, start at 20 % B, MS (ESI, positive): calcd, 656.33; *m/z* 657.29 $[M+H]^+$). The solvent of the product containing fractions was removed in vacuo and the remaining residue was treated with 2 mL 17 % TFA in DCM and stirred for 1 h. The product was precipitated in diethyl ether, isolated by centrifugation, and dried in vacuo. Yield: 10.34 mg (0.012 mmol, 5 %) of compound **12** as a white lyophilised solid. HPLC: 23.92 min, start at 10 % B (purity 98.2 %). $^1$H-NMR (500 MHz, DMSO-$d_6$): δ = 9.81 (s, 1H), 8.12 (m, 1H), 8.04 (s, 1H), , 8.00 (s, 1H), 7.78 (s, 2H), 7.70 (s, 1H), 7.57 (s, 1H), 4.35 (m, 4H), 3.42 (d, 2H), 3.20 (m, 1H), 3.01- 2.95 (m, 4H), 1.85-1.82 (m, 2H), 1.69-1.64 (m, 1H), 1.41- 1.32 (m, 4H) ppm. MS (ESI, positive): calcd, 556.27; *m/z* 557.21 $[M+H]^+$.

*N-((1-((5-([4,4'-bipiperidin]-1-ylmethyl)-3',5'-dichloro-[1,1'-biphenyl]-3-yl)methyl)piperidin-4-yl)methyl)-2,2,2-trifluoroacetamide × 3 TFA (**13**).*

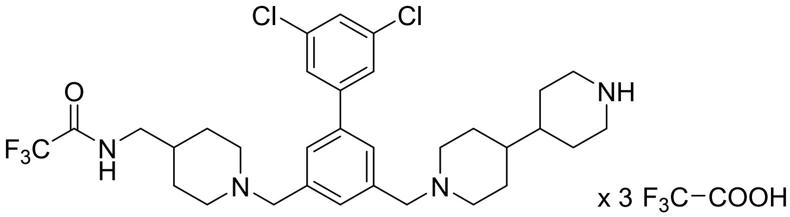


Compound **8** (105 mg, 0.26 mmol, 1 eq), compound **3s** (83.3 mg, 0.26mmol, 1 eq), *tert*-butyl [4,4'-bipiperidine]-1-carboxylate (68.9 mg, 0.26 mmol, 1 eq) and DIPEA (142.3 µL, 1.03 mmol, 4 eq) were dissolved in 20 mL MeCN. The solution was stirred at 75 °C for 6 h. The solvent was removed in vacuo and the remaining residue treated with 2 mL TFA and stirred for 1 h at room temperature. The product was precipitated by adding diethyl ether, isolated by centrifugation and purified by preparative HPLC. Yield: 78.1 mg (0.081 mmol, 31.2 %) of compound **13** as a white lyophilized solid. HPLC: 28.90 min,

start at 10 % B (purity 98.9 %). $^{1}$H NMR (500 MHz, DMSO-$d_6$): δ = 10.20 (s, 1H), 10.05 (s, 1H), 9.54 (t, $J$ = 5.8 Hz, 1H), 8.69 – 8.58 (m, 1H), 8.46 – 8.28 (m, 1H), 8.00 (d, $J$ = 5.4 Hz, 2H), 7.78 (d, $J$ = 1.8 Hz, 2H), 7.70 (t, $J$ = 1.8 Hz, 1H), 7.60 (s, 1H), 4.36 (s, 4H), 3.45 (d, $J$ = 13.2 Hz, 3H), 3.28 (d, $J$ = 14.6 Hz, 3H), 3.11 (t, $J$ = 5.9 Hz, 2H), 3.02 – 2.87 (m, 3H), 2.86 – 2.74 (m, 2H), 1.93 – 1.70 (m, 8H), 1.50 – 1.22 (m, 8H) ppm. $^{13}$C NMR (125 MHz, DMSO- $d_6$): δ = 158.8, 158.5, 158.2, 158.0, 156.7, 156.4, 142.1, 137.9, 134.8, 134.2, 131.1, 130.9, 130.9, 127.6, 125.4, 117.7, 117.0, 115.4, 114.7, 58.8, 58.7, 51.7, 51.3, 43.7, 37.2, 37.1, 32.9, 26.5, 25.8, 25.3 ppm. MS (ESI, positive): calcd, 624.26*; m/z* 625.12 [M+H]$^{+}$.

*1-((1-((5-([4,4'-bipiperidin]-1-ylmethyl)-3',5'-dichloro-[1,1'-biphenyl]-3-yl)methyl)piperidin-4-yl)methyl)-3-methylurea × 3 TFA (**14**).*

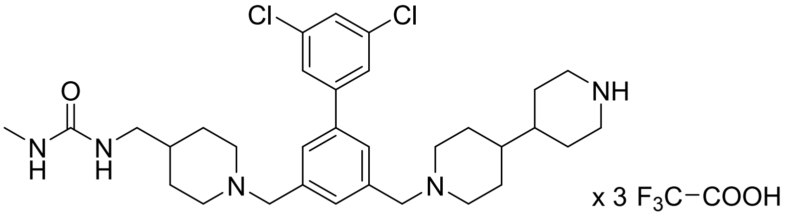


A solution of compound **8** (100 mg, 0.244 mmol, 1 eq), compound **4s** (50.8 mg, 0.244 mmol, 1 eq) and *tert*-butyl-4,4′-bipiperidine-1-carboxylate (65.6 mg, 0.244 mmol, 1 eq) in 15 mL of MeCN was treated with DIPEA (128 µL, 0.73 mmol, 3 eq) and stirred at 75 °C for 6 h. The solvent was removed in vacuo and the Boc-protected intermediate purified by preparative HPLC (anal. HPLC: 19.8 min, start at 30 % B, MS (ESI, positive): calcd, 685.35; *m/z* 686.29 [M+H]$^{+}$). The solvent of the product containing fractions was removed in vacuo. The remaining residue was treated with 2 mL TFA, the mixture was stirred for 1 h. The product was precipitated by adding diethyl ether and isolated by centrifugation and drying in vacuo. Yield: 22.3 mg (0.024 mmol, 9.8 %) of inhibitor **14** as a white lyophilized solid. HPLC: 24.30 min, start at 10 %B (purity > 99 %). $^{1}$H NMR (500 MHz, DMSO-$d_6$): δ = 10.13 (s, 1H), 9.84 (s, 1H), 8.69 – 8.54 (m, 1H), 8.45 – 8.29 (m, 1H), 8.00 (s, 2H), 7.78 (d, J = 1.8 Hz, 2H), 7.70 (t, J = 1.8 Hz, 1H), 7.60 (s, 1H), 6.07 (s, 1H), 4.35 (s, 4H), 3.44 (t, J = 11.4 Hz, 4H), 3.28 (d, J = 13.6 Hz, 2H), 3.21 – 3.09 (m, 1H), 2.98 – 2.90 (m, 3H), 2.88 (d, J = 6.7 Hz, 2H), 2.86 – 2.75 (m, 3H), 2.54 – 2.49 (m, 2H), 1.91 – 1.71 (m, 7H), 1.67 – 1.52 (m, 1H), 1.47 – 1.22 (m, 8H). MS (ESI, positive) calcd, 585.30; *m/z* 586.29 [M+H]$^{+}$.

*Methyl ((1-((5-([4,4'-bipiperidin]-1-ylmethyl)-3',5'-dichloro-[1,1'-biphenyl]-3-yl)methyl)piperidin-4-yl)methyl)carbamate × 3 TFA (**15**).*

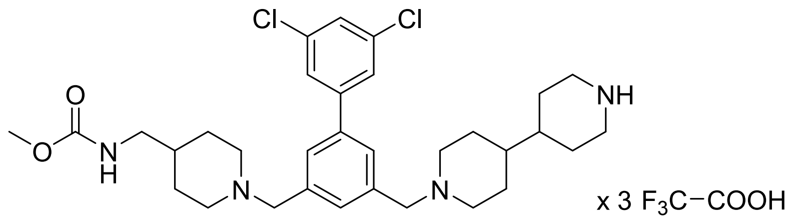


To a solution of compound **8** (100 mg, 0.24 mmol, 1 eq), compound **5s** (51 mg, 0.24 mmol, 1 eq) and *tert*-butyl-4,4′-bipiperidine-1-carboxylate (65 mg, 0.24mmol, 1 eq) in 20 mL MeCN was added DIPEA (128 µL, 0.73 mmol, 3 eq). The solution was stirred at 75 °C for 4 h. The solvent was removed in vacuo, the remaining residue was treated with 2 mL TFA and stirred for 1 h at room temperature. The product was precipitated after in diethyl ether, isolated by centrifugation and purified by preparative HPLC. Yield: 40 mg (0.043 mmol, 18 %) of inhibitor **15** as a white lyophilized solid. HPLC: 25.89 min, start at 10 % B (purity 99 %). $^1$H NMR (500 MHz, DMSO-$d_6$): δ = 10.06 (s, 1H), 9.80 (s, 1H), 8.58 (d, J = 11.6 Hz, 1H), 8.31 (d, J = 11.8 Hz, 1H), 8.11 – 7.90 (m, 2H), 7.78 (d, J = 1.8 Hz, 2H), 7.73 – 7.67 (m, 1H), 7.59 (s, 1H), 7.24 (t, J = 6.3 Hz, 1H), 4.35 (s, 4H), 3.51 (s, 3H), 3.48 – 3.37 (m, 4H), 3.28 (d, J = 14.0 Hz, 2H), 2.99 – 2.86 (m, 5H), 2.87 – 2.76 (m, 2H), 1.92 – 1.73 (m, 7H), 1.72 – 1.55 (m, 1H), 1.47 – 1.21 (m, 8H) ppm. MS (ESI, positive): calcd, 586.28; *m/z* 587.20 $[M+H]^+$.

*N-(2-(1-((5-([4,4'-bipiperidin]-1-ylmethyl)-3',5'-dichloro-[1,1'-biphenyl]-3-yl)methyl)piperidin-4-yl)ethyl)-2,2,2-trifluoroacetamide × 4 TFA (**16**).*

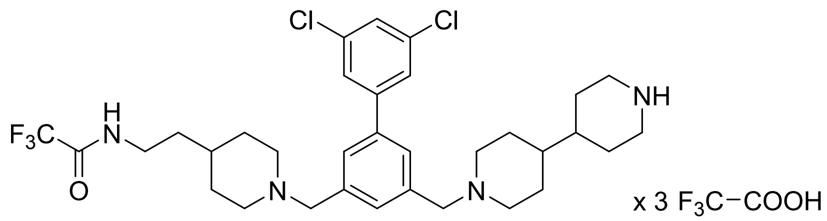


To a solution of compound **8** (50.0 mg, 0.12 mmol, 1 eq), compound **6s** (41.4 mg, 0.12 mmol, 1 eq), and *tert*-butyl-4,4′-bipiperidine-1-carboxylate (32.8 mg, 0.12 mmol, 1 eq) in 20 mL MeCN, DIPEA (85 µL, 4 eq, 0.49 mmol) was added. The solution was stirred at 75 °C for 3.5 h. The solvent was removed in vacuo, the remaining residue treated with 2 mL TFA, and stirred for 1 hour. The product was precipitated by adding diethyl ether and the product purified by preparative HPLC. Yield: 37.7 mg (0.038 mmol, 31.7 %) of compound **16** as a white lyophilized solid. HPLC: 30.07 min, start at 10 % B (purity 99 %). $^1$H-NMR (500 MHz, DMSO-$d_6$): δ = 10.22 (s, 1H), 10.07 (s, 1H), 9.44 (t, J = 5.6 Hz, 1H), 8.65 (d, J = 11.5 Hz, 1H), 8.39 (d, J = 10.4 Hz, 1H), 8.01 (s, 2H), 7.78 (d, J = 1.8 Hz, 2H), 7.69 (t, J = 1.7 Hz, 1H), 7.61 (s, 1H), 4.35 (s, 4H), 3.44 (t, J = 13.8 Hz, 4H), 3.32 – 3.16 (m, 5H), 2.99 – 2.88 (m, 3H), 2.88 – 2.75 (m, 2H), 1.95 – 1.72 (m, 7H), 1.49 – 1.23 (m, 10H) ppm. $^{13}$C-NMR (126 MHz, DMSO-$d_6$): δ = 158.8, 158.5,

158.2, 158.0, 156.3, 156.0, 142.1, 137.9, 134.8, 134.2, 131.1, 130.9, 127.6, 125.4, 117.9, 117.0, 115.6, 114.8, 58.7, 51.7, 43.3, 37.2, 37.1, 36.5, 34.2, 30.4, 28.5, 25.8, 25.3 ppm. MS (ESI, positive): calcd, 638.28; *m/z* 639.30 $[M+H]^+$.

*Methyl 2-(1-((5-([4,4'-bipiperidin]-1-ylmethyl)-3',5'-dichloro-[1,1'-biphenyl]-3-yl)methyl)piperidin-4-yl)acetate × 3 TFA (**17**).*

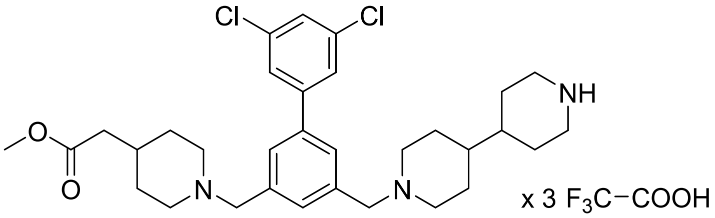


A solution of compound **8** (200 mg, 0.49 mmol, 1 eq), *tert*-butyl [4,4'-bipiperidine]-1-carboxylate (131.3 mg, 0.49 mmol, 1 eq) and methyl 2-(piperidin-4-yl)acetate hydrochloride (94.7 mg, 0.49 mmol, 1 eq) in 20 mL MeCN was treated with DIPEA (255.6 µL, 1.47 mmol, 3 eq). The solution was stirred for 3 h at 75 °C. The solvent was removed in vacuo and the remaining residue was treated with 2 mL TFA. The product was precipitated in diethyl ether and purified by preparative HPLC. Yield: 80.5 mg (0.088 mmol, 17.9 %) of compound **17** as a white lyophilizate. HPLC: 20.73 min, start at 10 %B (purity 100 %). $^1$H NMR (500 MHz, DMSO-$d_6$) δ 10.17 (s, 1H), 9.99 (s, 1H), 8.62 (d, J = 11.0 Hz, 1H), 8.35 (d, J = 11.4 Hz, 1H), 8.01 (s, 2H), 7.79 (d, J = 1.8 Hz, 2H), 7.70 (t, J = 1.8 Hz, 1H), 7.60 (s, 1H), 4.35 (s, 4H), 3.59 (s, 3H), 3.51 – 3.35 (m, 4H), 3.28 (d, J = 13.5 Hz, 2H), 3.08 – 2.88 (m, 4H), 2.87 – 2.75 (m, 2H), 2.29 (d, J = 6.8 Hz, 2H), 2.00 – 1.89 (m, 1H), 1.92 – 1.81 (m, 4H), 1.83 – 1.69 (m, 2H), 1.53 – 1.38 (m, 4H), 1.39 – 1.17 (m, 4H) ppm. $^{13}$C NMR (126 MHz, DMSO-$D_6$) δ 171.8, 158.3 (q, J = 33.4 Hz), 142.1, 137.9, 134.8, 134.3, 131.1, 130.9, 127.6, 125.4, 120.0, 117.7, 115.3, 113.0, 51.7, 51.5, 51.3, 43.3, 39.5, 37.2, 37.1, 30.0, 28.4, 25.8, 25.3 ppm. MS (ESI,positive): calcd, 571.27; *m/z* 572.46 $[M+H]^+$.

*2-(1-((5-([4,4'-bipiperidin]-1-ylmethyl)-3',5'-dichloro-[1,1'-biphenyl]-3-yl)methyl)piperidin-4-yl)acetic acid × 3 TFA (**18**).*

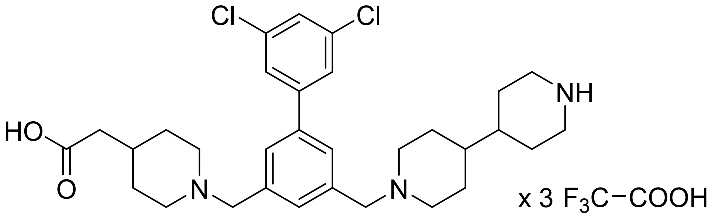


Inhibitor **17** (40 mg, 0.044 mmol, 1 eq) was dissolved in a mixture of 3 mL of 1,4-dioxane and 3 mL 1 N LiOH. The solution was stirred for 3 h at room temperature. After addition of TFA, the solvent was removed in vacuo and the product was purified by preparative HPLC. Yield: 34 mg (0.0378 mmol, 85 %) of compound **18** as a white lyophilized solid. HPLC: 18.00 min, start at 10 %B (purity >99 %). $^1$H NMR

(400 MHz, DMSO-d6) δ 10.16 (s, 1H), 9.96 (s, 1H), 8.64 (d, J = 11.2 Hz, 1H), 8.36 (d, J = 10.2 Hz, 1H), 8.06 – 7.93 (m, 2H), 7.78 (d, J = 1.8 Hz, 2H), 7.70 (t, J = 1.8 Hz, 1H), 7.60 (s, 1H), 4.35 (s, 4H), 3.58 – 3.37 (m, 4H), 3.28 (d, J = 12.6 Hz, 2H), 3.24 – 3.10 (m, 1H), 3.07 – 2.87 (m, 3H), 2.89 – 2.73 (m, 2H), 2.19 (d, J = 6.5 Hz, 2H), 1.87 (t, J = 12.6 Hz, 5H), 1.77 (d, J = 11.7 Hz, 2H), 1.57 – 1.37 (m, 4H), 1.41 – 1.15 (m, 4H) ppm. MS (ESI, positive): calcd, 557.26; *m/z* 558.46.

*N,N'-((((3',5'-dichloro-[1,1'-biphenyl]-3,5-diyl)bis(methylene))bis(piperidine-1,4-diyl))bis(methylene))diformamide × 2 TFA (**19**).*

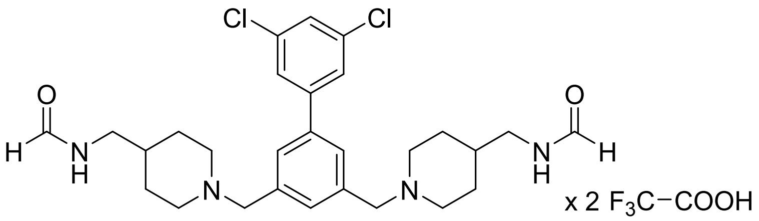


Inhibitor **19** was isolated as a second product during the preparative purification of compound **12**. Yield: 14.3 mg (0.019 mmol, 7.9 %) of compound **19** as a white lyophilized solid. HPLC: 14.75 min, start at 20 %B (purity 99.3 %). $^1$H NMR (500 MHz, DMSO-$d_6$): δ = 9.82 (s, 1H) 9.60 (s, 1H),8.48 (s, 1H), 8.20 (s, 1H), 8.05 (s, 1H), 8.01 (s, 1H), 7.79 (s, 2H), 7.72 (s, 1H), 7.57 (s, 1H), 4.35 (s, 4H), 3.02- 2.75 (m, 8H), 1.85- 1.76 (m, 6H), 1.41-1.24 (m, 8H) ppm. MS (ESI, positive): calcd, 530.22; *m/z* 531.16 $[M+H]^+$.

*N,N'-((((3',5'-dichloro-[1,1'-biphenyl]-3,5-diyl)bis(methylene))bis(piperidine-1,4-diyl))bis(methylene))bis(2,2,2-trifluoroacetamide) × 2 TFA (**20**).*

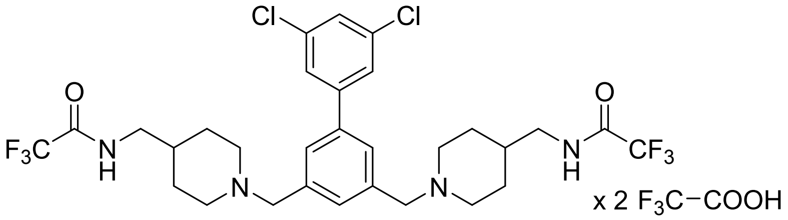


Compound **20** was isolated as a second product during the preparative purification of inhibitor **13**. Yield: 17.8 mg (0.02 mmol, 7.7 %) of compound **20** as a white lyophilized solid. HPLC: 36.89 min, start at 10 % B (purity 97.2 %). $^1$H-NMR (500 MHz, DMSO-$d_6$): δ = 9.68 (s, 2H), 9.51 (t, J = 5.8 Hz, 2H), 8.00 (s, 2H), 7.79 (d, J = 1.8 Hz, 2H), 7.72 (t, J = 1.8 Hz, 1H), 7.55 (s, 1H), 4.34 (s, 4H), 3.44 (d, J = 12.8 Hz, 4H), 3.12 (t, J = 6.3 Hz, 4H), 3.03 – 2.90 (m, 4H), 1.89 – 1.71 (m, 6H), 1.45 – 1.23 (m, 4H) ppm. $^{13}$C-NMR (126 MHz, DMSO-$d_6$): δ = 158.7, 158.5, 158.2, 157.9, 157.0, 156.7, 156.4, 156.1, 142.1, 140.1, 137.9, 134.8, 134.2, 131.1, 130.9, 127.6, 125.4, 119.4, 117.0, 114.7, 58.8, 51.3, 43.7, 32.9, 26.5 ppm. MS (ESI,positive): calcd, 666.20; *m/z* 667.10 $[M+H]^+$.

*1,1'-((((3',5'-dichloro-[1,1'-biphenyl]-3,5-diyl)bis(methylene))bis(piperidine-1,4-diyl))bis(methylene))bis(3-methylurea) × 2 TFA (**21**).*

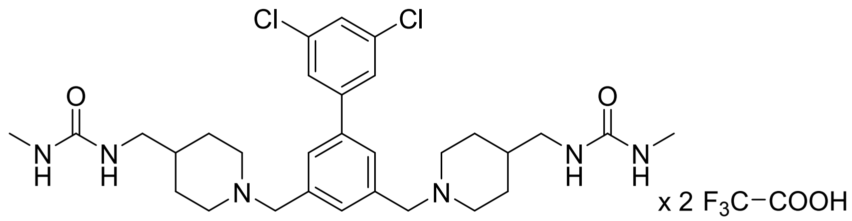


Inhibitor **21** was isolated as a second product during the preparative purification of inhibitor **14**. Yield: 17.3 mg (0.021 mmol, 8.6 %) of compound **21** as a white lyophilized solid. HPLC: 16.06 min, start at 20 % B (purity 100 %). $^{1}$H NMR: (500 MHz, DMSO-d$_6$): δ = 9.70 (s, 2H), 8.04 – 7.94 (m, 2H), 7.79 (d, J = 1.8 Hz, 2H), 7.71 (t, J = 1.8 Hz, 1H), 7.57 (s, 1H), 6.06 (s, 2H), 4.42 – 4.24 (m, 4H), 3.43 (d, J = 12.1 Hz, 4H), 3.26 – 3.12 (m, 1H), 3.12 – 3.03 (m, 1H), 3.03 – 2.81 (m, 8H), 2.52 (s, 4H), 1.86 – 1.73 (m, 4H), 1.68 – 1.54 (m, 2H), 1.44 – 1.19 (m, 4H) ppm. $^{13}$C NMR (126 MHz, DMSO-d$_6$) δ 158.7, 158.4, 158.1, 157.9, 142.1, 137.9, 134.9, 134.1, 131.1, 130.9, 127.6, 125.4, 117.3, 115.0, 58.8, 51.7, 44.1, 40.0, 39.5, 34.2, 26.7, 26.3 ppm.MS (ESI, positive): calcd, 589.28; *m/z* 589.20 [M+H]$^+$.

*Dimethyl ((((3',5'-dichloro-[1,1'-biphenyl]-3,5-diyl)bis(methylene))bis(piperidine-1,4-diyl))bis(methylene))dicarbamate × 2 TFA (**22**).*

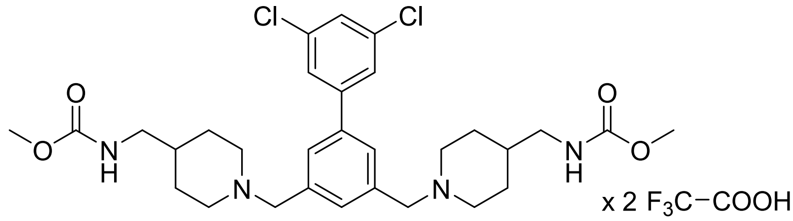


Inhibitor **22** was isolated as a second product during the preparative purification of inhibitor **15**. Yield: 16 mg (0.02mol, 8.3 %) of compound **22** as a white lyophilized solid. HPLC: 30.22 min, start at 10 % B (purity 97.0 %). $^{1}$H NMR (500 MHz, DMSO-*d$_6$*): δ = 9.70 (s, 2H), 8.00 (s, 2H), 7.79 (d, J = 1.8 Hz, 2H), 7.71 (t, J = 1.8 Hz, 1H), 7.56 (s, 1H), 7.31 – 7.16 (m, 2H), 4.34 (s, 4H), 3.70 – 3.30 (m, 6H), 2.95 (d, J = 10.2 Hz, 4H), 2.89 (t, J = 6.3 Hz, 4H), 2.03 – 1.70 (m, 6H), 1.66 (s, 2H), 1.49 – 1.18 (m, 6H) ppm. MS (ESI, positive): calcd, 590.24; *m/z* 591.20 [M+H]$^+$.

*N,N'-((((3',5'-dichloro-[1,1'-biphenyl]-3,5-diyl)bis(methylene))bis(piperidine-1,4-diyl))bis(ethane-2,1-diyl))bis(2,2,2-trifluoroacetamide) × 2 TFA (**23**).*

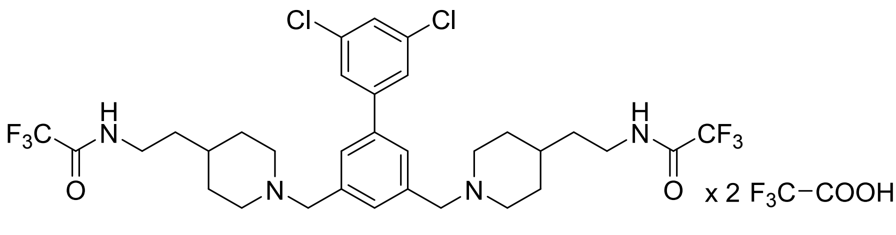

Compound **8** (52.3 mg, 1 eq, 0.13 mmol), compound **6s** (121.8 mg, 0.26 mmol, 2 eq) and DIPEA (90 µL, 0.52 mmol, 4 eq) were dissolved in 20 mL MeCN and the solution was stirred at 75 °C for 2 h. The solvent was removed in vacuo and the product purified by preparative HPLC. Yield: 35.6 mg (0.039 mmol, 30 %) of compound **23** as a white lyophilized solid. HPLC: 28.69 min, start at 20% B (purity > 99 %). $^{1}$H NMR (500 MHz, DMSO-d6): δ = 10.13 – 9.89 (m, 2H), 9.42 (t, J = 5.7 Hz, 2H), 8.01 (s, 2H), 7.79 (d, J = 1.8 Hz, 2H), 7.70 (t, J = 1.8 Hz, 1H), 7.60 (s, 1H), 4.35 (s, 4H), 3.63 – 3.35 (m, 4H), 3.22 (q, J = 6.8 Hz, 4H), 3.01 – 2.88 (m, 4H), 1.89 (d, J = 14.1 Hz, 4H), 1.54 – 1.30 (m, 10H) ppm. $^{13}$C NMR (125 MHz, DMSO-$d_6$): δ = 158.7, 158.4, 158.2, 157.9, 156.6, 156.3, 156.0, 155.7, 142.1, 137.9, 134.9, 134.2, 131.1, 130.9, 127.6, 125.4, 119.3, 117.1, 114.8, 58.8, 51.7, 36.5, 34.2, 30.4, 28.6 ppm. MS (ESI, positive): calcd, 695.23; m/z 695.14 [M+H]$^+$.

*dimethyl 2,2'-(((3',5'-dichloro-[1,1'-biphenyl]-3,5-diyl)bis(methylene))bis(piperidine-1,4-diyl))diacetate × 2 TFA (**24**).*

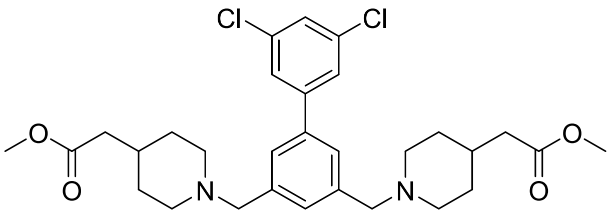


Compound **24** was isolated as a second product during the preparative purification of inhibitor **17**. Yield: 45 mg (0.057 mmol, 11.6 %) of compound **24** as a white lyophilized solid. HPLC: 34.41 min, start at 10 % B (purity 99.1 %). $^{1}$H-NMR (500 MHz, DMSO-$d_6$) δ 9.99 (s, 2H), 8.00 (s, 2H), 7.78 (s, 2H), 7.70 (t, J = 1.7 Hz, 1H), 7.59 (s, 1H), 4.34 (s, 4H), 3.59 (s, 8H), 3.42 (d, J = 12.3 Hz, 5H), 3.00 (s, 3H), 2.30 (d, J = 6.8 Hz, 3H), 2.00 – 1.91 (m, 2H), 1.91 – 1.78 (m, 4H), 1.54 – 1.35 (m, 3H). $^{13}$C NMR (126 MHz, DMSO-$D_6$) δ 171.8, 158.29 (q, *J* = 31.7 Hz), 142.1, 137.9, 134.8, 131.0, 130.9, 127.6, 125.4, 119.4, 118.2, 115.7, 58.8, 51.5, 51.3, 39.5, 31.2, 30.0, 28.4. MS (ESI, positive): calcd, 561.23; *m/z* 561.41 [M+H]$^+$.

*N-((1-((3',5'-dichloro-5-((4-(4-methylpiperazin-1-yl)piperidin-1-yl)methyl)-[1,1'-biphenyl]-3-yl)methyl)piperidin-4-yl)methyl)acetamide × 4 TFA (**25**).*

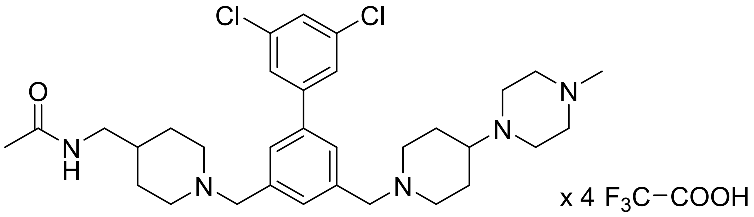

Compound **8** (100 mg, 0.245 mmol, 1 equiv), 1-Methyl-4-(piperidin-4-yl)piperazine (45 mg, 0.245 mmol, 1 equiv), *N*-(piperidin-4-ylmethyl)acetamide × TFA (66 mg, 0.245 mmol, 1 equiv), and $K_2CO_3$ (203 mg, 1.47 mmol, 6 equiv) were dissolved in 2 mL DMF and stirred for 2 h at 80 °C. The solvent was removed in vacuo and the product was purified by preparative HPLC and lyophilized. Yield: 23 mg (0.022 mmol, 9 %) of compound **25** as a colorless, resinous solid. HPLC: 12.57 min, start at 20 % B (purity: > 98.1 %). $^1$H-NMR (500 MHz, DMSO-$d_6$): δ = 10.28 (s, 1H), 10.14 – 9.68 (m, 1H), 8.00 (s, 2H), 7.92 (t, J = 5.9 Hz, 1H), 7.78 (d, J = 1.9 Hz, 2H), 7.70 (t, J = 1.8 Hz, 1H), 7.59 (s, 1H), 4.35 (d, J = 11.8 Hz, 4H), 3.65 – 2.66 (m, 22H), 2.03 (d, J = 13.1 Hz, 2H), 1.89 – 1.56 (m, 8H), 1.43 – 1.23 (m, 2H) ppm. $^{13}$C-NMR (126 MHz, DMSO-$d_6$): δ = 169.2, 142.1, 138.0, 134.9, 134.2, 131.2, 131.0, 127.6, 125.4, 58.8, 58.5, 57.7, 52.3, 51.6, 50.7, 45.5, 43.2, 42.1, 33.5, 26.7, 24.9, 22.5 ppm and signals of TFA: 158.4 (q, J = 34.9 Hz), 116.1 (q, J= 294.3 Hz) ppm. HRMS (APCI, positive): calcd, 585.3001; m/z, 586.3064 $[M+H]^+$.

*N-((1-((3',5'-dichloro-5-((4-(1-methylpiperidin-4-yl)piperazin-1-yl)methyl)-[1,1'-biphenyl]-3-yl)methyl)piperidin-4-yl)methyl)acetamide × 4 TFA (**26**).*

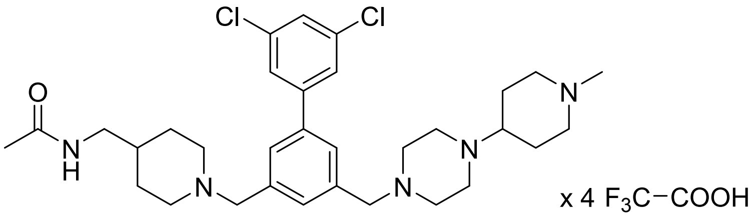


Compound **8** (100 mg, 0.245 mmol, 1.0 equiv) was dissolved with *N*-(piperidin-4-ylmethyl)acetamide × TFA (66 mg, 0.245 mmol, 1.0 equiv) and 1-(1-Methylpiperidin-4-yl)piperazine (45 mg, 0.245 mmol, 1.0 equiv) in 5 mL acetonitrile, treated with $NEt_3$ (68 µL, 0.489 mmol, 2.0 equiv), heated for 1 h at 70 °C and then stirred overnight at room temperature. The solvent was removed in vacuo and the crude product was purified by preparative HPLC and lyophilized. Yield: 16.1 mg (0.015 mmol, 6 %) of compound **26** as a colorless solid. HPLC: 13.15 min, start at 20 % B (purity: > 99 %). $^1$H-NMR (500 MHz, DMSO-$d_6$): δ = 10.25 – 9.78 (m, 2H), 7.99 – 7.84 (m, 3H), 7.77 (d, J = 1.8 Hz, 2H), 7.68 (t, J = 1.8 Hz, 1H), 7.55 (s, 1H), 4.34 (s, 2H), 4.10 (br s, 2H), 3.60 – 3.33 (m, 4H), 3.32 – 2.64 (m, 18H), 2.13 (d, J = 12.7 Hz, 2H), 1.90 – 1.59 (m, 8H), 1.46 – 1.25 (m, 2H) ppm. $^{13}$C-NMR (126 MHz, DMSO-$d_6$): δ = 169.2, 142.4, 137.8, 134.8, 131.0, 127.5, 125.4, 59.1, 58.8, 58.0, 52.2, 51.5, 50.1, 46.7, 43.2, 42.3, 33.5, 26.7, 24.7, 22.5 ppm and signals of TFA: 158.4 (q, J = 34.3 Hz), 117.5, 115.1 ppm. HRMS (APCI, positive): calcd, 585.3001; m/z, 586.3065 $[M+H]^+$.

The synthesis of inhibitor **27** has been described in the main manuscript.

*N-((1-((3',5'-dichloro-5-((4-phenylpiperidin-1-yl)methyl)-[1,1'-biphenyl]-3-yl)methyl)piperidin-4-yl)methyl)acetamide × 2 TFA (**28**).*

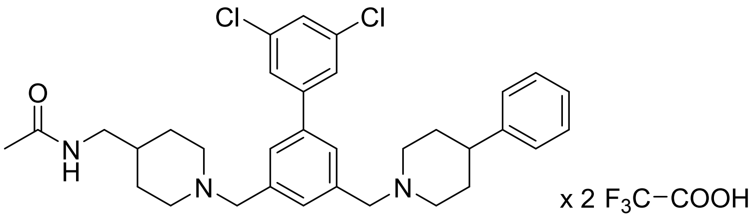


Compound **8** (100 mg, 245 µmol, 1.0 equiv) was dissolved with *N*-(piperidin-4-ylmethyl)acetamide × HCl (47 mg, 245 µmol, 1.0 equiv) and 4-phenylpiperidine (40 mg, 245 µmol, 1.0 equiv) in 5 mL acetonitrile, treated with DIPEA (128 µL, 734 µmol, 3.0 equiv), and stirred for 3 h at 70 °C. The crude product was purified by preparative HPLC and lyophilized. Yield: 46 mg (58 µmol, 24 %) of compound **28** as a colorless solid. HPLC: 27.0 min, start at 20 % B (purity > 99 %). $^{1}$H-NMR (500 MHz, DMSO-$d_6$): δ = 10.29 – 10.00 (m, 1H), 9.97 – 9.63 (m, 1H), 8.08 – 8.05 (m, 1H), 8.04 – 8.00 (m, 1H), 7.91 (t, J = 5.9 Hz, 1H), 7.81 (d, J = 1.9 Hz, 2H), 7.71 (t, J = 1.8 Hz, 1H), 7.65 – 7.60 (m, 1H), 7.40 – 7.15 (m, 5H), 4.43 (s, 2H), 4.36 (d, J = 3.2 Hz, 2H), 3.75 – 3.35 (m, 4H), 3.29 – 3.06 (m, 3H), 3.02 – 2.90 (m, 3H), 2.88 – 2.74 (m, 1H), 2.05 – 1.74 (m, 9H), 1.72 – 1.59 (m, 1H), 1.46 – 1.28 (m, 2H) ppm. $^{13}$C-NMR (126 MHz, DMSO-$d_6$): δ = 169.2, 144.0, 142.1, 138.0, 134.9, 134.2, 131.2, 131.1, 131.0, 128.6, 127.6, 126.6, 126.4, 125.4, 58.9, 58.8, 52.0, 51.6, 43.1, 38.6, 33.5, 29.9, 26.7, 22.5 ppm and signals of TFA: 158.3 (q, J = 35.2 Hz), 117.1, 114.8 ppm. HRMS (APCI, positive): calcd, 563.2470; m/z, 564.2528 $[M+H]^+$.

*N-((1-((5-((4-aminopiperidin-1-yl)methyl)-3',5'-dichloro-[1,1'-biphenyl]-3-yl)methyl)piperidin-4-yl)methyl)acetamide × 3 TFA (**29**).*

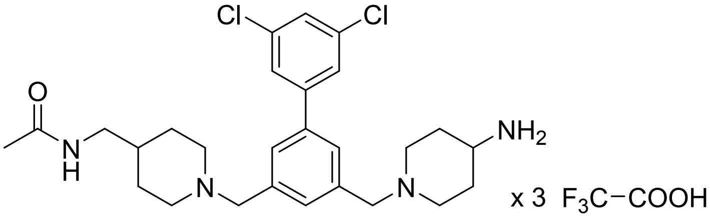


Compound **8** (100 mg, 0.24 mmol, 1 eq), *N*-(piperidin-4-ylmethyl)acetamide × HCl (47.12 mg, 0.24 mmol, 1 eq), 4-(Boc-Amino)-piperidin (49 mg, 0.24 mmol, 1 eq) and DIPEA (128 µL, 0.73 mmol, 3 eq) were dissolved in 20 mL of MeCN. The solution was stirred at 75 °C for 4 h. The solvent was removed in vacuo and the approach was mixed with 2 mL TFA. The product was precipitated in diethylether after 1 h and purified by preparative HPLC. Yield: 62.2 mg (30.8 %) of compound **29** as a white lyophilized solid. HPLC: 22.81 min, start at 10 %B (purity 99.6 %). $^{1}$H-NMR (500 MHz, DMSO-$d_6$) δ 10.36 (s, 1H), 9.82 (s, 1H), 8.17 (s, 3H), 8.00 (d, J = 8.1 Hz, 2H), 7.92 (t, J = 5.9 Hz, 1H), 7.78 (s, 2H), 7.71 (t, J = 1.8 Hz, 1H), 7.57 (s, 1H), 4.43 – 4.25 (m, 4H), 3.54 – 3.00 (m, 4H), 3.33 – 3.25 (m, 2H), 3.20 –

3.01 (m, 2H), 2.99 – 2.89 (m, 3H), 2.17 – 2.05 (m, 2H), 1.86 – 1.70 (m, 7H), 1.71 – 1.61 (m, 1H), 1.42 – 1.29 (m, 2H) ppm. MS (ESI, positive): calcd, 503.23; *m/z* 503.21 [M+H]$^+$.

*N-(1-((5-((4-(acetamidomethyl)piperidin-1-yl)methyl)-3',5'-dichloro-[1,1'-biphenyl]-3-yl)methyl)piperidin-4-yl)-2-aminoacetamide × 3 TFA (**30**).*

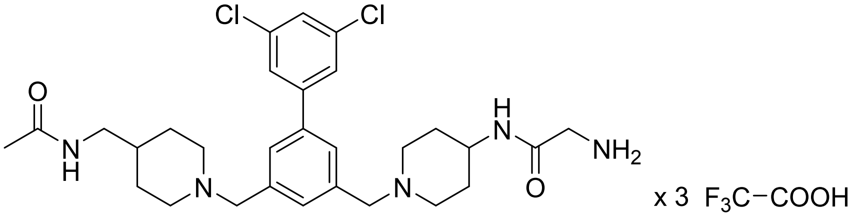


Inhibitor **29** (30 mg, 0.035 mmol, 1 eq), Boc-Gly-OH (6.2 mg, 0.035 mmol, 1 eq), HATU (13.5 mg, 0.035 mmol, 1 eq) and DIPEA (18.5 µL, 0.11 mmol, 3 eq) were dissolved in 5 mL DMF and stirred for 2 h. The solvent was removed in vacuo and the remaining residue was treated with 2 mL TFA. The product was precipitated in diethyl ether and purified by preparative HPLC. Yield: 25.5 mg (0.028 mmol, 80 %) of compound **30** as a white lyophilized solid. HPLC: 22.49 min, start at 10 %B (purity > 99 %). $^1$H NMR (500 MHz, DMSO-*d$_6$)* δ 10.40 (s, 1H), 9.92 (s, 1H), 8.52 (d, J = 7.2 Hz, 1H), 8.06 – 7.95 (m, 5H), 7.92 (t, J = 6.0 Hz, 1H), 7.78 (d, J = 1.9 Hz, 2H), 7.70 (t, J = 1.8 Hz, 1H), 7.58 (s, 1H), 4.35 (s, 4H), 3.90 – 3.78 (m, 1H), 3.52 (d, J = 5.1 Hz, 2H), 3.44 (t, J = 13.2 Hz, 4H), 3.25 – 3.04 (m, 3H), 2.98 – 2.89 (m, 3H), 1.99 (d, J = 13.5 Hz, 2H), 1.84 – 1.73 (m, 5H), 1.71 – 1.61 (m, 3H), 1.42 – 1.27 (m, 2H) ppm. MS (ESI, positive): calcd, 560.26; *m/z* 560.25 [M+H]$^+$.

*N-(1-((5-((4-(acetamidomethyl)piperidin-1-yl)methyl)-3',5'-dichloro-[1,1'-biphenyl]-3-yl)methyl)piperidin-4-yl)-3-aminopropanamide × 3 TFA (**31**).*

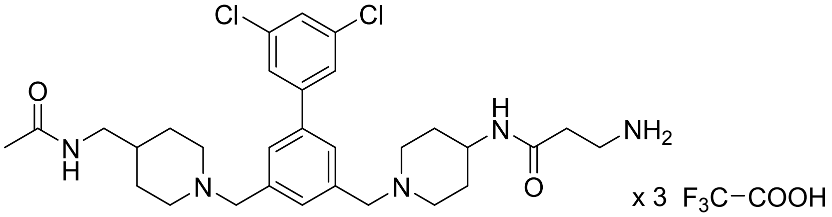


Inhibitor **29** (20 mg, 0.024 mmol, 1 eq), Boc-β-Ala-OH (4.48 mg, 0.024 mmol, 1 eq), HATU (9 mg, 0.024 mmol, 1 eq) and DIPEA (12.4 µL, 0.072 mmol, 3 eq) were dissolved in 2 mL DMF and stirred for 1.5 h. The solvent was removed in vacuo and 2 mL was added to the remaining residue. The product was precipitated in diethyl ether and purified by preparative HPLC. Yield: 12 mg (0.013 mmol, 54.2 %) of compound **31** as a white lyophilized solid. HPLC: 22.77 min, start at 10 %B (purity >99 %). $^1$H NMR (500 MHz, DMSO-*d$_6$*) δ 10.24 (s, 1H), 9.82 (s, 1H), 8.24 (d, J = 8.0 Hz, 1H), 8.00 (s, 2H), 7.92 (t, J = 5.8 Hz, 1H), 7.79 (d, J = 1.8 Hz, 3H), 7.71 (t, J = 1.8 Hz, 2H), 7.58 (s, 1H), 4.34 (s, 4H), 3.51 – 3.34 (m, 9H),

3.04 – 2.87 (m, 5H), 2.43 (t, J = 7.1 Hz, 2H), 2.01 – 1.91 (m, 2H), 1.80 (s, 5H), 1.71 – 1.58 (m, 3H), 1.42 – 1.29 (m, 2H) ppm. MS (ESI, positive): calcd, 574.27; *m/z* 574.26 $[M+H]^+$.

*N-((1-((5-((4-(aminomethyl)piperidin-1-yl)methyl)-3',5'-dichloro-[1,1'-biphenyl]-3-yl)methyl)piperidin-4-yl)methyl)acetamide × 3 TFA (**32**).*

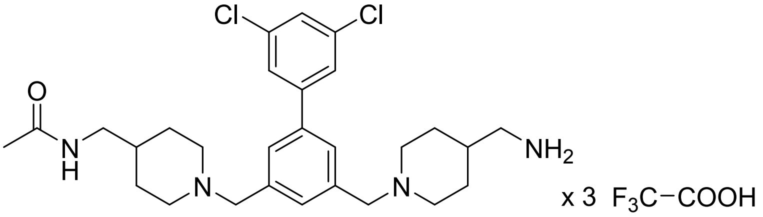


A solution of compound **8** (150 mg, 0.37 mmol, 1 eq), *N*-(piperidin-4-ylmethyl)acetamide × HCl (70.7 mg, 0.37 mmol, 1 eq), *tert*-butyl (piperidin-4-ylmethyl)carbamate (76.6 mg, 0.37 mmol, 1 eq) and DIPEA (192 µL, 1.1 mmol, 3 eq) was stirred for 9 h at 75 °C. The solvent was removed in vacuo and 2 mL TFA was added to the remaining residue. The product was precipitated after 1 h in diethylether and purified by preparative HPLC. Yield: 77 mg (0.089 mmol, 24 %) of compound **32** as a white lyophilized solid. HPLC: 22.33 min, start at 10 % B (purity 99.5 %). $^1H$ NMR (500 MHz, DMSO-$d_6$) δ 10.07 (s, 1H), 9.83 (s, 1H), 8.06 – 7.94 (m, 3H), 7.99 – 7.84 (m, 3H), 7.78 (d, J = 1.6 Hz, 2H), 7.71 (t, J = 1.7 Hz, 1H), 7.58 (s, 1H), 4.44 – 4.24 (m, 4H), 3.57 – 3.36 (m, 4H), 3.30 – 3.09 (m, 1H), 3.06 – 2.83 (m, 5H), 2.86 – 2.65 (m, 2H), 2.01 – 1.89 (m, 2H), 1.89 – 1.70 (m, 6H), 1.74 – 1.59 (m, 1H), 1.55 – 1.24 (m, 4H) ppm. $^{13}C$ NMR (126z MHz, DMSO-D6) δ 169.2, 158.3 (q, $^2J_{C\text{-}F}$ = 34.5 Hz, $CF_3COO^-$), 142.1, 134.9, 125.4, 117.4, 115.0, 58.8, 51.5, 51.0, 43.2, 43.0, 39.5, 33.5, 31.6, 31.4, 26.7, 26.3, 22.5 ppm. MS (ESI, positive): calcd, 517.25; *m/z* 517.29 $[M+H]^+$.

*4,4'-((3',5'-dichloro-[1,1'-biphenyl]-3,5-diyl)bis(methylene))bis(1-(1-methylpiperidin-4-yl)piperazine) × 6 TFA (**33**).*

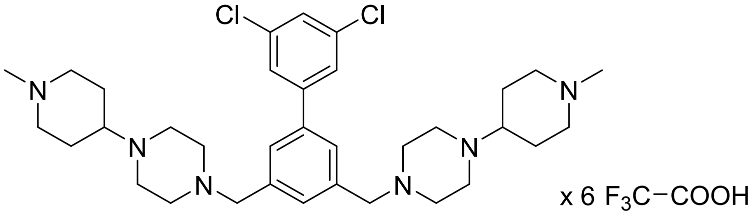


Compound **33** was isolated as a byproduct during the preparative HPLC purification of compound **26**. Yield: 9.6 mg (7.4 µmol, 3 %) of compound **33** as colorless solid. HPLC: 21.48 min, start at 10 % B (purity: > 99 %). $^1H$-NMR (500 MHz, DMSO-$d_6$): δ = 9.91 (s, 2H), 7.96 – 7.82 (m, 2H), 7.75 (d, J = 1.8 Hz, 2H), 7.67 (t, J = 1.8 Hz, 1H), 7.57 – 7.47 (m, 1H), 4.48 – 3.83 (m, 4H), 3.51 (d, J = 13.3 Hz, 4H), 3.37 – 2.64 (m,

28H), 2.20 – 1.98 (m, 4H), 1.80 – 1.52 (m, 4H) ppm. HRMS (APCI, positive): calcd, 612.3474; m/z, 613.3530 $[M+H]^+$.

*4,4'-((3',5'-dichloro-[1,1'-biphenyl]-3,5-diyl)bis(methylene))bis(1-(pyridin-4-yl)piperazine) × 4 TFA (**34**).*

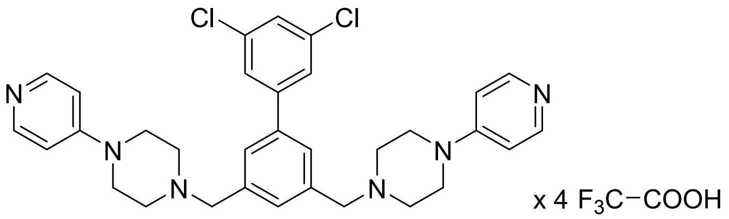


Compound **34** was obtained as additional product during the synthesis and preparative HPLC purification of compound **27**. Yield: 2.8 mg (0.002 mmol, 1 %) of compound **27** as a colorless solid. Due to the poor yield, the compound was resynthesized by treatment of compound **8** (50 mg, 0.122 mmol, 1 equiv) with 1-(pyridin-4-yl)piperazine (40 mg, 0.245 mmol, 2 equiv) and $NEt_3$ (34 µL, 0.245 mmol, 2 equiv) in 20 mL acetonitrile for 70 h at 70 °C. Afterwards the solution was acidified, the solvent removed in vacuo. The crude product was purified by preparative HPLC. Yield: 11.8 mg (0.009 mmol, 8 %) of compound **27** as a colorless solid. HPLC: 11.81 min, start at 20 % B (purity 95.7 %). $^1$H-NMR (500 MHz, DMSO-$d_6$) δ = 8.35 (d, J = 7.5 Hz, 4H), 7.92 (s, 2H), 7.77 (d, J = 1.9 Hz, 2H), 7.69 (t, J = 1.8 Hz, 1H), 7.58 (s, 1H), 7.24 (d, J = 7.7 Hz, 4H), 4.22 (s, 4H), 3.15 (s, 8H) ppm. Missing aliphatic signal is probably covered by water signal. $^{13}$C-NMR (126 MHz, DMSO-$d_6$) δ = 156.7, 142.4, 140.2, 137.8, 134.8, 132.7, 129.6, 127.4, 125.4, 107.9, 59.2, 50.5, 43.8 ppm and signals of TFA: 158.3 (q, J = 32.8 Hz), 117.9, 115.5 ppm. HRMS (APCI, positive): calcd, 572.2222; m/z 573.2285.

*1,1'-((3',5'-dichloro-[1,1'-biphenyl]-3,5-diyl)bis(methylene))dipiperazine × 4 TFA (**35**).*

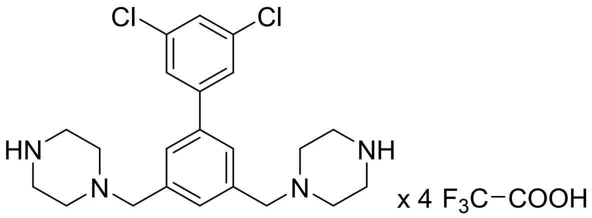


Compound **8** (99.2 mg, 0.243 mmol, 1.0 equiv) was dissolved with 1-Boc-piperazine (90.4 mg, 0.485 mmol, 2.0 equiv) in 5 mL acetonitrile, treated with $NEt_3$ (67 µL, 0.485 mmol, 2.0 equiv) and stirred at 70 °C for 19 h. The solvent was removed in vacuo and the residue was treated with 1 mL TFA and stirred for 1 h at room temperature. After precipitation in cold diethyl ether the crude product was purified by preparative HPLC and lyophilized. Yield: 117.0 mg (0.134 mmol, 55 %) of compound **35** as a colorless crystalline solid. HPLC: 21.15 min, start at 10 % B (purity: > 99 %). $^{1}$H-NMR (500 MHz, water-*d₂*): δ = 7.95 (s, 2H), 7.74 (s, 1H), 7.71 – 7.64 (m, 2H), 7.62 – 7.56 (m, 1H), 4.65 (s, 4H), 3.81 – 3.59 (m, 16H) ppm. (500 MHz, DMSO- $d_6$): δ = 9.07 (s, 4H), 7.77 (s, 2H), 7.75 (d, J = 1.9 Hz, 2H), 7.65 (t, J = 1.9 Hz, 1H), 7.50 (s, 1H), 3.96 (s, 4H), 3.24 (s, 8H), 2.93 (s, 8H) ppm. $^{13}$C-NMR (126 MHz, Methanol-$d_4$): δ = 144.5, 140.9, 136.7, 136.4, 132.9, 130.3, 128.7, 126.8, 62.0, 50.1, 43.6 ppm and signals of TFA: 162.6 (q, J = 36.5 Hz), 119.0, 116.7 ppm. (126 MHz, DMSO-$d_6$): δ = 142.9, 137.6, 134.7, 131.6, 128.4, 127.2, 125.4, 60.0, 48.5, 41.8 ppm and signals of TFA: 158.6 (q, J = 33.1 Hz), 117.8, 115.4 ppm. The missing aromatic signal is presumably covered by another signal or the noise. HRMS (ESI, positive): calcd, 418.1691; m/z, 419.1759 $[M+H]^+$.

*1,1'-((3',5'-dichloro-[1,1'-biphenyl]-3,5-diyl)bis(methylene))bis(piperidin-4-amine) × 4 TFA (**36**).*

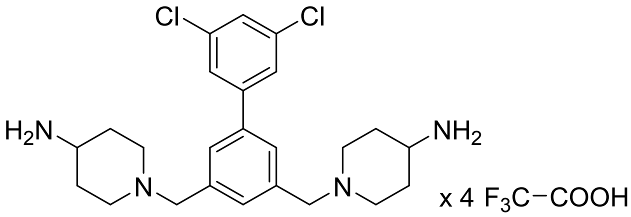


Compound **8** (97.1 mg, 0.237 mmol, 1.0 equiv) and 4-(*N*-Boc-amino)piperidine (95.1 mg, 0.475 mmol, 2.0 equiv) were dissolved in 5 mL acetonitrile and treated with $NEt_3$ (66 µL, 0.475 mmol, 2.0 equiv). The solution was stirred at 70 °C for 19 h. The solvent was removed in vacuo. 1 mL of TFA was added to the crude intermediate and stirred for 1 h at room temperature. After precipitation in cold diethyl ether the crude product was purified by preparative HPLC and lyophilized. Yield: 136.9 mg (0.156 mmol, 66 %) of compound **36** as a colorless crystalline solid. HPLC: 19.46 min, start at 10 % B (purity: > 99 %). $^{1}$H-NMR (500 MHz, water-$d_2$): δ = 7.91 (s, 2H), 7.69 (s, 1H), 7.68 – 7.66 (m, 2H), 7.59 –

7.57 (m, 1H), 4.52 (s, 4H), 3.75 (d, J = 13.2 Hz, 4H), 3.68 – 3.57 (m, 2H), 3.27 (t, J = 13.5 Hz, 4H), 2.40 (d, J = 14.5 Hz, 4H), 2.14 – 1.90 (m, 4H) ppm. (500 MHz, DMSO-$d_6$): δ = 10.64 (br s, 2H), 8.26 (br s, 6H), 7.96 (br s, 2H), 7.76 (d, J = 1.9 Hz, 2H), 7.69 (t, J = 1.8 Hz, 1H), 7.55 (br s, 1H), 4.32 (br s, 4H), 3.72 – 2.83 (m, 10H), 2.22 – 1.98 (m, 4H), 1.91 – 1.63 (m, 4H) ppm. $^{13}$C-NMR (126 MHz, Methanol-$d_4$): δ = 143.6, 141.6, 136.9, 135.1, 132.6, 129.1, 126.9, 60.8, 51.6, 46.9, 28.4 ppm and signals of TFA: 163.1 (q, J = 34.6 Hz), 118.1 (q, J = 292.0 Hz) ppm. HRMS (ESI, positive): calcd, 446.2004; m/z, 447.2072 [M+H]$^+$.

*1,1'-((3',5'-dichloro-[1,1'-biphenyl]-3,5-diyl)bis(methylene))dipiperidine × 2 TFA (**37**).*

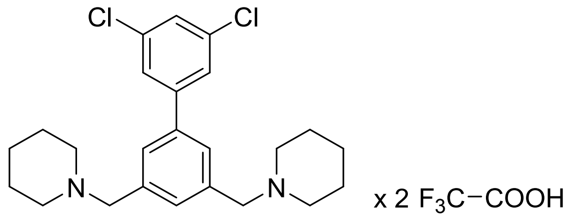


Compound **8** (99.8 mg, 0.244 mmol, 1.0 equiv) and piperidine (48 µL, 0.488 mmol, 2.0 equiv) where dissolved in 5 mL acetonitrile, treated with $NEt_3$ (68 µL, 0.488 mmol, 2.0 equiv) and stirred at 70 °C for 19 h. The solvent was removed in vacuo and the crude product was purified by preparative HPLC and lyophilized. Yield: 104.1 mg (0.161 mmol, 66 %) of compound **37** as a colorless crystalline solid. HPLC: 20.61 min, start at 20 % B (purity: > 99 %). $^1$H-NMR (500 MHz, water-$d_2$): δ = 7.83 – 7.74 (m, 2H), 7.67 – 7.61 (m, 1H), 7.59 (d, J = 1.8 Hz, 2H), 7.45 (t, J = 1.8 Hz, 1H), 4.42 (s, 4H), 3.54 (d, J = 12.4 Hz, 4H), 3.12 – 2.95 (m, 4H), 1.99 (d, J = 15.1 Hz, 4H), 1.90 – 1.83 (m, 2H), 1.81 – 1.69 (m, 4H), 1.59 – 1.42 (m, 2H) ppm. (500 MHz, DMSO-$d_6$): δ = 10.08 (br s, 2H), 8.01 (d, J = 1.3 Hz, 2H), 7.78 (d, J = 1.9 Hz, 2H), 7.69 (t, J = 1.9 Hz, 1H), 7.62 (s, 1H), 4.36 (s, 4H), 3.39 (d, J = 12.1 Hz, 4H), 3.02 – 2.83 (m, 4H), 1.93 – 1.59 (m, 10H), 1.48 – 1.28 (m, 2H) ppm. $^{13}$C-NMR (126 MHz, Methanol-$d_4$): δ = 143.7, 141.4, 136.9, 135.1, 132.6, 129.1, 126.9, 61.1, 54.3, 24.1, 22.7 ppm and signals of TFA: 162.9 (q, J = 35.1 Hz) ppm. The missing aromatic signal is presumably covered by another signal or the noise. (126 MHz, DMSO-$d_6$): δ = 142.1, 137.9, 134.8, 134.2, 131.1, 130.9, 127.5, 125.4, 58.6, 51.9, 22.3, 21.2 ppm and signals of TFA: 158.4 (q, J = 31.7 Hz) ppm. HRMS (ESI, positive): calcd, 416.1786; m/z, 417.1867 [M+H]$^+$.

*1,1'-((3',5'-Dichlor-[1,1'-biphenyl]-3,5-diyl)bis(methylen))dipyrrolidin × 2 TFA (**38**).*

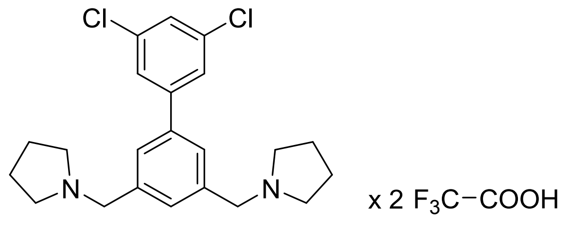

Compound **8** (98.6 mg, 0.241 mmol, 1.0 equiv) and pyrrolidine (40 µL, 0.482 mmol, 2.0 equiv) where dissolved in 5 mL acetonitrile, treated with $NEt_3$ (67 µL, 0.482 mmol, 2.0 equiv) and stirred at 70 °C for 19 h. The solvent was removed in vacuo and the crude product was purified by preparative HPLC and lyophilized. Yield: 40.9 mg (0.066 mmol, 27 %) of compound **38** as a colorless crystalline solid. HPLC: 18.25 min, start at 20 % B (purity: 98.3 %). $^{1}$H-NMR (500 MHz, water-*d*$_2$): δ = 7.87 (s, 2H), 7.69 (s, 1H), 7.69 – 7.66 (m, 2H), 7.60 – 7.58 (m, 1H), 4.55 (s, 4H), 3.70 – 3.46 (m, 4H), 3.42 – 3.20 (m, 4H), 2.42 – 2.18 (m, 4H), 2.13 – 1.95 (m, 4H) ppm. (500 MHz, DMSO-*d*$_6$): δ = 10.35 (s, 2H), 8.02 (s, 2H), 7.80 (d, J = 1.9 Hz, 2H), 7.70 (t, J = 1.9 Hz, 1H), 7.65 (s, 1H), 4.43 (s, 4H), 3.60 – 2.99 (m, 8H), 2.33 – 1.46 (m, 8H) ppm. $^{13}$C-NMR (126 MHz, Methanol-*d*$_4$): δ = 143.7, 141.7, 137.0, 134.4, 133.3, 131.6, 129.2, 126.9, 58.6, 55.1, 23.9 ppm. (126 MHz, DMSO-*d*$_6$): δ = 142.2, 138.0, 134.9, 132.9, 132.5, 129.9, 127.5, 125.4, 56.5, 53.0, 22.5 ppm and signals of TFA: 158.6 (q, J = 33.0 Hz), 117.9, 115.5 ppm. HRMS (ESI, positive): calcd, 388.1473; m/z, 389.1545 $[M+H]^+$.

The modified synthesis of analog **39** is described in Scheme S2.

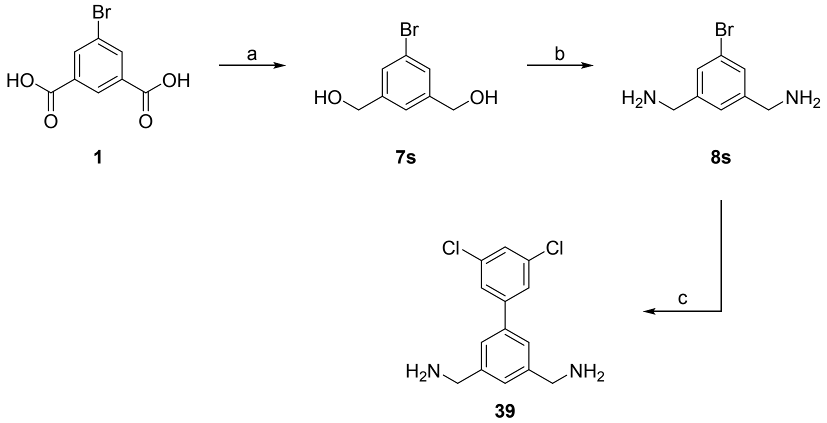


**Scheme S2.** (a) 1.0 equiv. of **1**, 4.0 equiv. $BH_3$-THF in THF, at 0 °C over 10 min, then room temperature overnight, 78 %; (b) (i) 1.0 equiv. of **7s,** 4.0 equiv. of $NEt_3$, 2.4 equiv. of mesyl chloride in ethyl acetate, at -5 °C over 10 min, then room temperature; (ii) 4.6 equiv. of $NaN_3$ in DMF at room temperature over 24 h; (iii) 4.4 equiv. of $PPh_3$ in ethyl acetate at 70°C over 3.5 h, then room temperature; (iv) water/DMF (1:1, v/v) at room temperature, overnight, 57 %; (c) 1.00 equiv. of **8s** 3,5-dichlorophenylboronic acid, 0.06 equiv. of $Pd(PPh_3)_4$, 8.25 equiv. of $Na_2CO_3$ in dioxane/water (3.5:1, v/v) at 100 °C over 2 h, 30 %.

*(3',5'-dichloro-[1,1'-biphenyl]-3,5-diyl)dimethanamine × 2 TFA (**39**).*

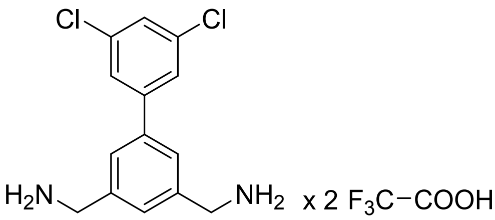


Compound **8s** (110 mg, 0.248 mmol, 1.00 equiv), 3,5-dichlorophenylboronic acid (117 mg, 0.613 mmol, 2.47 equiv), $Pd(PPh_3)_4$ (18 mg, 0.016 mmol, 0.06 equiv), and $Na_2CO_3$ (217 mg, 2.047 mmol, 8.25 equiv) were added to a mixture of 7 mL of dioxane and 2 mL of water. The mixture was degassed with argon and heated under stirring for 2 h at 100 °C in a laboratory microwave, and then cooled to room temperature. After adding some mL of water and acidifying with 5 % $KHSO_4$, the mixture was filtered. The solvent was removed in vacuo and the crude product was purified by preparative HPLC and lyophilized. Yield: 38.1 mg (0.075 mmol, 30 %) of compound **39** obtained as a white solid. HPLC: 22.23 min, start at 10 % B (purity: > 99 %). $^1$H-NMR (500 MHz, DMSO-$d_6$): δ = 8.35 (br s, 6H), 7.92 (s, 2H), 7.79 (d, J = 1.8 Hz, 2H), 7.69 (t, J = 1.8 Hz, 1H), 7.52 (s, 1H), 4.13 (s, 4H) ppm. $^{13}$C-NMR (126 MHz, DMSO-$d_6$) δ = 142.4, 137.4, 135.3, 134.9, 129.5, 127.4, 127.1, 125.1, 42.0 ppm. HRMS (ESI, positive): calcd, 280.0534; m/z, 281.0605 $[M+H]^+$.

Inhibitors **40**-**46** have been synthesized as described in Scheme S3.

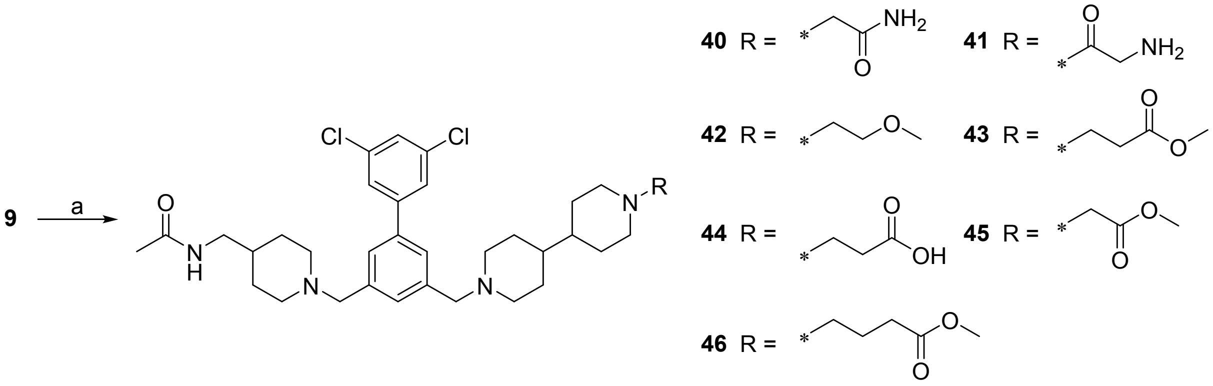


**Scheme S3.** (a) For compound **40**: 1.2 equiv. 2-bromoacetamide, 4.0 equiv. $K_2CO_3$ in DMF, 2 h 80 °C, 59 %; **41**: (i) 1.0 equiv. Boc-Gly-OH, 1.0 equiv. HATU, 6.0 equiv. DIPEA in DMF, overnight at room temperature, (ii) TFA, 1 h at room temperature, 81 %; **42**: 1.2 equiv. 1-bromo-2-methoxyethane, 3.0 equiv. $K_2CO_3$ in DMF, 3 h at 80 °C, 73 %; **43**: 1.3 equiv. 3-bromopropanoate, 3.0 equiv. $K_2CO_3$ in DMF, 2 h at 80 °C, 72 %; **44**: compound **43** and 1 M LiOH in dioxane/acetone, 3 h at room temperature, 73 %; **45**: 1.2 equiv. methyl bromoacetate, 3.0 equiv. $K_2CO_3$ in DMF, 3 h at 80 °C, 21 %; **46**: 2.4 equiv. methyl butanoate, 6.0 equiv. $K_2CO_3$ in DMF, 4 h at 80 °C, 56 %.

*2-(1'-((5-((4-(acetamidomethyl)piperidin-1-yl)methyl)-3',5'-dichloro-[1,1'-biphenyl]-3-yl)methyl)-[4,4'-bipiperidin]-1-yl)acetamide × 3 TFA (**40**).*

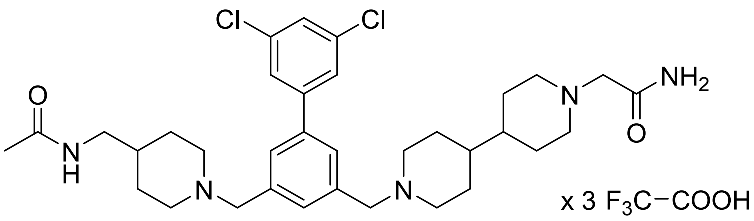


Compound **9** (70.0 mg, 0.077 mmol, 1.0 equiv) was dissolved with 2-bromoacetamide (12.7 mg, 0.092 mmol, 1.2 equiv) and $K_2CO_3$ (31.8 mg, 0.230 mmol, 3.0 equiv) in 300 µL DMF. The solution was stirred for 2 h at 80 °C. For complete conversion, additional 10.2 mg $K_2CO_3$ (1.0 equiv), 5 mg 2-bromoacetamide (0.5 equiv), and 200 µL DMF were added and the mixture stirred for another 2 h at 80 °C. After cooling to room temperature, the product was purified by preparative HPLC and lyophilized. Yield: 44.1 mg (0.045 mmol, 59 %) of product **40** obtained as a colorless solid. HPLC: 13.96 min, start at 20 % B (purity: > 99 %). $^1$H-NMR (500 MHz, DMSO-$d_6$): δ = 10.40 – 9.84 (m, 2H), 9.74 – 9.30 (m, 1H), 8.00 (s, 2H), 7.97 – 7.89 (m, 2H), 7.78 (d, J = 1.8 Hz, 2H), 7.70 (t, J = 1.8 Hz, 1H), 7.65 (s, 1H), 7.60 (s, 1H), 4.35 (s, 4H), 3.83 (s, 2H), 3.54 – 3.32 (m, 6H), 3.25 – 3.07 (m, 1H), 3.05 – 2.78 (m, 7H), 1.95 – 1.74 (m, 9H), 1.71 – 1.58 (m, 1H), 1.56 – 1.15 (m, 8H) ppm. $^{13}$C-NMR (126 MHz, DMSO-$d_6$) δ = 169.2, 165.9, 142.1, 137.9, 134.9, 134.2, 131.1, 131.1, 130.9, 127.6, 125.4, 58.8, 56.5, 52.6, 51.7, 51.6, 43.2, 37.0, 36.8, 33.5, 26.7, 25.8, 25.6, 22.5 ppm and signals of TFA: 158.2 (q, J = 32.8 Hz), 117.9, 115.5 ppm. HRMS (APCI, positive): calcd, 627.3107; m/z, 628.3160 $[M+H]^+$.

The synthesis of inhibitors **41**-**44** is described in the main manuscript.

*Methyl 2-(1'-((5-((4-(acetamidomethyl)piperidin-1-yl)methyl)-3',5'-dichloro-[1,1'-biphenyl]-3-yl)methyl)-[4,4'-bipiperidin]-1-yl)acetate × 3 TFA (**45**).*

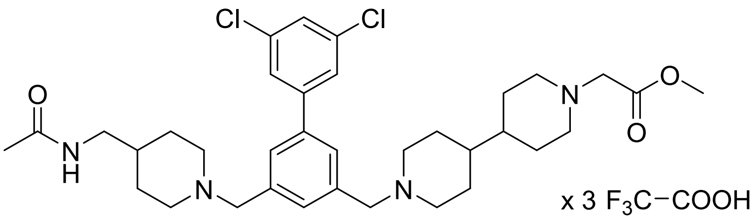


Compound **9** (70.0 mg, 0.077 mmol, 1.0 equiv) was dissolved with methyl bromoacetate (9.5 µL, 0.092 mmol, 1.2 equiv) and $K_2CO_3$ (31.8 mg, 0.230 mmol, 3.0 equiv) in 300 µL DMF. The solution was stirred for 3 h at 80 °C. After cooling to room temperature, the mixture was purified by preparative HPLC and

the product lyophilized. Yield: 15.7 mg (0.016 mmol, 21 %) of compound **45** obtained as a colorless resin-like solid. HPLC: 15.26 min, start at 20 % B (purity: > 99 %). $^{1}$H-NMR: (500 MHz, DMSO-$d_6$): δ = 10.35 – 9.78 (m, 3H), 8.02 – 7.96 (m, 2H), 7.93 (t, J = 6.2 Hz, 1H), 7.78 (d, J = 1.8 Hz, 2H), 7.70 (t, J = 1.8 Hz, 1H), 7.60 (s, 1H), 4.35 (s, 4H), 4.18 (s, 2H), 3.76 (s, 3H), 3.57 – 3.37 (m, 6H), 3.26 – 3.10 (m, 1H), 3.01 – 2.85 (m, 7H), 1.92 – 1.73 (m, 9H), 1.71 – 1.59 (m, 1H), 1.55 – 1.26 (m, 8H) ppm. $^{13}$C-NMR (126 MHz, DMSO-$d_6$): δ = 169.2, 166.2, 142.1, 137.9, 134.9, 134.2, 131.1, 131.1, 130.9, 127.6, 125.4, 58.8, 58.8, 55.2, 52.8, 52.7, 51.7, 51.6, 43.2, 37.0, 36.8, 33.5, 26.7, 25.8, 25.6, 22.5 ppm and signals of TFA: 158.3 (q, J = 33.4 Hz), 117.7, 115.3 ppm. HRMS (APCI, positive): calcd, 642.3103; m/z, 643.3162 $[M+H]^+$.

*Methyl 4-(1'-((5-((4-(acetamidomethyl)piperidin-1-yl)methyl)-3',5'-dichloro-[1,1'-biphenyl]-3-yl)methyl)-[4,4'-bipiperidin]-1-yl)butanoate × 3 TFA (**46**).*

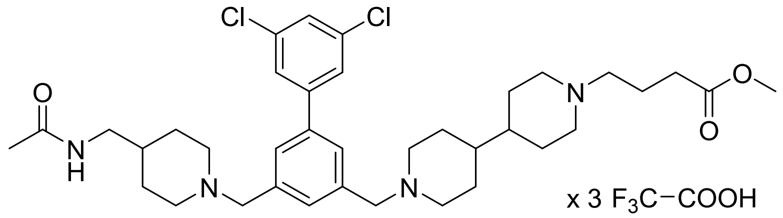


Compound **9** (73.5 mg, 0.080 mmol, 1.0 equiv) was dissolved with methyl butanoate (12 µL, 0.097 mmol, 1.2 eqiv) and $K_2CO_3$ (33.4 mg, 0.241 mmol, 3.0 equiv) in 300 µL DMF. The mixture was stirred for 2 h at 80 °C. For complete conversion, additional 33.4 mg of $K_2CO_3$ (3.0 equiv), 12 µL of methyl butanoate (1.2 equiv), and 200 µL of DMF were added and stirred for another 2 h at 80 °C. After cooling to room temperature, the mixture was purified by preparative HPLC and lyophilized. Yield: 45.6 mg (0.045 mmol, 56 %) of compound **46** obtained as a colorless resinous solid. HPLC: 16.41 min, start: 20 % B (purity: > 99 %). $^{1}$H-NMR (500 MHz, DMSO-$d_6$): δ = 10.30 – 9.63 (m, 2H), 9.69 – 9.15 (m, 1H), 8.00 (s, 2H), 7.93 (t, J = 5.9 Hz, 1H), 7.78 (d, J = 1.8 Hz, 2H), 7.70 (t, J = 1.8 Hz, 1H), 7.59 (s, 1H), 4.35 (s, 4H), 3.61 (s, 3H), 3.54 – 3.31 (m, 6H), 3.25 – 3.10 (m, 1H), 3.09 – 2.77 (m, 9H), 2.41 (t, J = 7.3 Hz, 2H), 1.96 – 1.73 (m, 11H), 1.71 – 1.57 (m, 1H), 1.49 – 1.27 (m, 8H) ppm. $^{13}$C-NMR (126 MHz, DMSO-$d_6$): δ = 172.4, 169.3, 142.1, 137.9, 134.9, 134.2, 131.1, 131.1, 130.9, 127.6, 125.4, 58.8, 58.8, 55.1, 51.8, 51.7, 51.6, 51.4, 43.2, 39.5, 37.0, 36.9, 33.5, 30.2, 26.7, 26.0, 25.8, 22.5, 18.9 ppm and signals of TFA: 158.3 (q, J = 34.9 Hz), 116.0 (q, J = 294.0 Hz) ppm. HRMS (APCI, positive): calcd, 670.3416; m/z, 671.3472 $[M+H]^+$.

Compound **9s** was obtained as side product during the synthesis of inhibitor **32** (see Scheme S1 below).

*(((3',5'-dichloro-[1,1'-biphenyl]-3,5-diyl)bis(methylene))bis(piperidine-1,4-diyl))dimethanamine × 4 TFA (**9s**).*

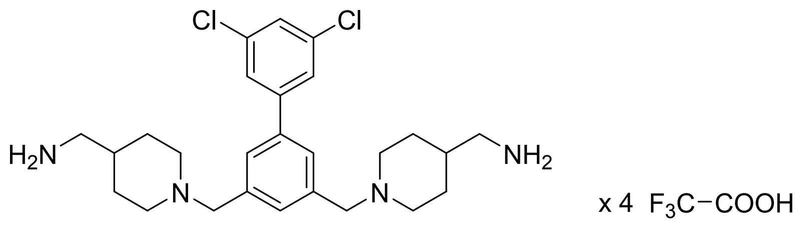


Inhibitor **9s** was isolated as a second product during the preparative purification of inhibitor **32**. Yield: 53 mg (0.056 mmol, 15 %) of compound **9s** as a white lyophilized solid. HPLC: 19.9 min, start at 10 % B (purity 99.4 %). $^1$H NMR (500 MHz, DMSO-$d_6$) δ 10.22 (s, 2H), 8.01 (s, 2H), 7.99 – 7.94 (m, 5H), 7.77 (d, J = 1.5 Hz, 2H), 7.70 (t, J = 1.8 Hz, 1H), 7.60 (s, 1H), 4.37 (s, 4H), 3.47 (d, J = 12.8 Hz, 4H), 3.06 – 2.91 (m, 4H), 2.80 – 2.67 (m, 4H), 1.99 – 1.89 (m, 4H), 1.89 – 1.79 (m, 2H), 1.50 – 1.35 (m, 4H) ppm. $^{13}$C NMR (126 MHz, DMSO-$d_6$) δ158.4 (q, $^2J_{C-F}$ = 33.7 Hz, $CF_3COO^-$), 142.1, 134.9, 130.9, 127.6, 125.4, 120.4, 117.6, 115.2, 112.9, 58.8, 51.0, 43.1, 39.5, 31.6, 26.3 ppm. MS (ESI, positive): calcd, 474.23; *m/z* 475.28 $[M+H]^+$.

**3. Synthesis scheme and HPLC chromatograms for the preparation of inhibitor 32**

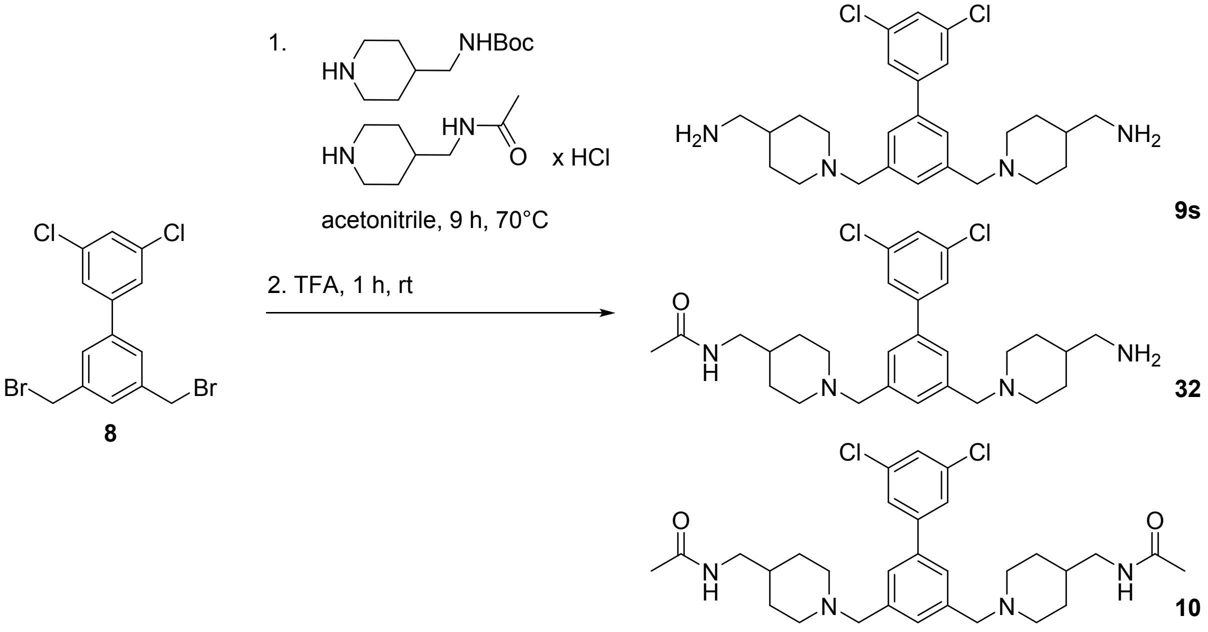


**Scheme S4.** Synthesis of the asymmetric inhibitor **32** providing the two additional symmetric derivatives **9s** and **10**.

The following analytical HPLC chromatograms are reaction controls of the two final steps for the preparation of inhibitor **32** according to Scheme S4.

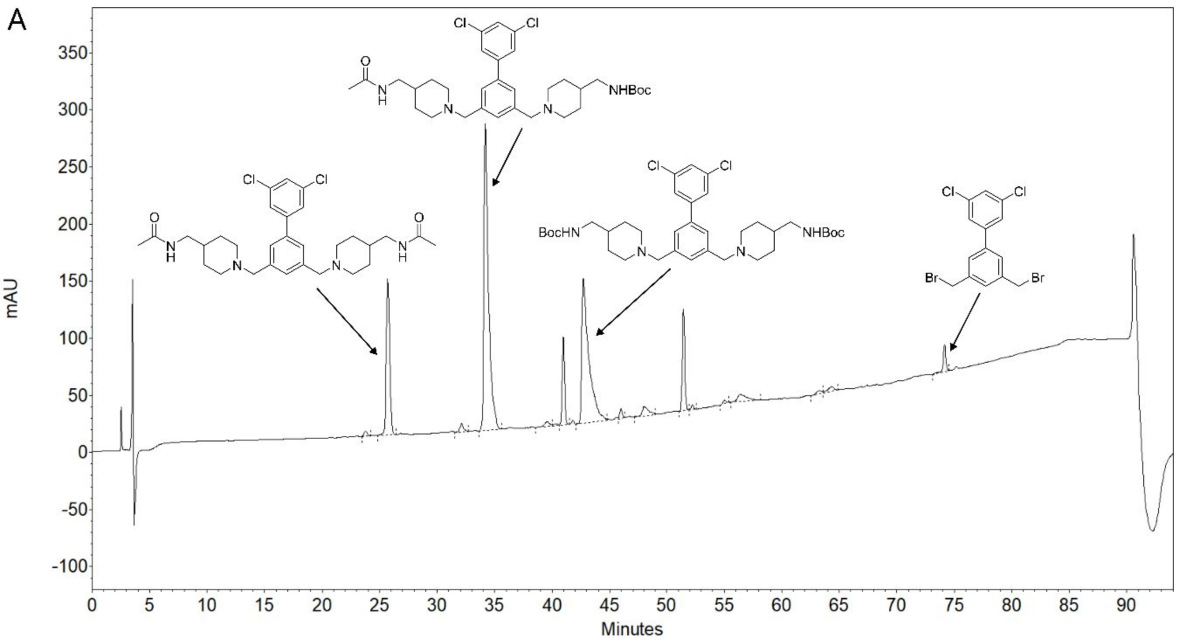

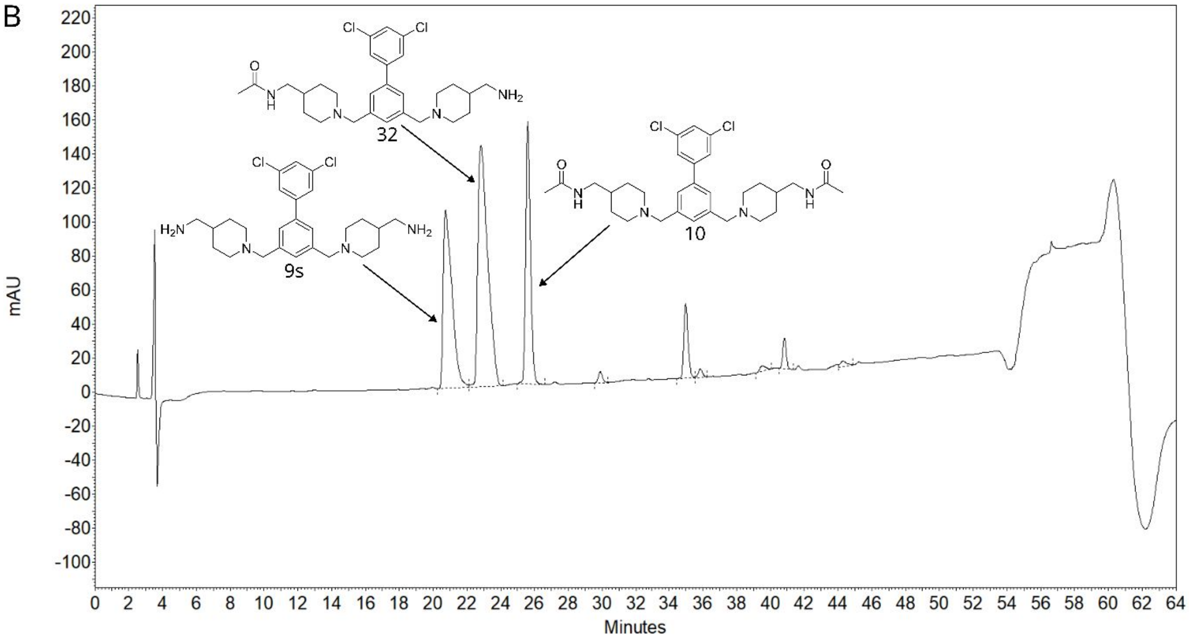


**Figure S1.** Analytical HPLC chromatograms of the two final steps for the synthesis of inhibitor **32** according to Scheme S4. (A) Step 1 provided the indicated three major products. The asymmetric Boc-protected precursor of inhibitor **32** elutes at approximately 34.2 min between the two symmetric compounds (HPLC method: linear gradient, start at 10 % solvent B to 90 % solvent B within 80 min, detection at 220 nm. After 80 min, the column wash starts). (B) Treatment with TFA in step 2 provided the asymmetric inhibitor **32**, which again elutes between the symmetric compounds **9s** and **10** (HPLC method: linear gradient, start at 10 % solvent B to 60 % solvent B within 50 min, detection at 220 nm. After 50 min, the column wash starts).

## 4. HPLC chromatograms of inhibitors

### Compound 4

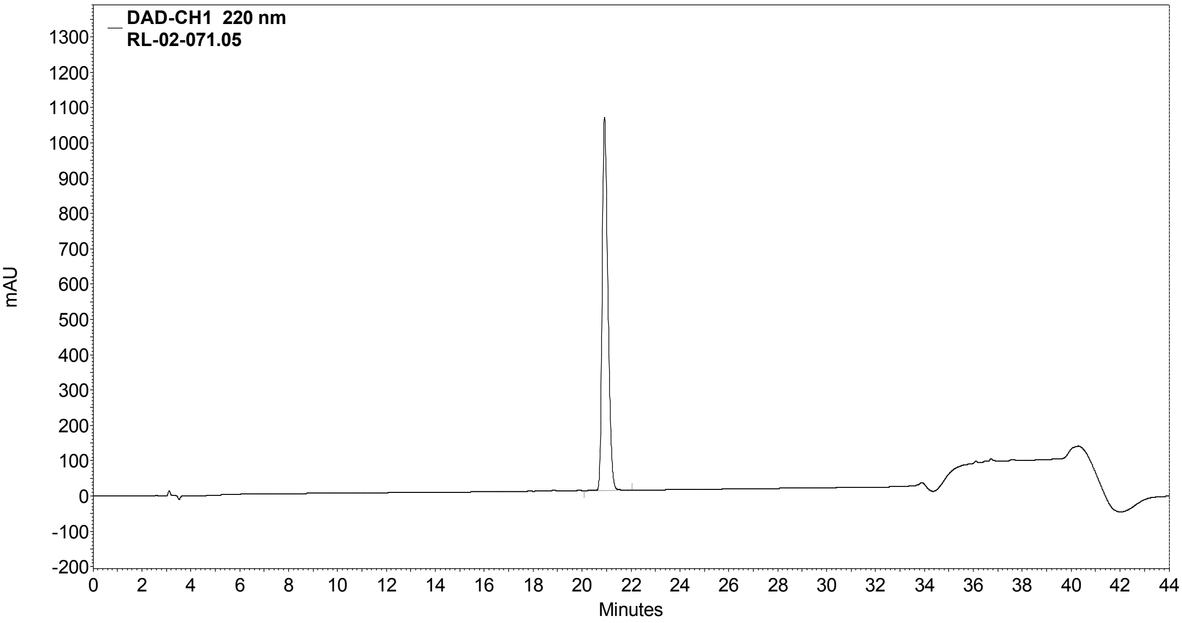


20 % solvent B to 50 % solvent B in 30 min, the compound elutes at 20.92 min. After 30 min, the washing procedure starts.

### Compound 5

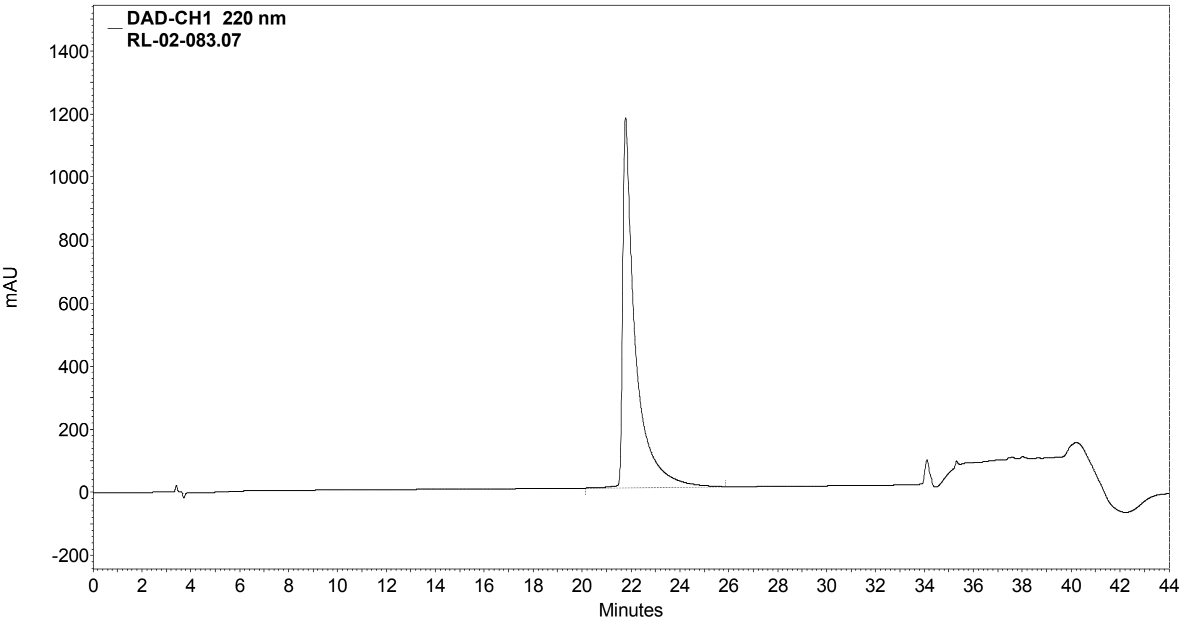


10 % solvent B to 40 % solvent B in 30 min, the compound elutes at 21.78 min. After 30 min, the washing procedure starts.

**Compound 6**

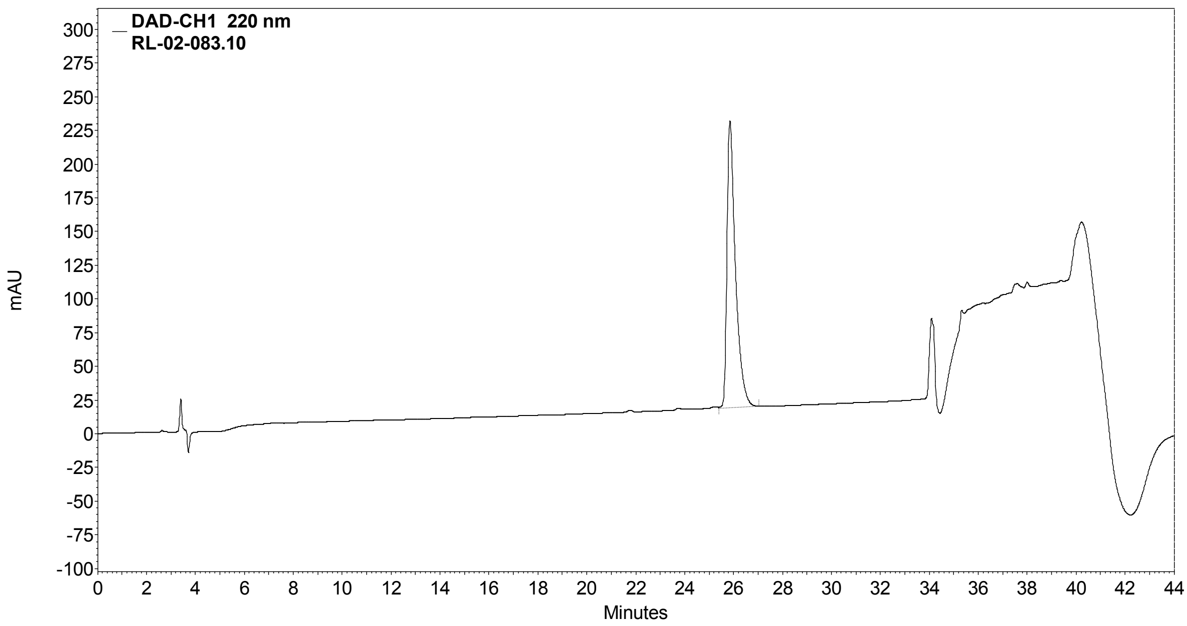


10 % solvent B to 40 % solvent B in 30 min, the compound elutes at 25.85 min. After 30 min, the washing procedure starts.

**Compound 9**

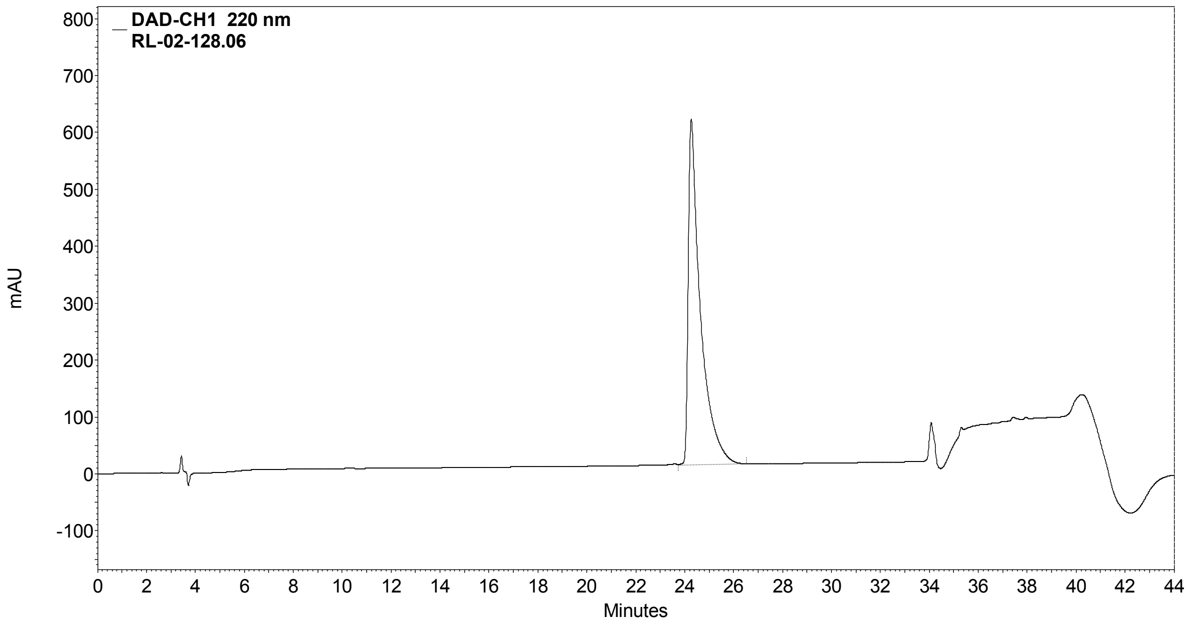


10 % solvent B to 40 % solvent B in 30 min, the compound elutes at 24.27 min. After 30 min, the washing procedure starts.

**Compound 10**

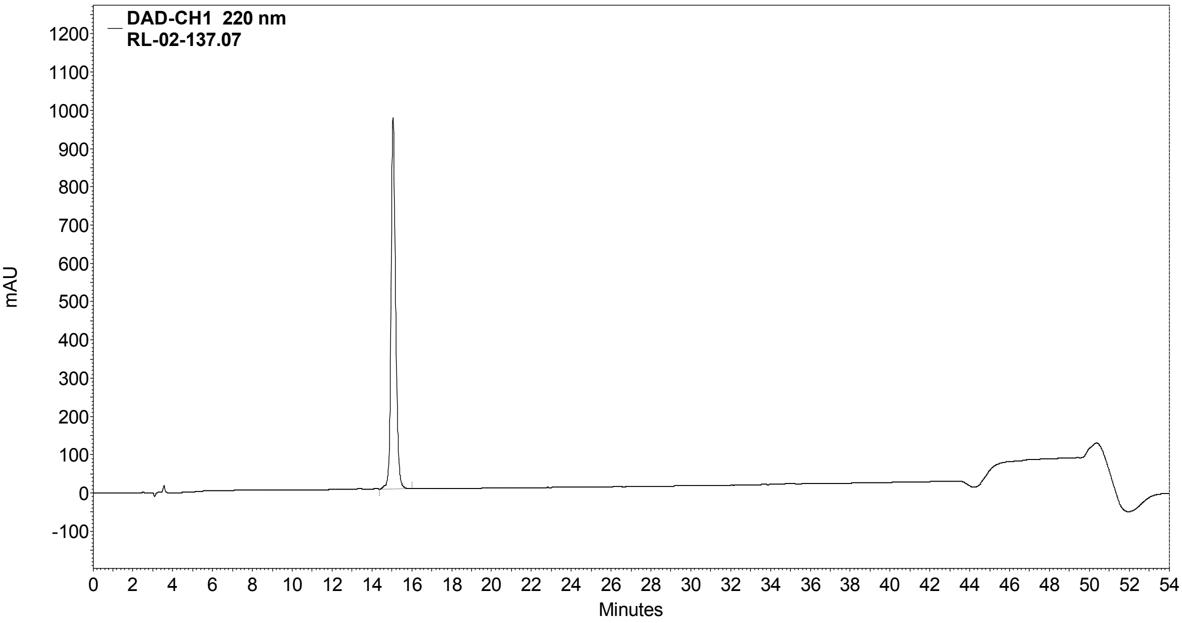


20 % solvent B to 60 % solvent B in 40 min, the compound elutes at 15.05 min. After 40 min, the washing procedure starts.

**Compound 11**

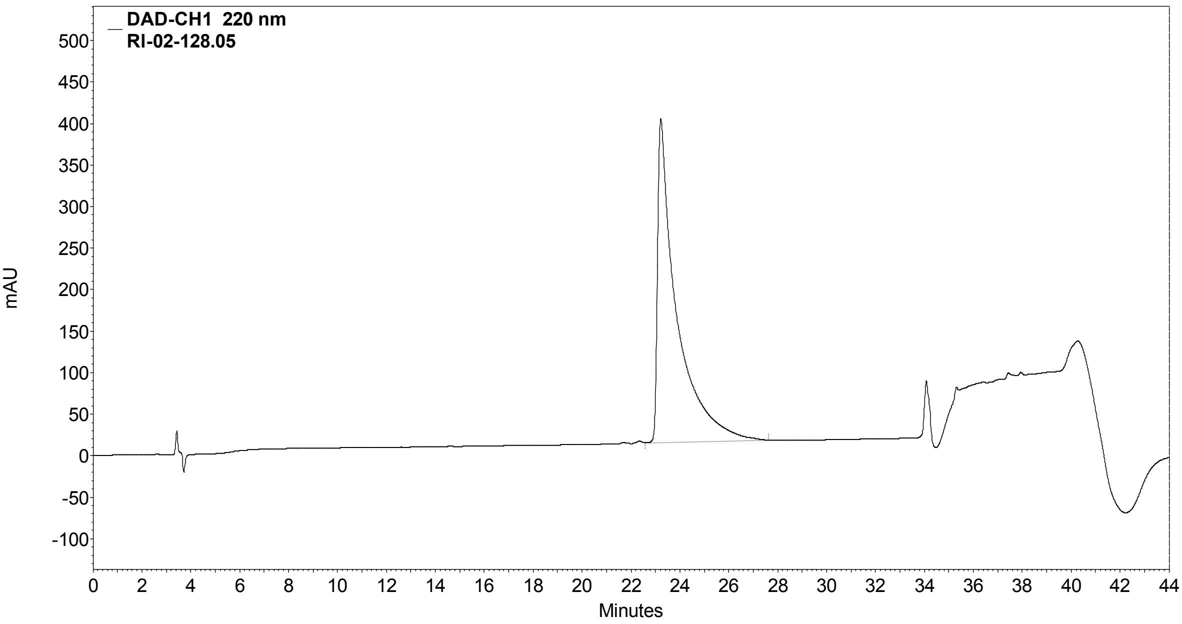


10 % solvent B to 40 % solvent B in 30 min, the compound elutes at 23.22 min. After 30 min, the washing procedure starts.

**Compound 12**

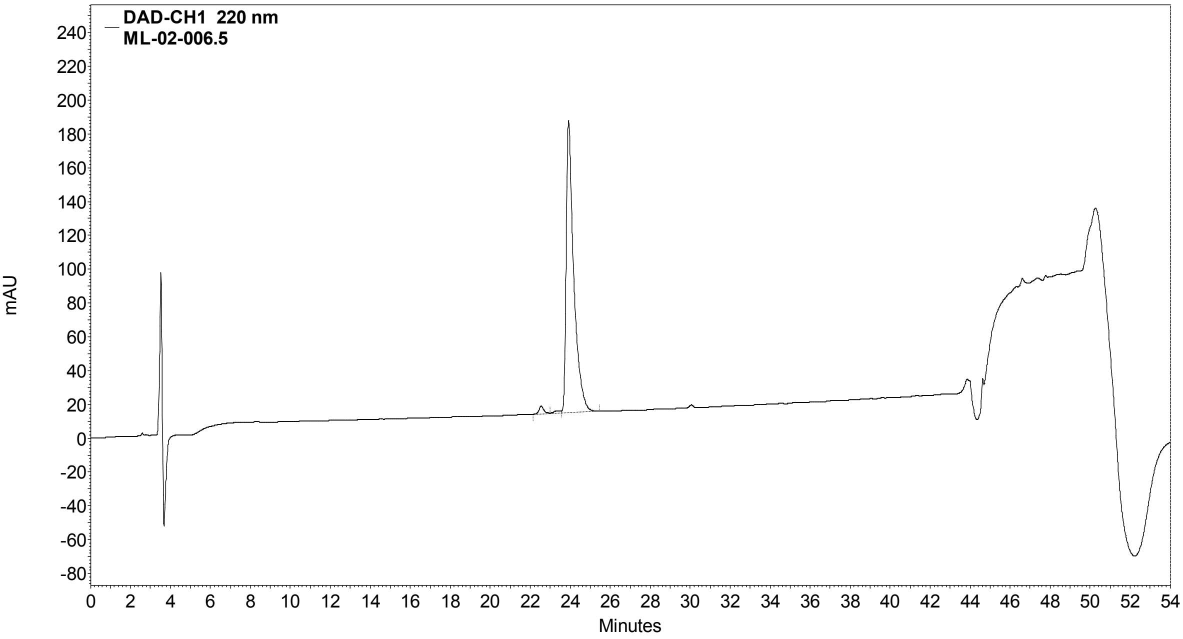


10 % solvent B to 50 % solvent B in 40 min, the compound elutes at 23.92 min. After 40 min, the washing procedure starts.

**Compound 13**

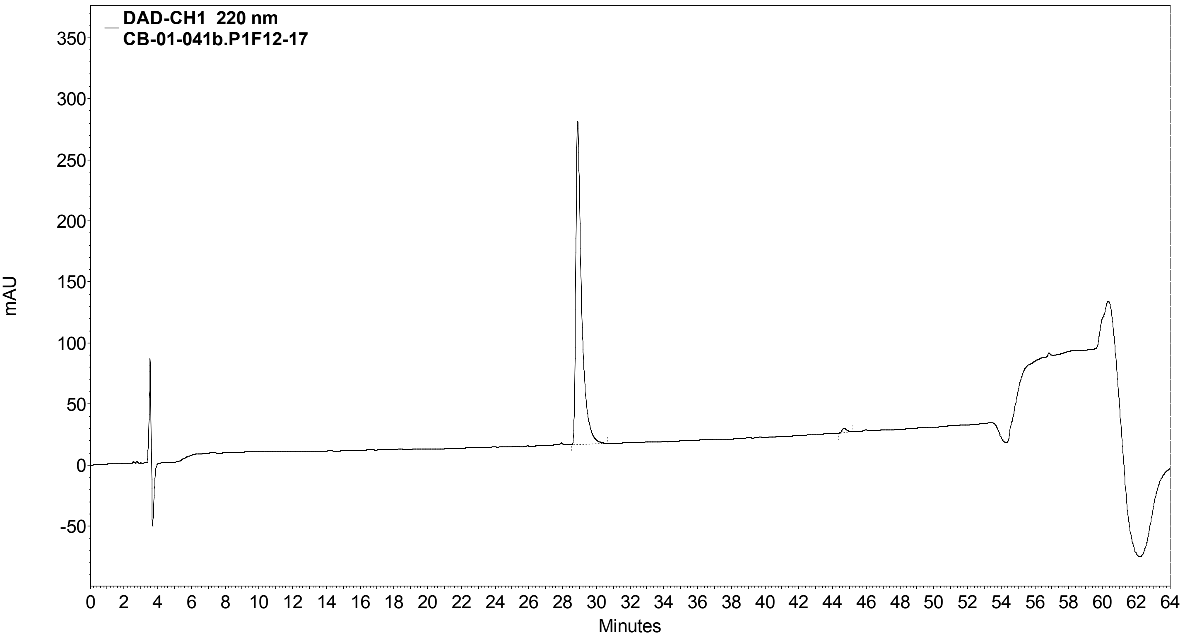


10 % solvent B to 60 % solvent B in 50 min, the compound elutes at 28.90 min. After 50 min, the washing procedure starts.

**Compound 14**

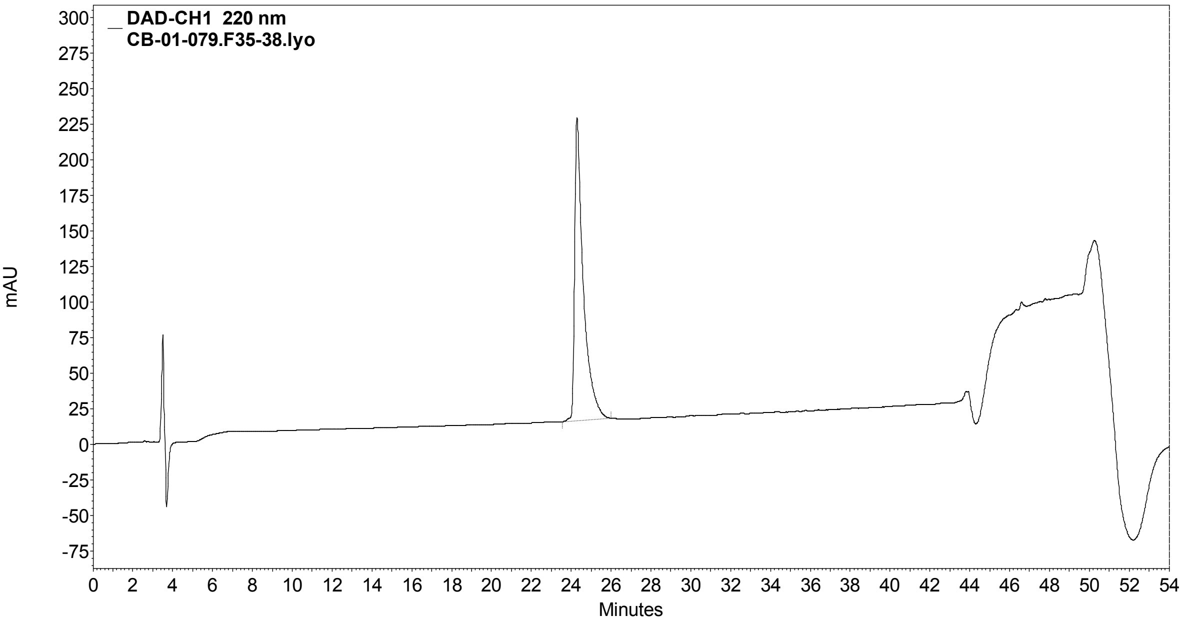


10 % solvent B to 50 % solvent B in 40 min, the compound elutes at 24.30 min. After 40 min, the washing procedure starts.

**Compound 15**

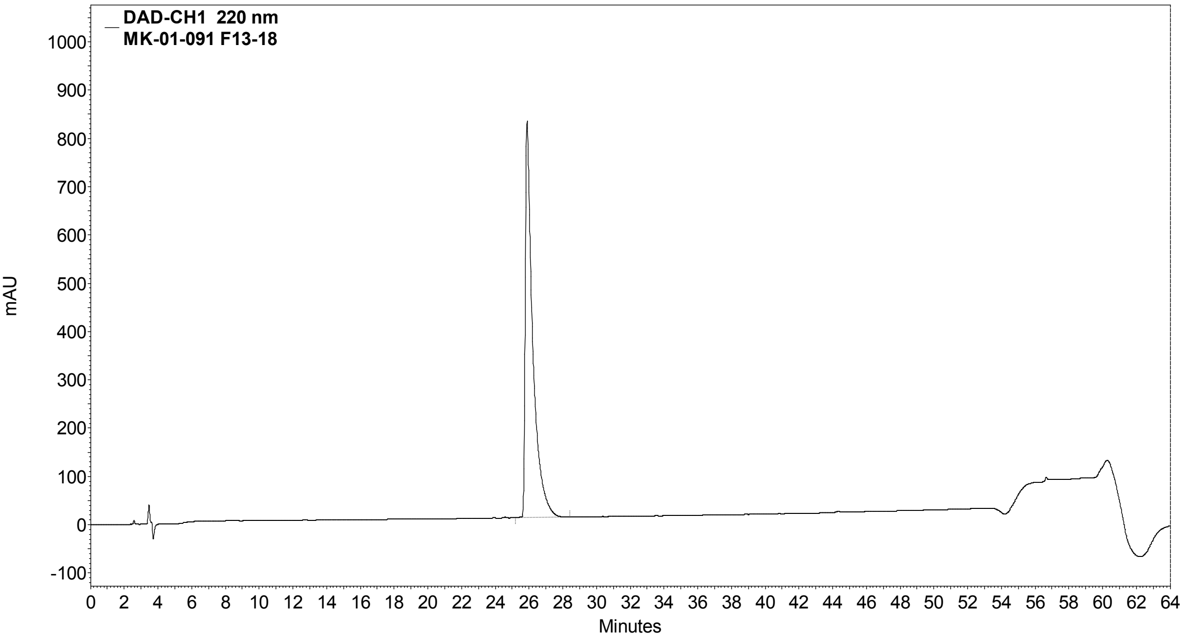


10 % solvent B to 60 % solvent B in 50 min, the compound elutes at 25.89 min. After 50 min, the washing procedure starts.

**Compound 16**

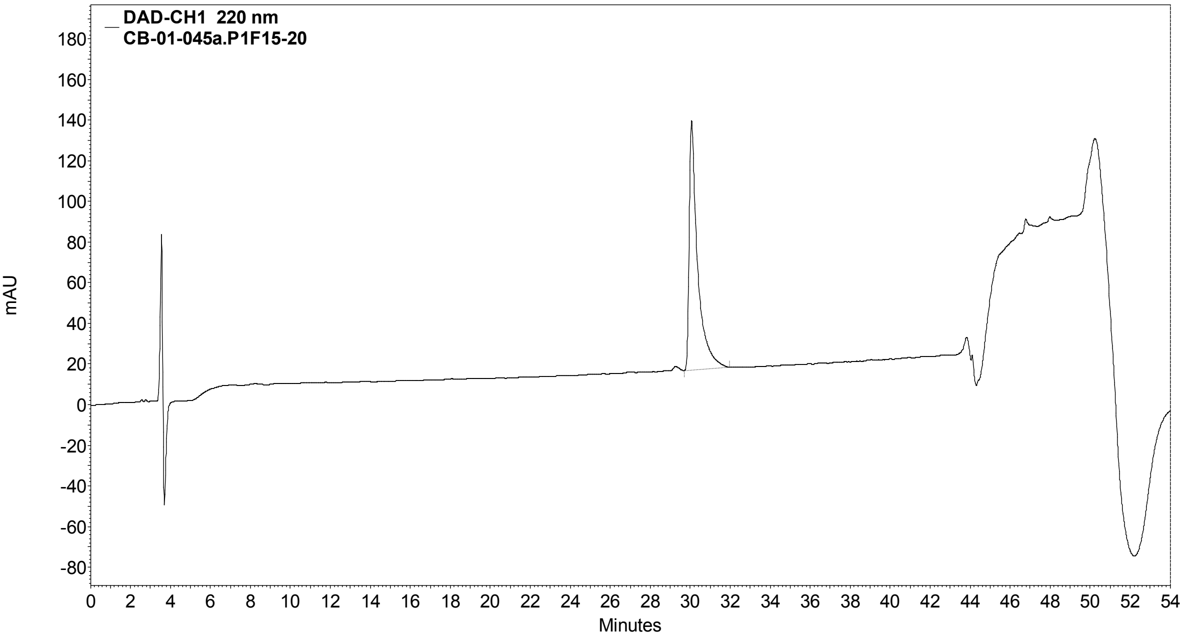


10 % solvent B to 50 % solvent B in 40 min, the compound elutes at 30.07 min. After 40 min, the washing procedure starts.

**Compound 17**

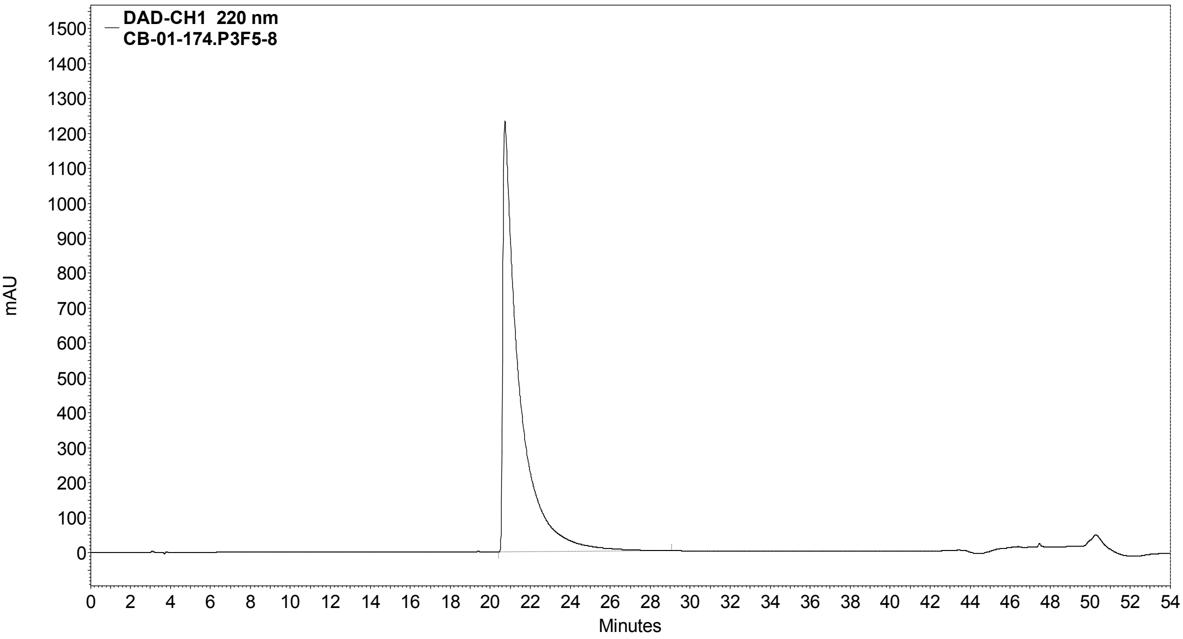


10 % solvent B to 50 % solvent B in 40 min, the compound elutes at 20.73 min. After 40 min, the washing procedure starts.

**Compound 18**

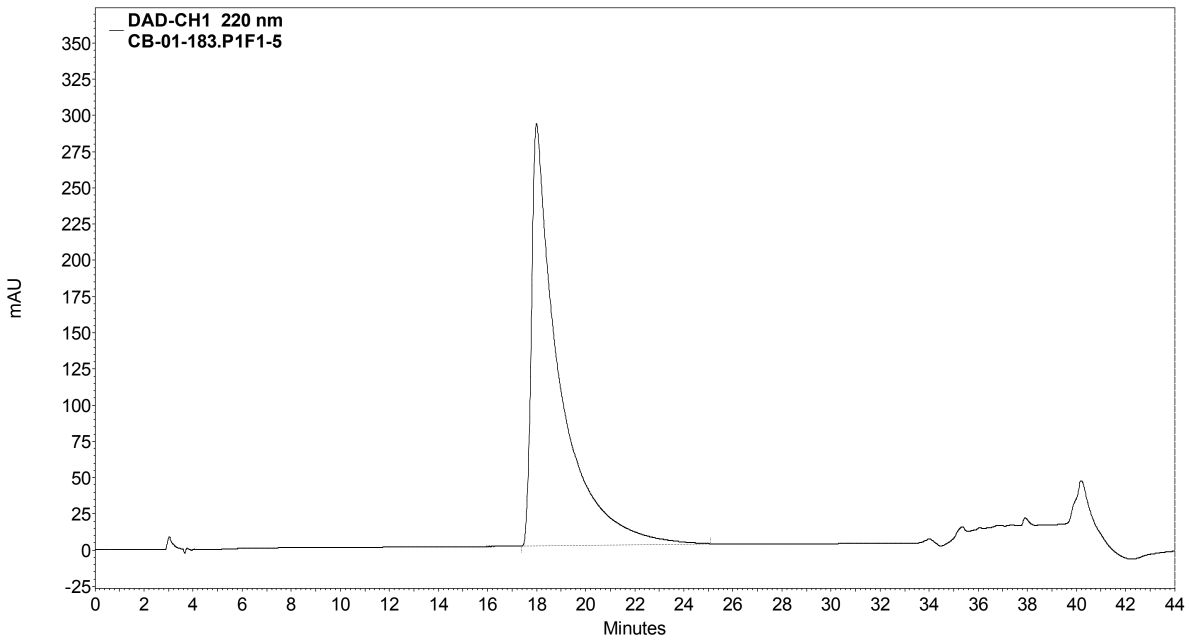


10 % solvent B to 40 % solvent B in 30 min, the compound elutes at 18.00 min. After 30 min, the washing procedure starts.

**Compound 19**

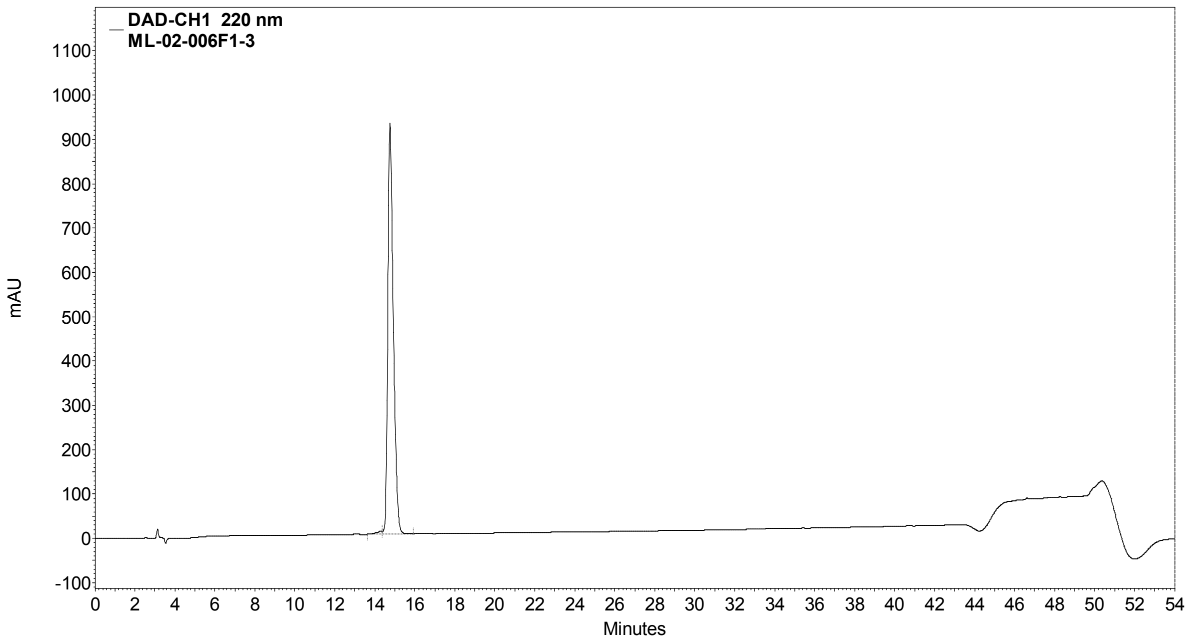


20 % solvent B to 60 % solvent B in 40 min, the compound elutes at 14.75 min. After 40 min, the washing procedure starts.

**Compound 20**

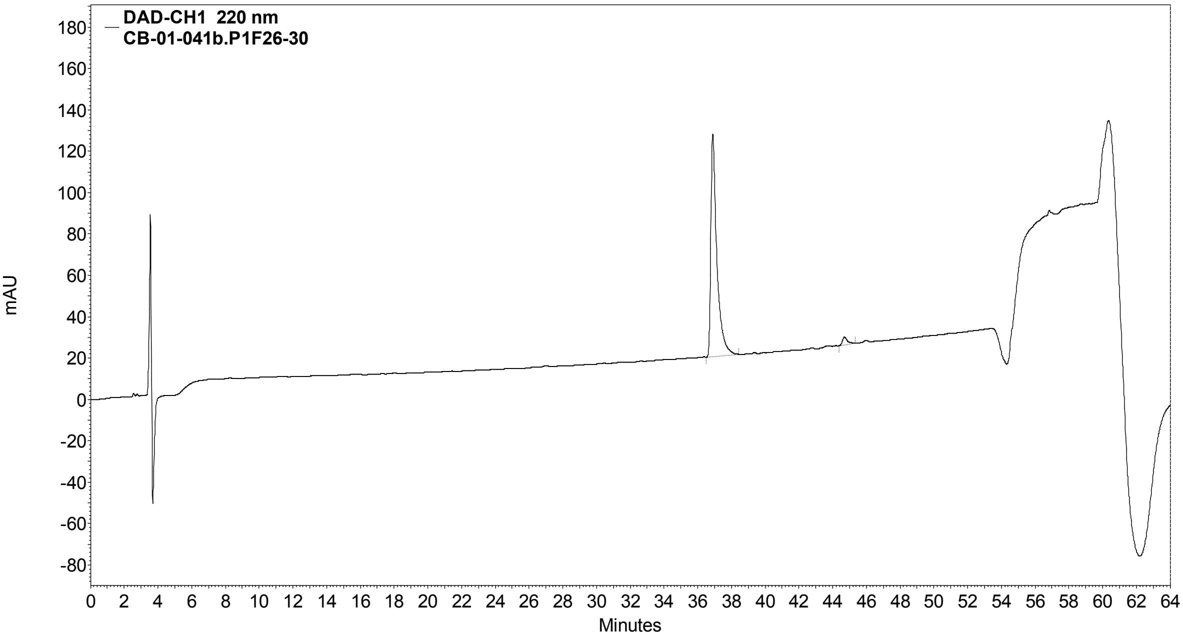


10 % solvent B to 60 % solvent B in 50 min, the compound elutes at 36.89 min. After 50 min, the washing procedure starts.

**Compound 21**

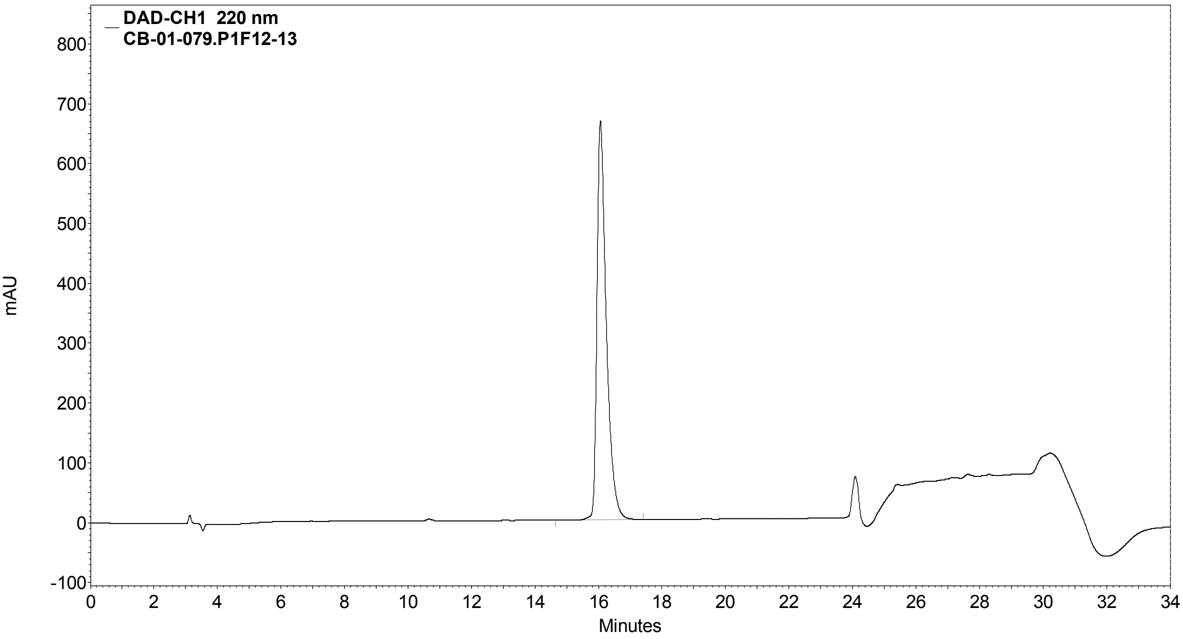


20 % solvent B to 40 % solvent B in 20 min, the compound elutes at 16.06 min. After 20 min, the washing procedure starts.

**Compound 22**

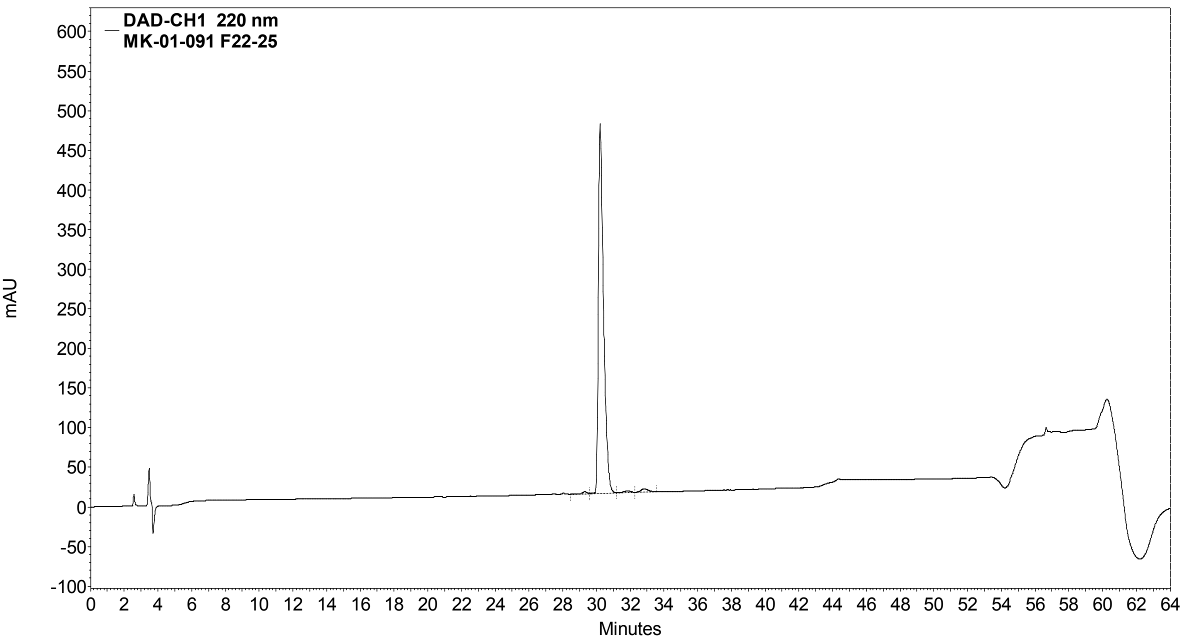


10 % solvent B to 60 % solvent B in 50 min, the compound elutes at 30.22 min. After 50 min, the washing procedure starts.

**Compound 23**

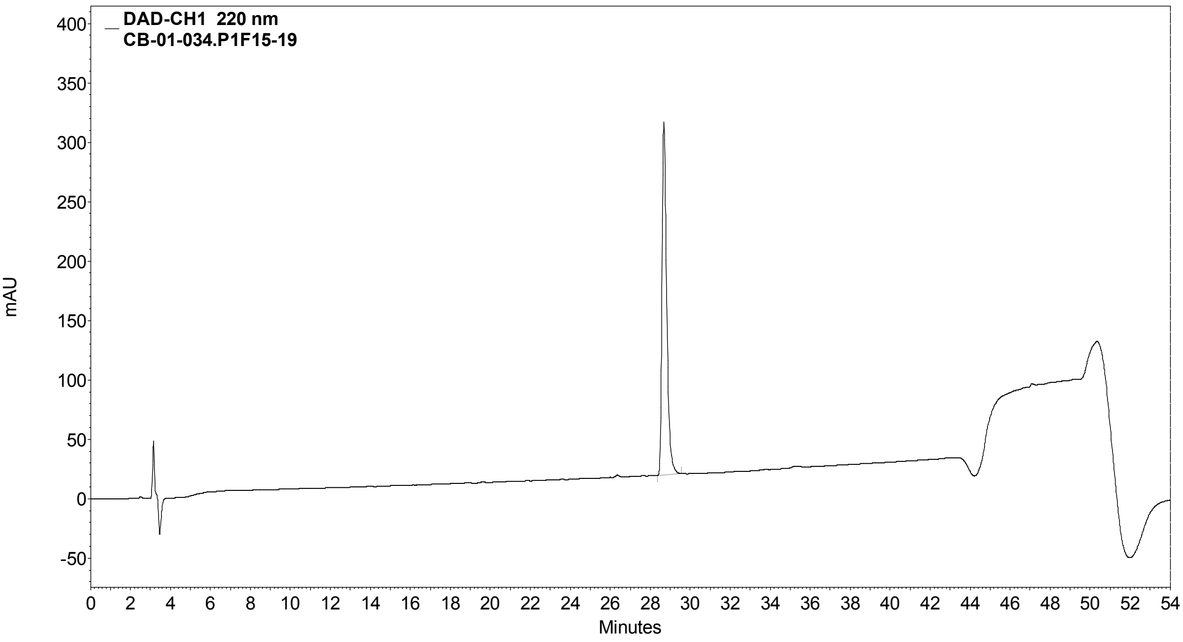


20 % solvent B to 60 % solvent B in 40 min, the compound elutes at 28.69 min. After 40 min, the washing procedure starts.

**Compound 24**

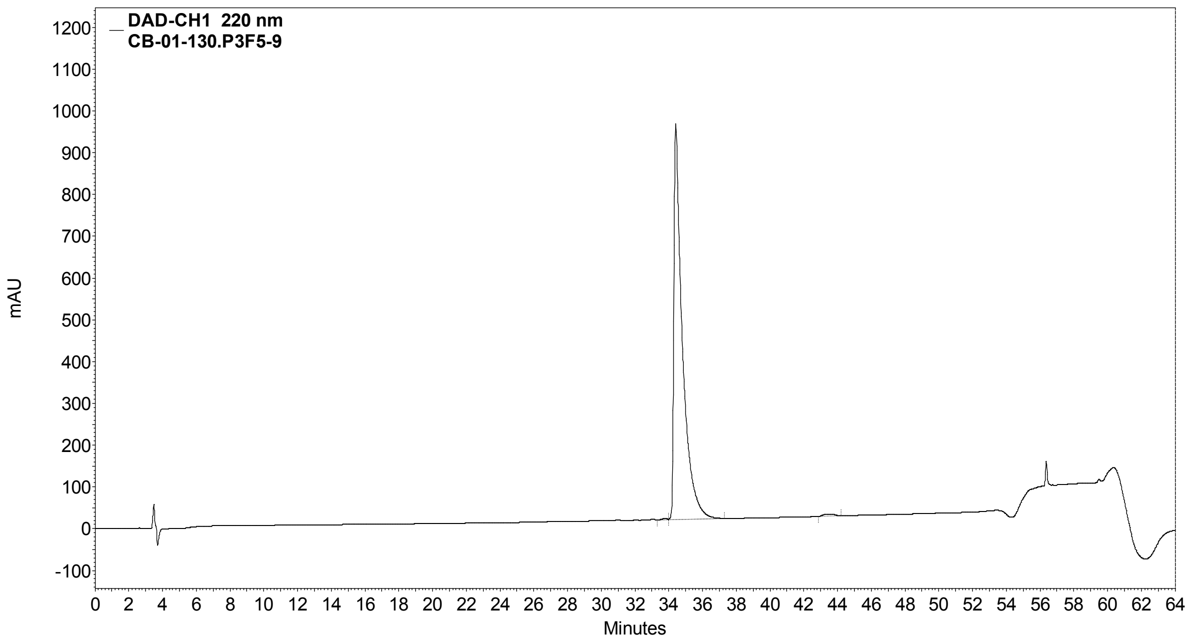


10 % solvent B to 60 % solvent B in 50 min, the compound elutes at 34.41 min. After 50 min, the washing procedure starts.

**Compound 25**

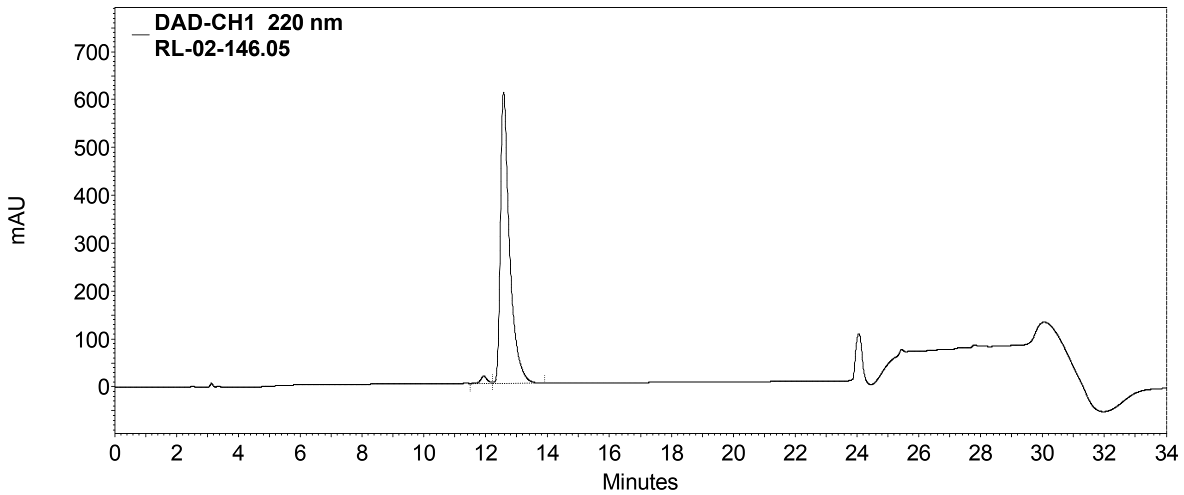


20 % solvent B to 40 % solvent B in 20 min, the compound elutes at 12.57 min. After 20 min, the washing procedure starts.

**Compound 26**

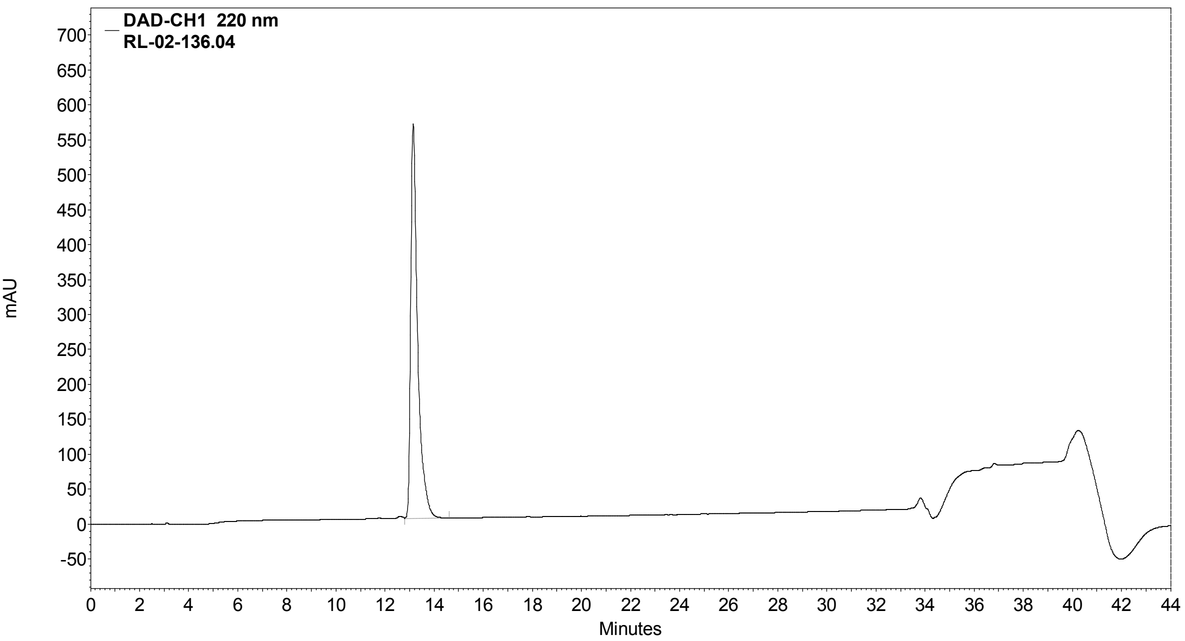


20 % solvent B to 50 % solvent B in 30 min, the compound elutes at 13.15 min. After 30 min, the washing procedure starts.

**Compound 27**

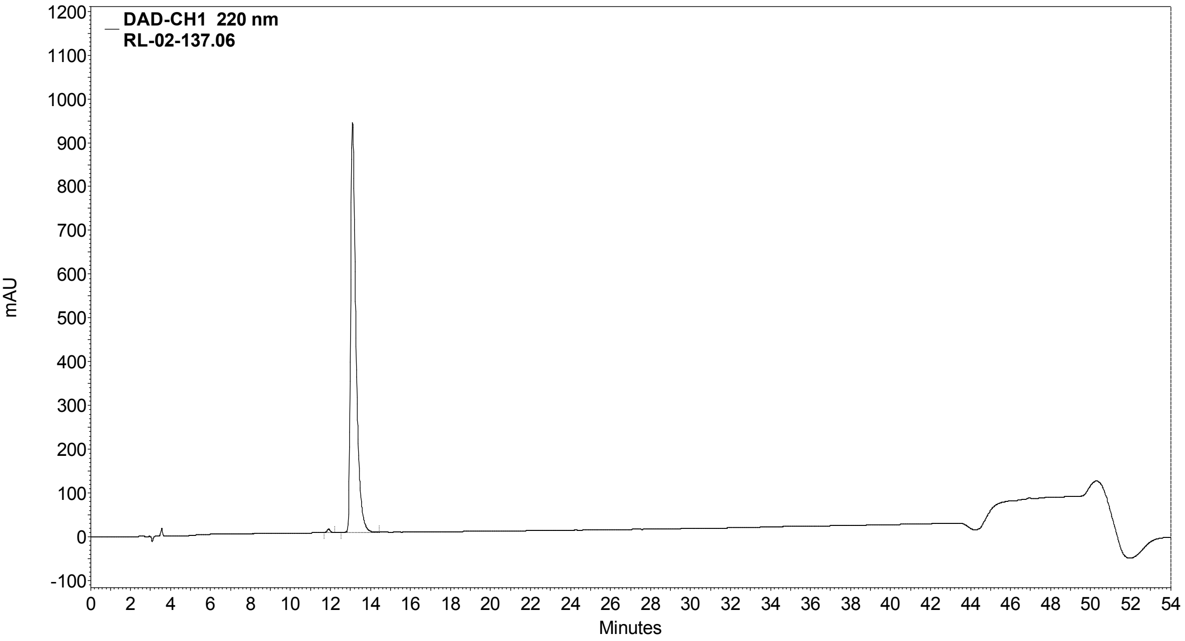


20 % solvent B to 60 % solvent B in 40 min, the compound elutes at 13.10 min. After 40 min, the washing procedure starts.

**Compound 28**

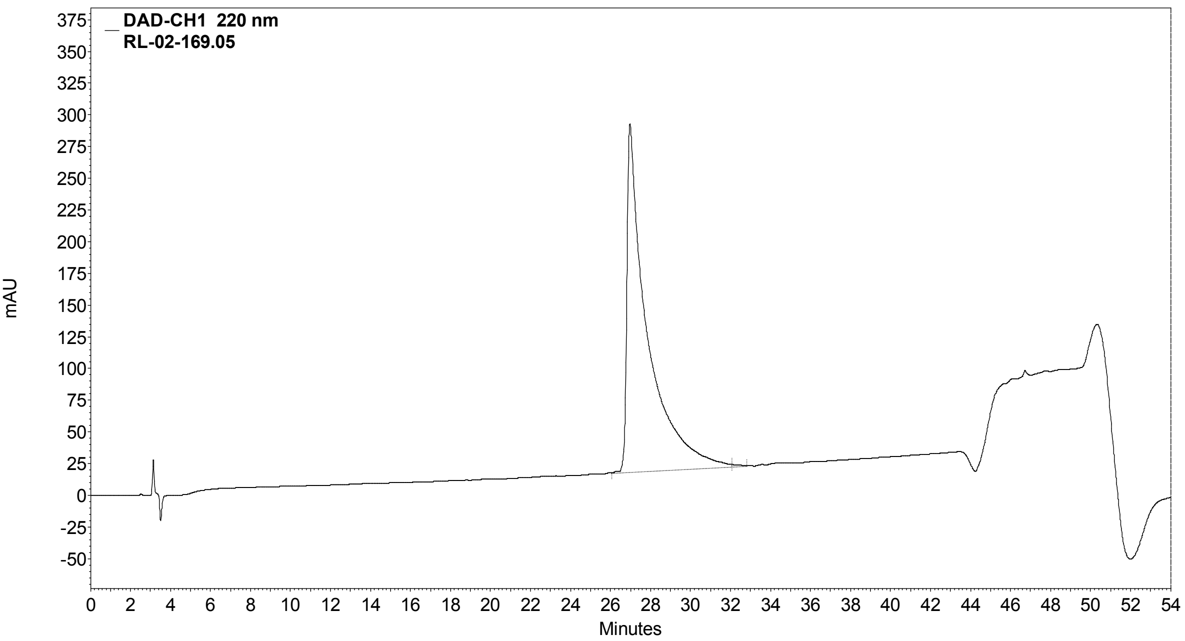


20 % solvent B to 60 % solvent B in 40 min, the compound elutes at 26.97 min. After 40 min, the washing procedure starts.

**Compound 29**

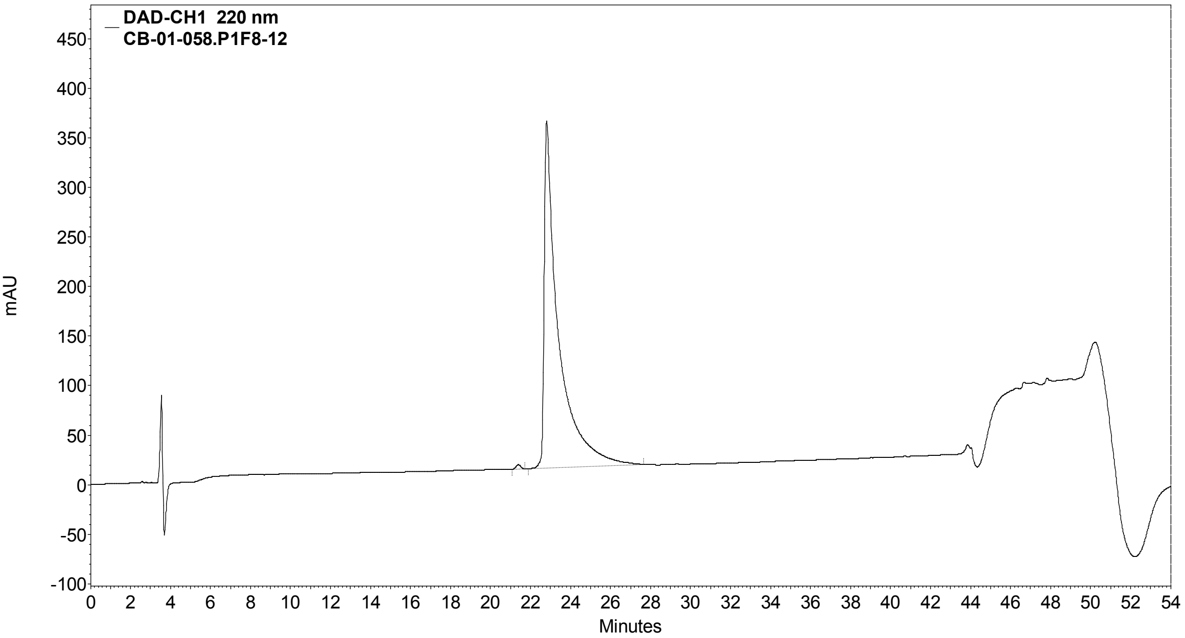


10 % solvent B to 50 % solvent B in 40 min, the compound elutes at 22.81 min. After 40 min, the washing procedure starts.

**Compound 30**

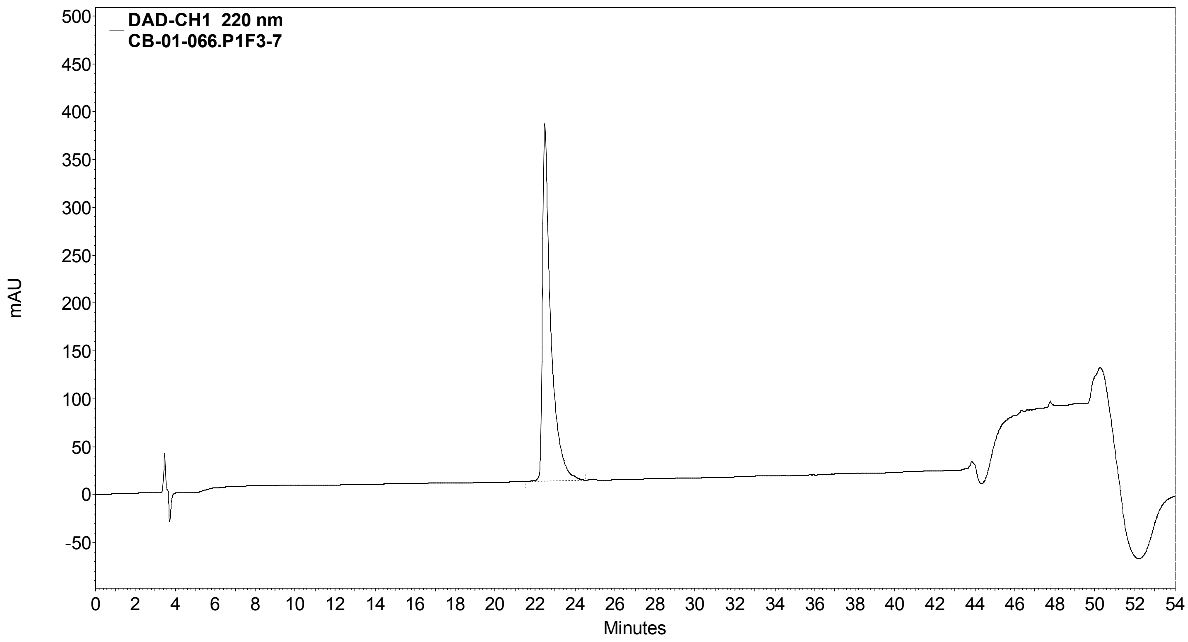


10 % solvent B to 50 % solvent B in 40 min, the compound elutes at 22.49 min. After 40 min, the washing procedure starts.

**Compound 31**

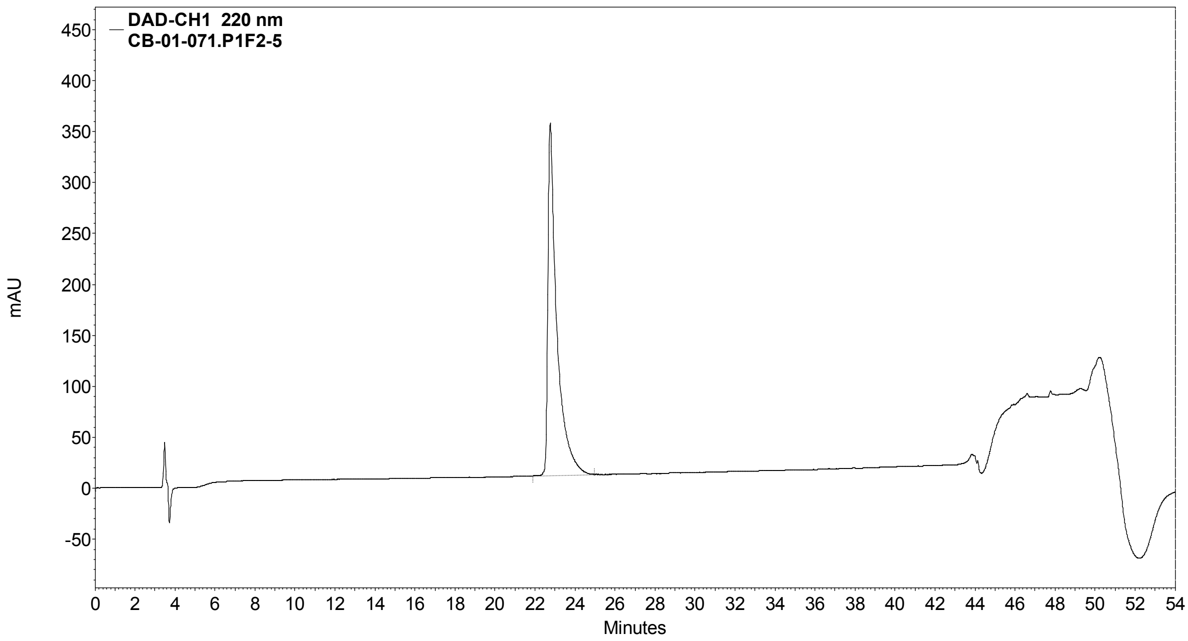


10 % solvent B to 50 % solvent B in 40 min, the compound elutes at 22.77 min. After 40 min, the washing procedure starts.

## Compound 32

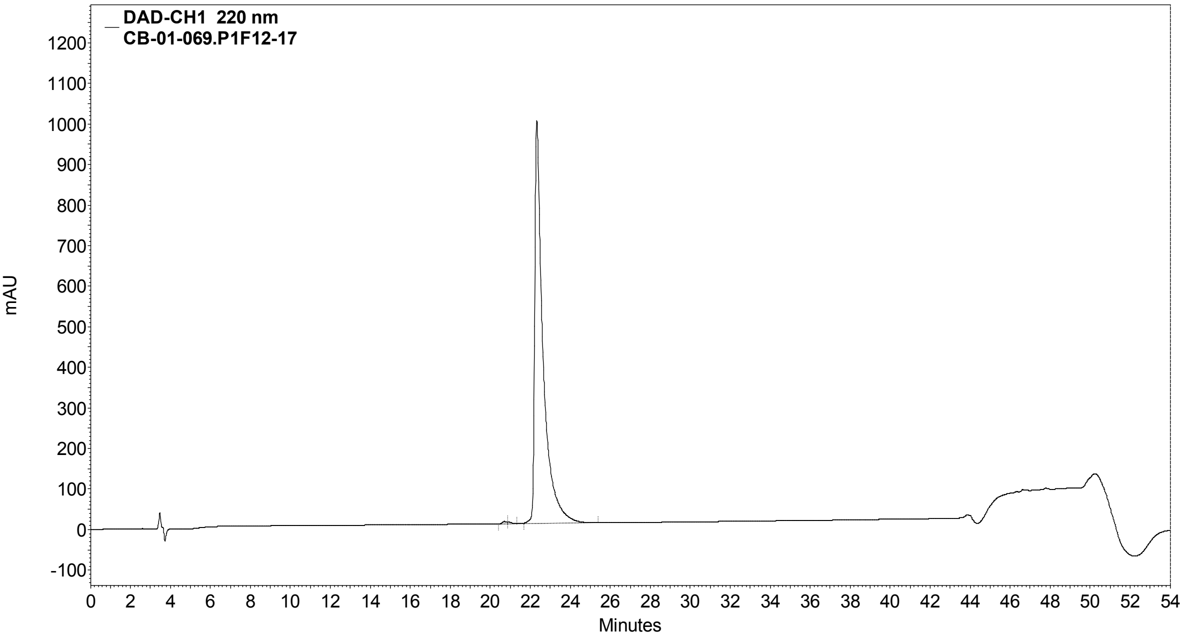


10 % solvent B to 50 % solvent B in 40 min, the compound elutes at 22.33 min. After 40 min, the washing procedure starts.

## Compound 33

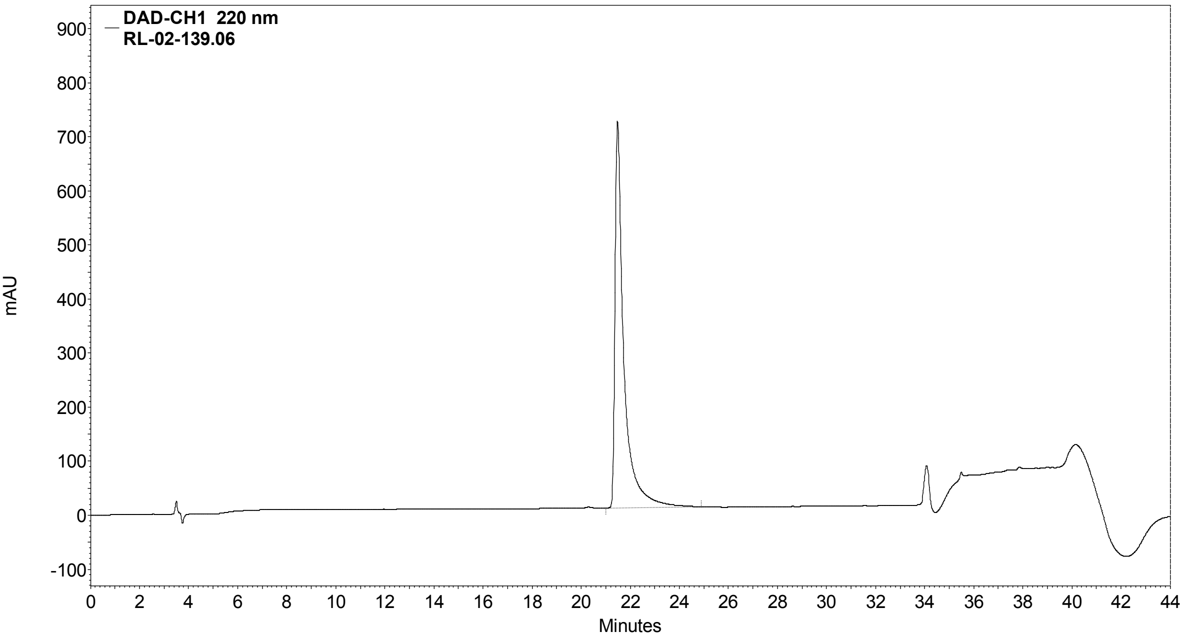


10 % solvent B to 40 % solvent B in 30 min, the compound elutes at 21.48 min. After 30 min, the washing procedure starts.

**Compound 34**

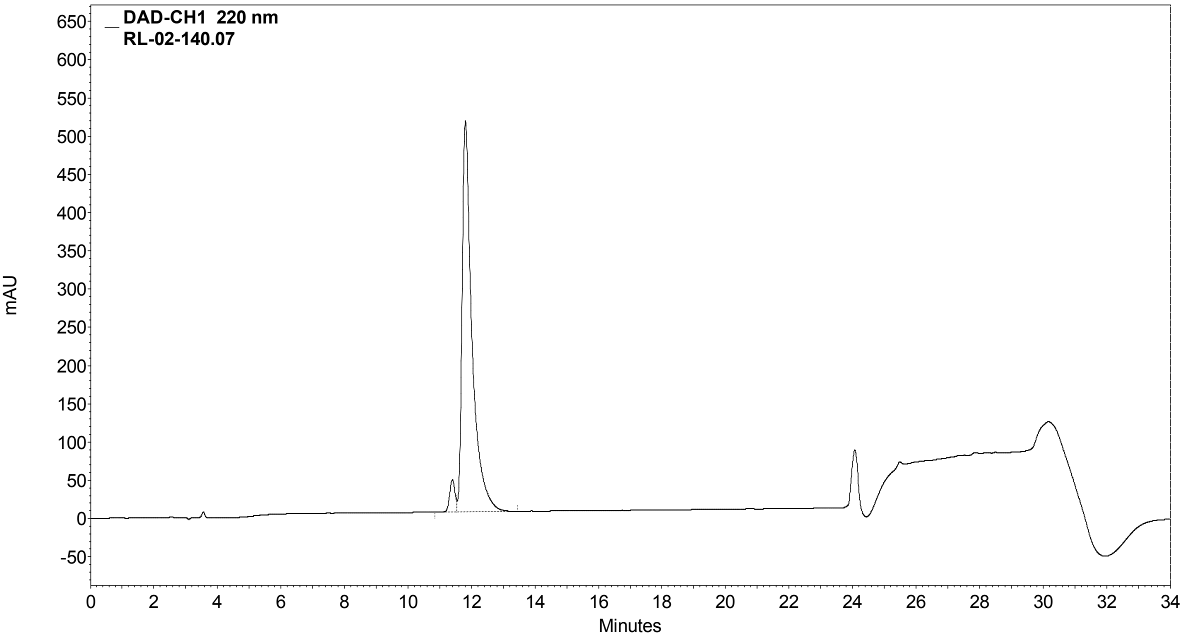


20 % solvent B to 40 % solvent B in 20 min, the compound elutes at 11.81 min. After 20 min, the washing procedure starts.

**Compound 35**

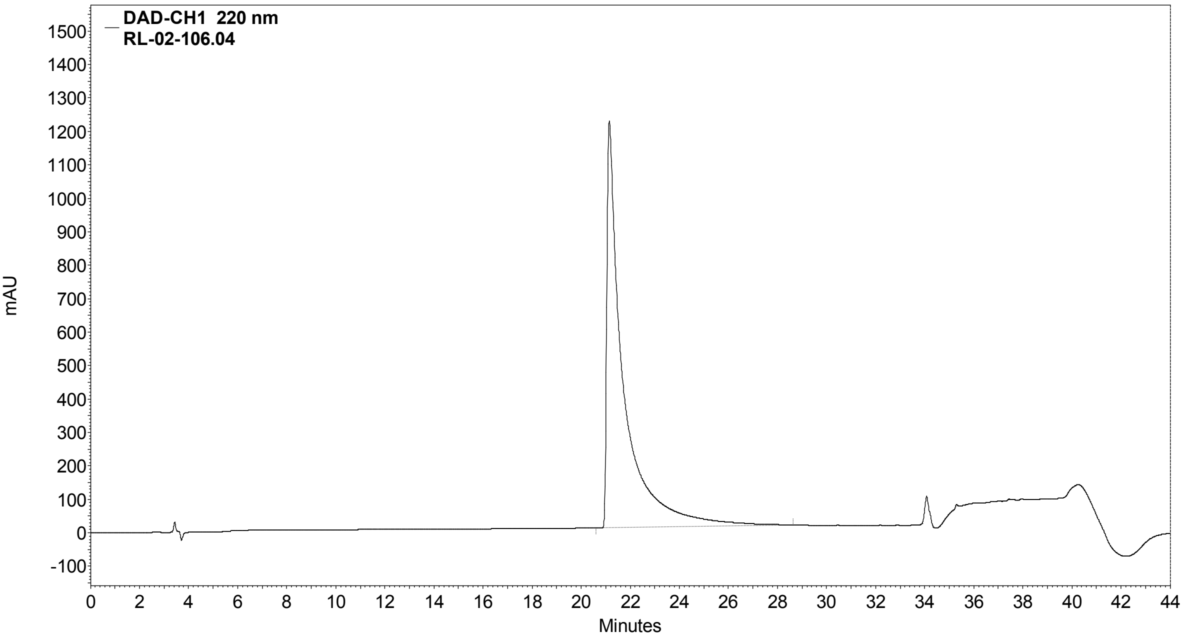


10 % solvent B to 40 % solvent B in 30 min, the compound elutes at 21.15 min. After 30 min, the washing procedure starts.

**Compound 36**

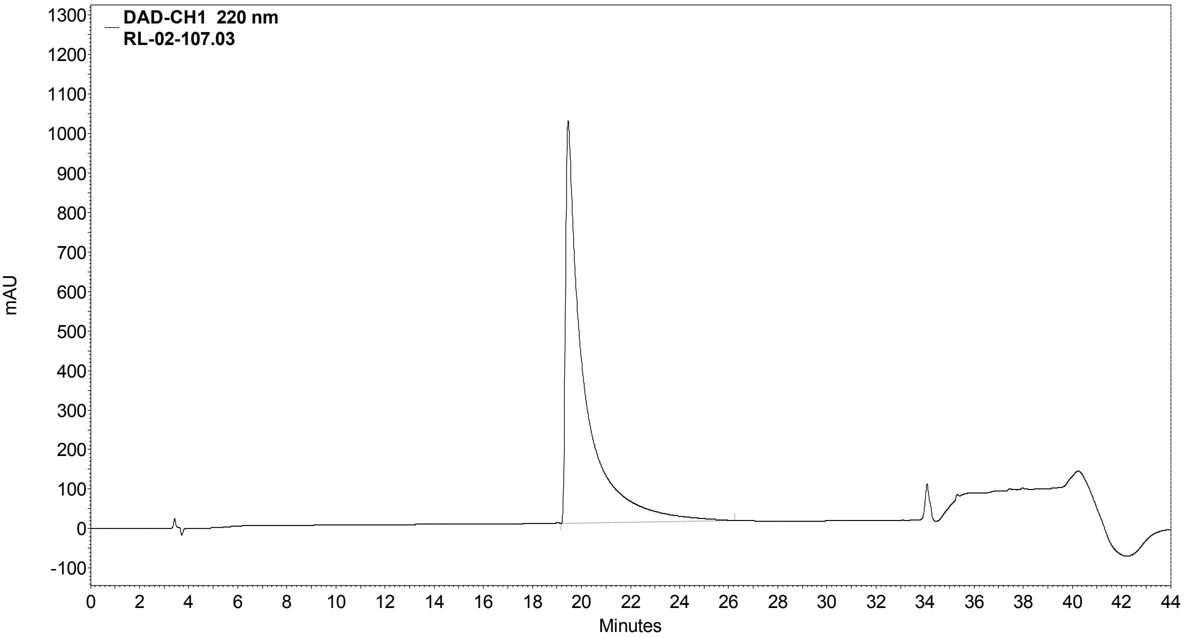


10 % solvent B to 40 % solvent B in 30 min, the compound elutes at 19.46 min. After 30 min, the washing procedure starts.

**Compound 37**

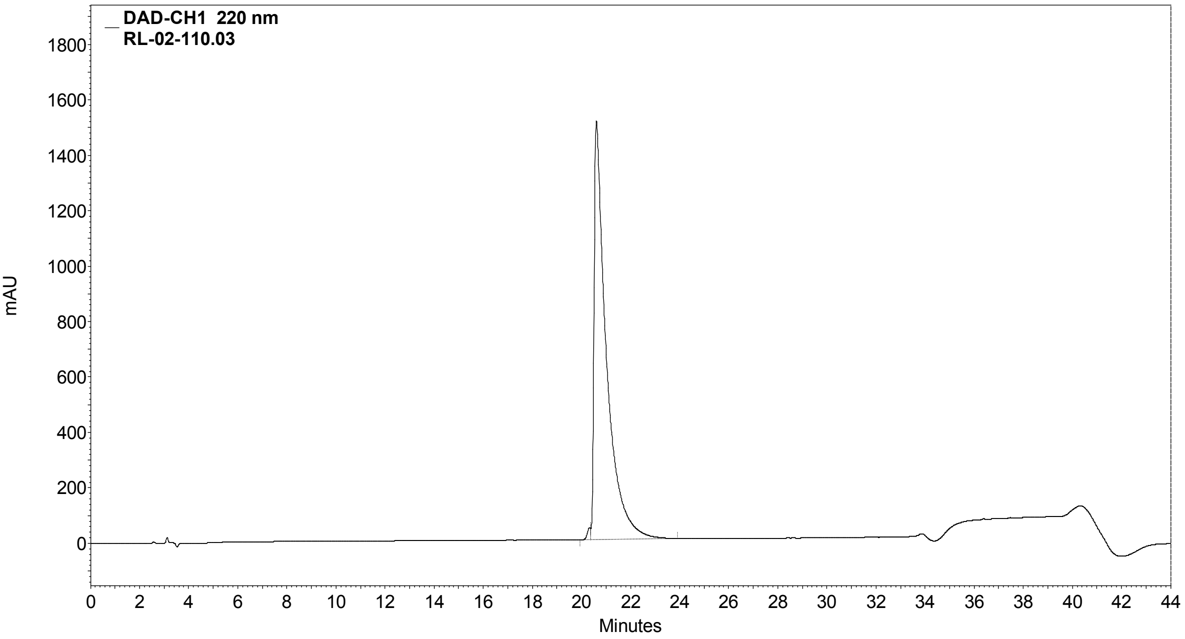


20 % solvent B to 50 % solvent B in 30 min, the compound elutes at 20.61 min. After 30 min, the washing procedure starts.

**Compound 38**

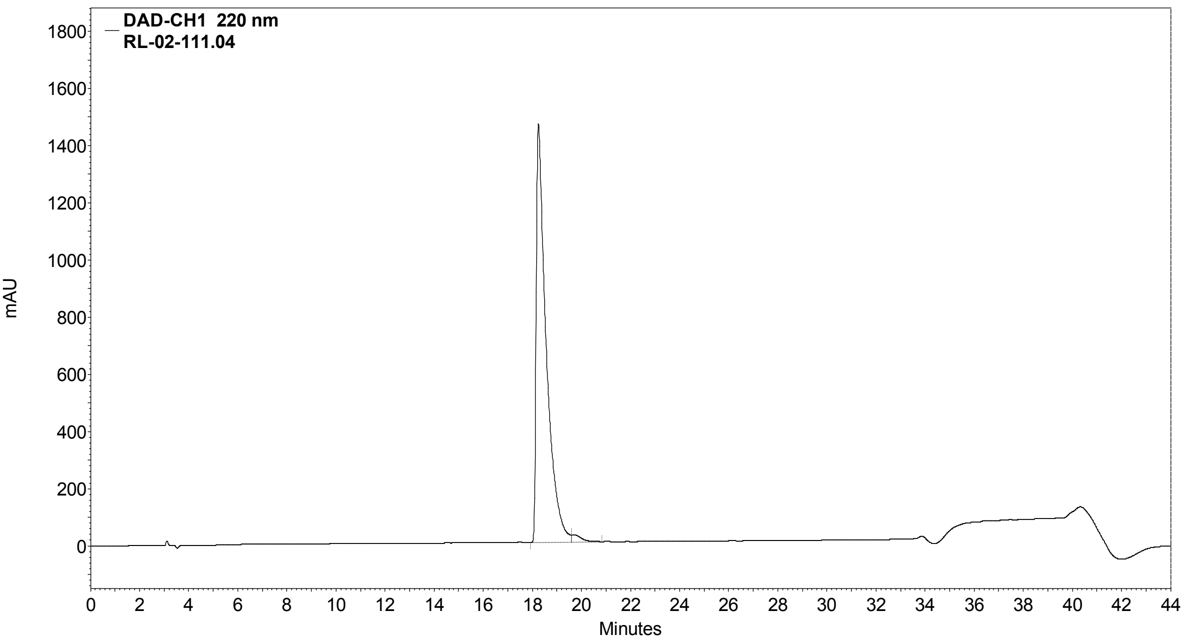


20 % solvent B to 50 % solvent B in 30 min, the compound elutes at 18.25 min. After 30 min, the washing procedure starts.

**Compound 39**

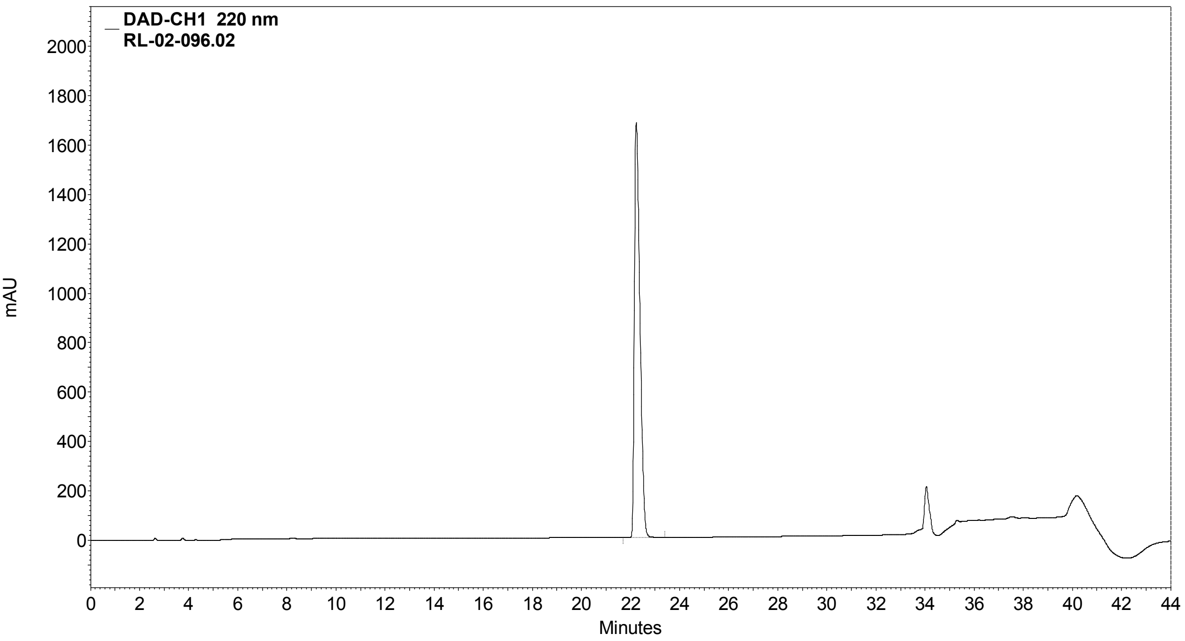


10 % solvent B to 40 % solvent B in 30 min, the compound elutes at 22.23 min. After 30 min, the washing procedure starts.

**Compound 40**

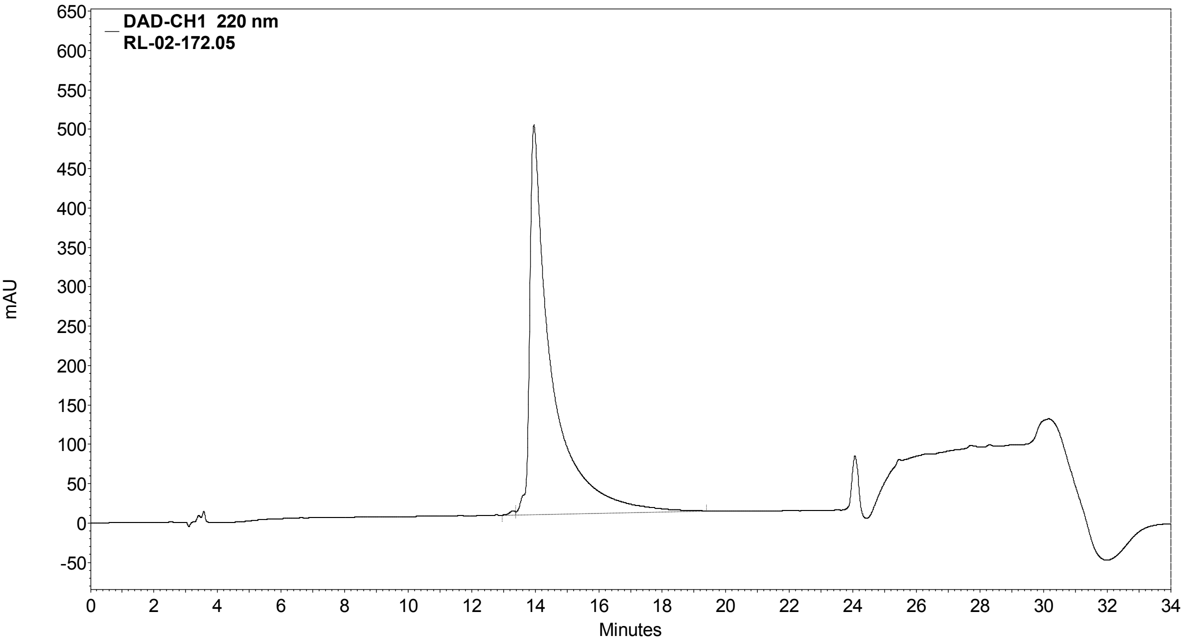


20 % solvent B to 40 % solvent B in 20 min, the compound elutes at 13.96 min. After 20 min, the washing procedure starts.

**Compound 41**

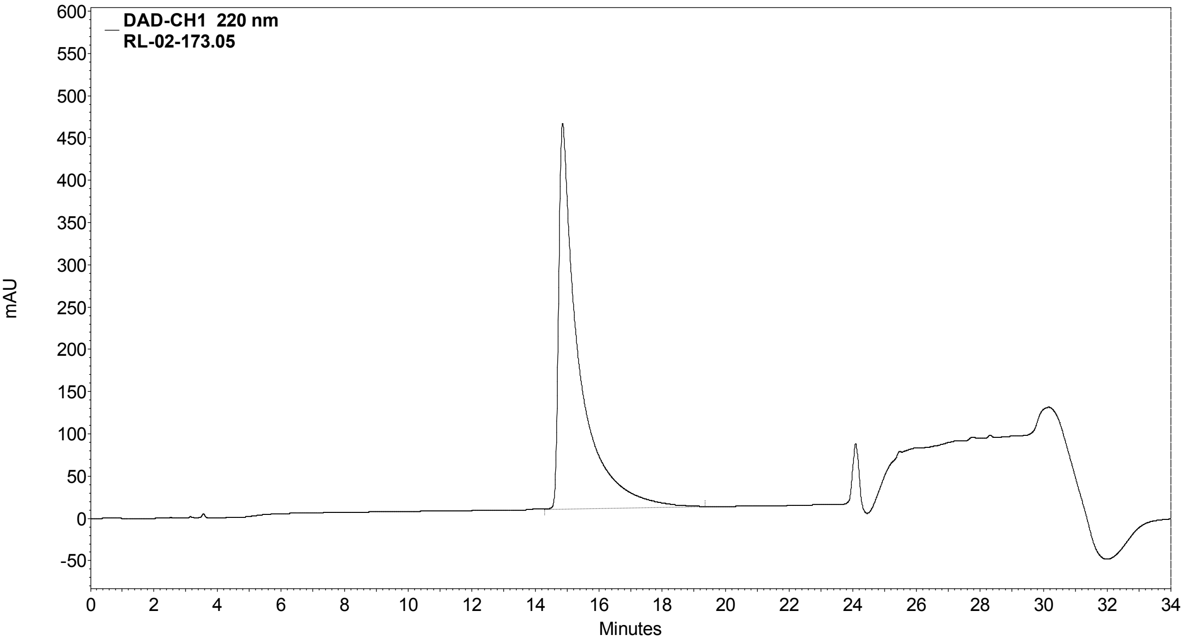


20 % solvent B to 40 % solvent B in 20 min, the compound elutes at 14.86 min. After 20 min, the washing procedure starts.

**Compound 42**

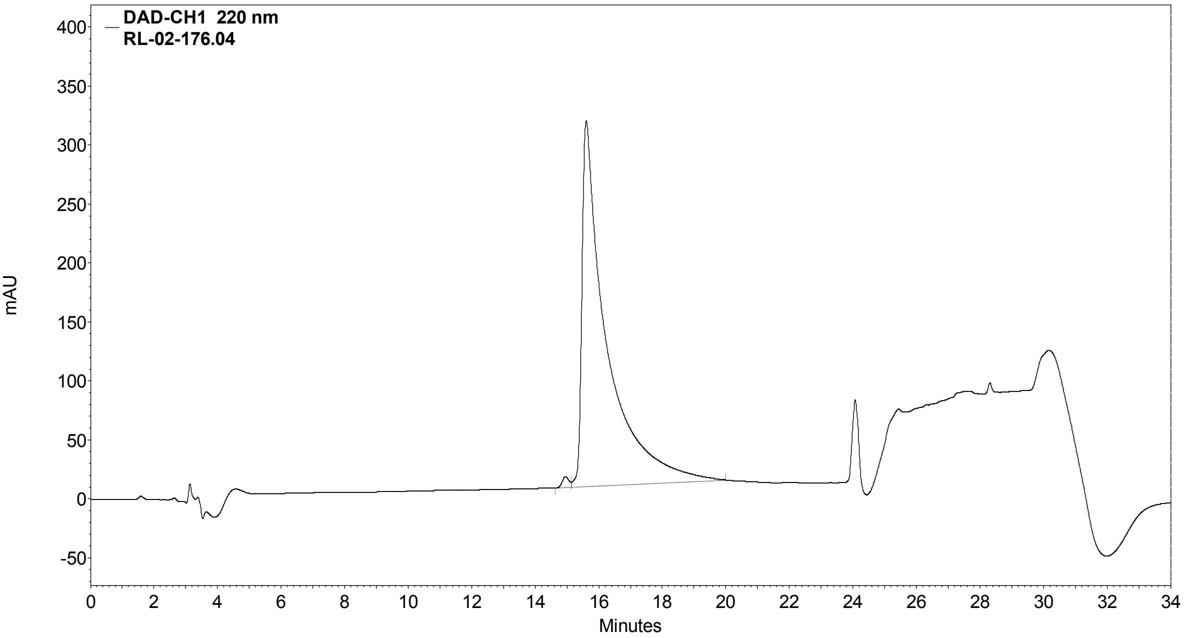


20 % solvent B to 40 % solvent B in 20 min, the compound elutes at 15.61 min. After 20 min, the washing procedure starts.

**Compound 43**

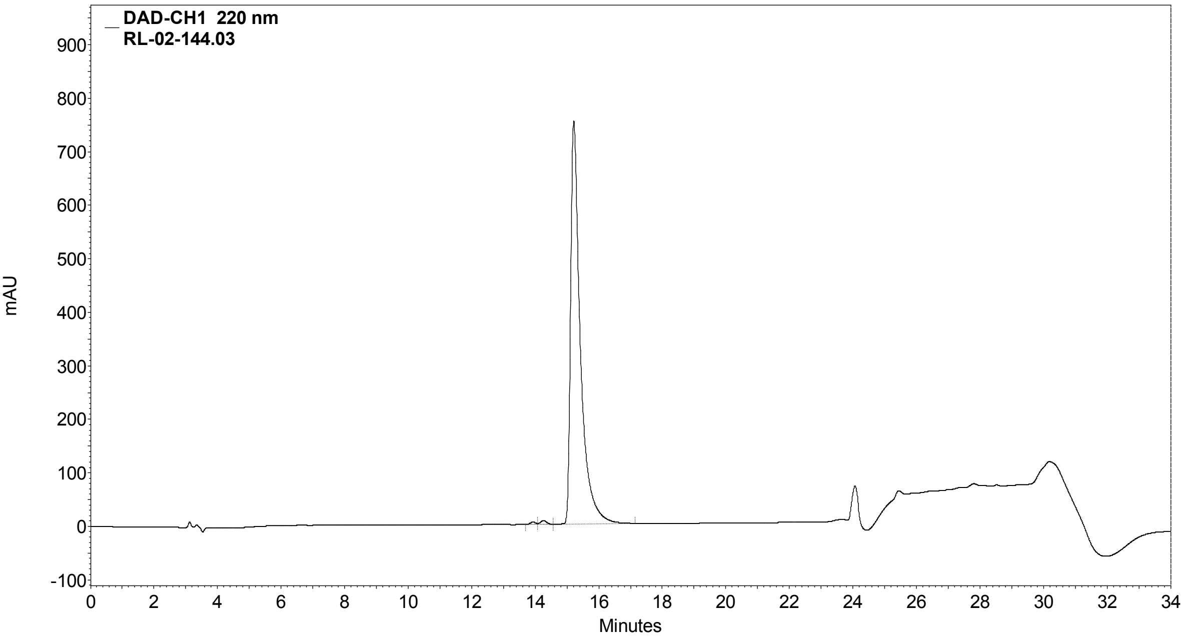


20 % solvent B to 40 % solvent B in 20 min, the compound elutes at 15.21 min. After 20 min, the washing procedure starts.

**Compound 44**

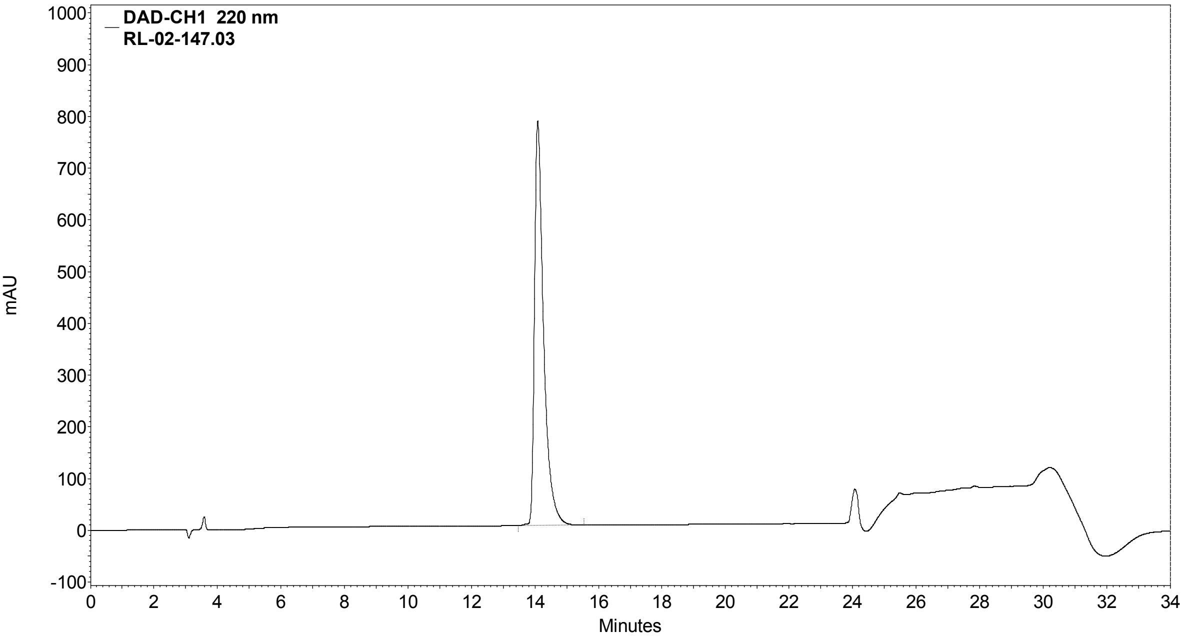


20 % solvent B to 40 % solvent B in 20 min, the compound elutes at 14.01 min. After 20 min, the washing procedure starts.

**Compound 45**

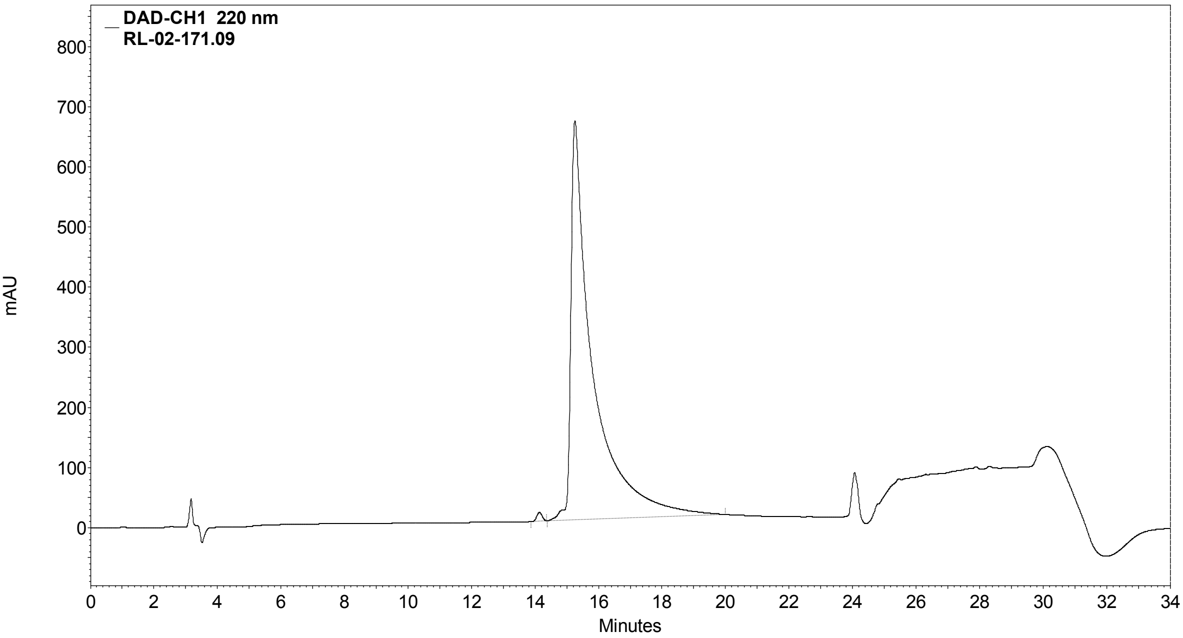


20 % solvent B to 40 % solvent B in 20 min, the compound elutes at 15.26 min. After 20 min, the washing procedure starts.

**Compound 46**

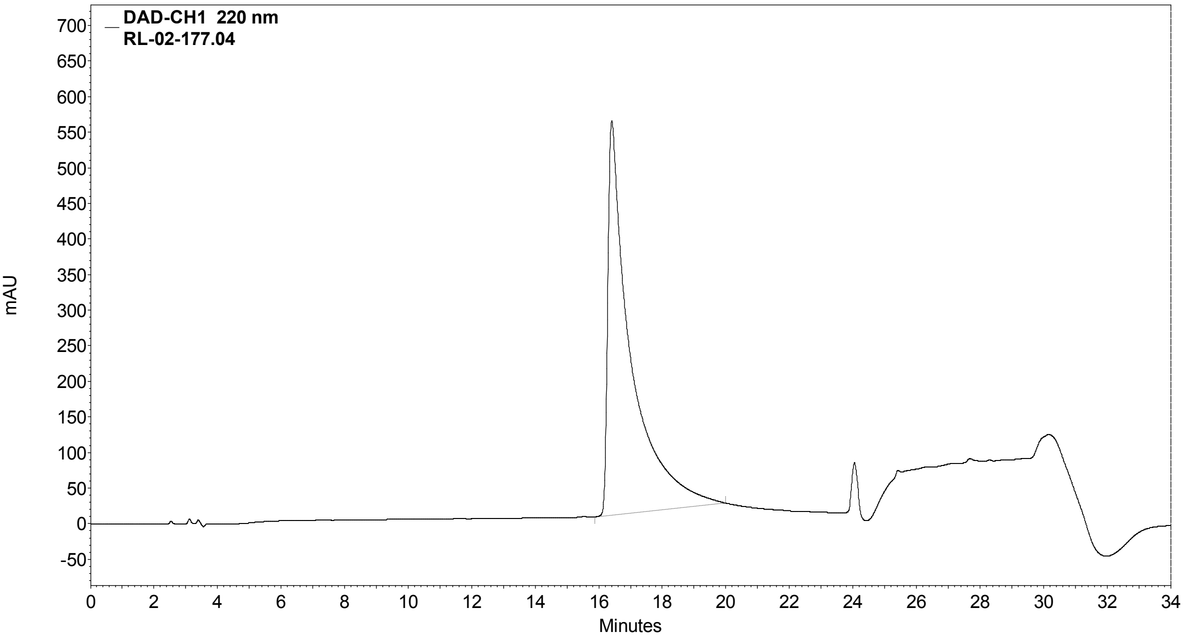


20 % solvent B to 40 % solvent B in 20 min, the compound elutes at 16.41 min. After 20 min, the washing procedure starts.

## 5. Structure determination of furin in complex with inhibitors 13, 24, 26, 27, 34, and 41

Table S1. Data collection and refinement statistics.

| **Data collection statistics** | **13 (MI-3102)** | **24 (MI-3140)** | **26 (MI-2456)** | **27 (MI-2471)** | **34 (MI-2470)** | **41 (MI-2464)** |
|---|---|---|---|---|---|---|
| PDB ID | 9QWF | 9QWG | 9QWB | 9QWE | 9QWD | 9QWC |
| Soaking concentration (mM) | 5 | 5 | 5 | 5 | 5 | 5 |
| Beamline | ESRF ID30A-3 | ESRF ID30A-3 | ESRF ID30A-3 | ESRF ID30A-3 | ESRF ID30A-3 | ESRF ID30A-3 |
| Wavelength (Å) | 0.967697 | 0.967697 | 0.967697 | 0.967697 | 0.967697 | 0.967697 |
| Space group | $P6_522$ | $P6_522$ | $P6_522$ | $P6_522$ | $P6_522$ | $P6_522$ |
| Unit cell parameters (Å): a = b, c | 131.3, 155.2 | 131.2, 154.8 | 131.0, 155.3 | 131.1, 155.1 | 131.5, 155.4 | 131.2, 155.4 |
| Resolution range [a] (Å) | 43.0-1.65 (1.75-1.65) | 45.8-1.6 (1.70-1.60) | 42.9-1.6 (1.70-1.60) | 42.9-1.6 (1.70-1.60) | 45.9-1.6 (1.70-1.60) | 45.9-1.5 (1.59-1.50) |
| $R_{meas}$ [a] (%) | 14.8 (230.5) | 27.6 (404.1) | 11.1 (199.7) | 27.4 (481.2) | 13.0 (193.1) | 11.4 (185.4) |
| I/sigI [a] | 16.3 (1.5) | 12.1 (1.3) | 20.3 (1.8) | 13.2 (1.2) | 16.2 (1.8) | 19.4 (1.9) |
| $CC_{1/2}$ (%) [a] | 99.9 (68.8) | 99.8 (49.0) | 100.0 (73.6) | 99.8 (42.5) | 99.9 (81.1) | 99.9 (76.6) |
| Completeness [a] | 97.5 (97.2) | 95.7 (89.7) | 96.8 (96.2) | 97.1 / 92.9 | 99.9 (99.9) | 96.3 (94.8) |
| No. of observations (total/unique) | 1961950 / 92367 | 2115290 / 98793 | 2140367 / 100122 | 2111914 / 103405 | 2154743 / 104095 | 2595113 / 121051 |
| **Refinement statistics** | | | | | | |
| No. of non-hydrogen atoms | 4338 | 4311 | 4394 | 4358 | 4359 | 4365 |
| Protein / inhibitor / water / other | 3776 / 36 / 476 / 50 | 3759 / 38 | 3801 / 66 / 477 / 50 | 3766 / 78 / 464 / 50 | 3780 / 80 / 449 / 50 | 3800 / 43 / 472 / 50 |
| $R_{work}/R_{free}$ | 15.5 / 16.8 | 14.8 / 18.3 | 13.1 / 15.8 | 15.1 / 18.5 | 13.5 / 16.1 | 12.9 / 14.8 |
| **B-factors (Å²)** | | | | | | |
| Overall/Wilson plot | 26.2 / 29.2 | 21.2 / 25.3 | 27.1 / 29.2 | 23.1 / 27.5 | 25.1 / 28.2 | 23.3 / 25.7 |
| Protein / inhibitor / water / other | 25.0 / 25.1 / 34.9 / 38.9 | 19.6 / 18.0 / 32.8 / 35.9 | 25.2 / 24.0 / 40.5 / 47.0 | 21.4 / 22.7 / 34.7 / 39.1 | 23.4 / 28.6 / 37.0 / 42.7 | 21.6 / 20.9 / 35.7 / 39.9 |
| RMSD bond length (Å) | 0.008 | 0.008 | 0.007 | 0.008 | 0.007 | 0.007 |
| RMSD bonded B-factors (Å²) | 3.3 | 1.8 | 1.9 | 2.0 | 1.8 | 1.7 |

[a] Values of the highest resolution shell are given in parentheses

## 6. Hydrophobic binding pocket for the central dichloro-biphenyl segment

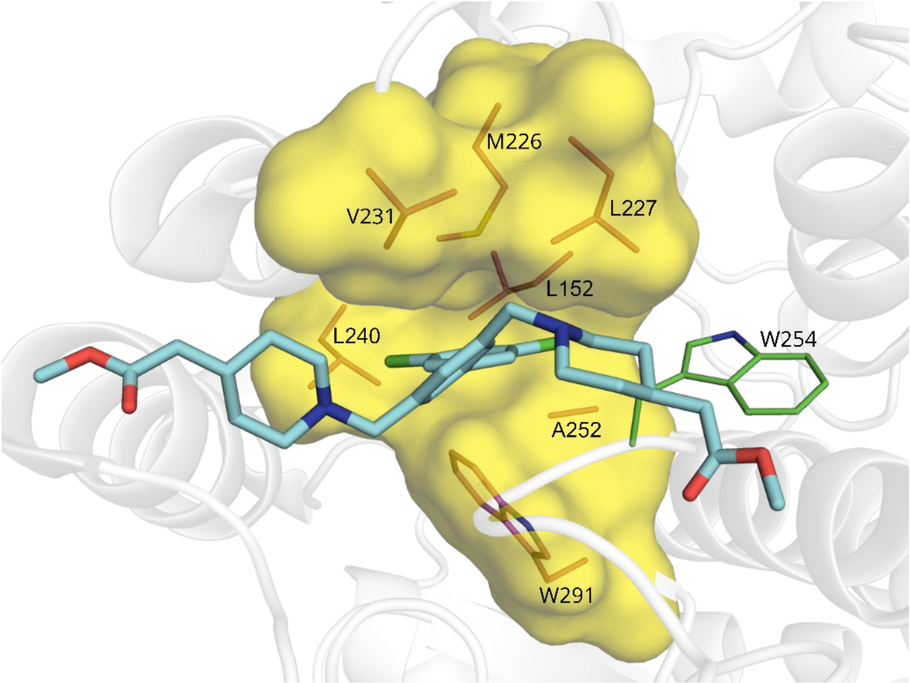


**Figure S2.** Structure of furin in complex with inhibitor **24**. The cryptic binding pocket of furin is opened after a rotation of the Trp254 side chain (green carbon atoms) by approximately 180° and surrounded by the hydrophobic side chains of furin residues Leu152, Met226, Leu227, Val231, Leu240, Ala252 and Trp291 shown with carbon atoms in magenta and a surface in yellow. In case of residues Met226 and Leu227, two conformations have been observed. For clarity, only one conformation of these two residues is shown here.

**7. Structures of furin in complex with the inhibitors**

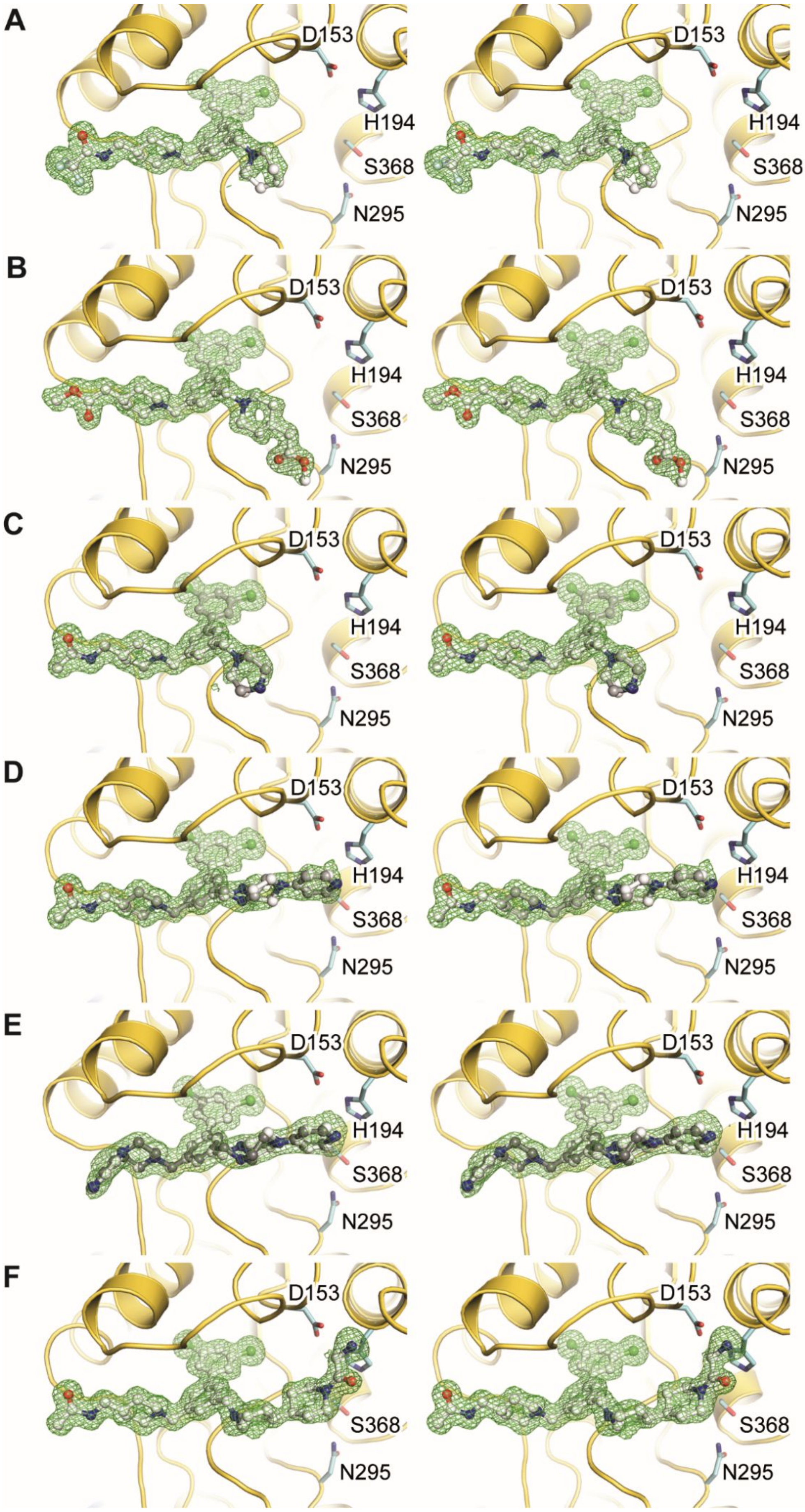

**Figure S3**. Structures of furin bound to inhibitors in stereo representation. The protease is shown as cartoon representation (yellow), catalytic residues as sticks with carbon atoms in cyan and the inhibitors as ball-and-stick models (gray). Different inhibitor conformations are shown with dark gray carbons. The $\mathbf{F_o}$-$\mathbf{F_c}$ annealed omit electron density map of the inhibitors is shown as green mesh and is contoured at 3.0 σ. (A) complex with inhibitor **13**, (B) **24**, (C) **26**, (D) **27** (E) **34** (F) **41**.

## 8. Determination of antiviral activities against the HPAIV strain SC35M

The experiments have been performed as described previously.[3]

**Influenza A SC35M infections and inhibition of multicycle replication:** SC35M infection experiments were carried out in Dulbecco's Modified Eagle Medium (DMEM, Gibco, Thermo Fisher Scientific, Paisley, UK) supplemented with 0.1 % Bovine Serum Albumin (BSA, Bovine Serum Albumin solution 30 % in saline, IgG free, SIGMA-ALDRICH Co.), penicillin, streptomycin (Pen Strep, Gibco, Thermo Fisher Scientific, Paisley, UK) and 2 mM L-Glutamine (L-Glutamine 200 mM, Gibco, Thermo Fisher Scientific, Paisley, UK). Recombinant influenza A virus SC35M/H7N7 (reverse genetics system kindly provided by Jürgen Stech (Friedrich Loeffler Institute, Greifswald - Isle of Riems, Germany). SC35M was propagated in MDCKII cells in infection medium (DMEM (Gibco) supplemented with 0.1 % bovine serum albumin (BSA), glutamine, and antibiotics). Cell supernatants were cleared from cell debris by low-speed centrifugation and stored as virus stocks at -80 °C. For inhibition of SC35M multicycle replication, adenocarcinomic human alveolar basal epithelial cells A549 were grown in DMEM supplemented with 10 % fetal bovine serum (FBS, Gibco, Thermo Fisher Scientific, Paisley, UK), penicillin, streptomycin and 2 mM L-Glutamine until 95 % confluence. Growth medium was removed, cells were washed with PBS def. and infected with SC35M at a MOI of 0.0001 for 1 h at 37 °C, 5 % $CO_2$. Inoculum was removed, cells were washed with PBS def. and fresh medium with or without inhibitors was added. Cells were then further incubated at 37 °C, 5 % $CO_2$ for 72 h in total. 50 µL cell supernatant was collected at different time points and stored at -20 °C until plaque formation assay titration.

**Plaque assay titration SC35M:** Madin-Darby canine kidney cells II (MDCKII) were cultured until 95 % cell confluence was reached, growth medium was aspirated and cells washed with PBS. Cells were then inoculated with 10-fold serial dilutions of cell supernatants in Dulbecco's Modified Eagle Medium (Gibco) supplemented with 0.1 % Bovine Serum Albumin (BSA), penicillin, streptomycin and 2 mM L-Glutamine and incubated at 37 °C, 5 % $CO_2$ for 1 hour. Inoculum was removed and replaced with 50 % v/v double-concentrated MEM (2xMEM (Gibco) supplemented with 0.6 % BSA solution 30 % in saline, IgG free, SIGMA-ALDRICH Co.), penicillin, streptomycin (Pen Strep, Gibco, Thermo Fisher Scientific,

Paisley, UK), 4 mM L-Glutamine (L-Glutamine 200 mM, Gibco, Thermo Fisher Scientific, Paisley, UK) and 50 % cellulose solution (Avicel (Sigma Aldrich) 2.5 % w/v in double-distilled $H_2O$) and incubated for 48 hours at 37 °C, 5 % $CO_2$. Overlay was removed and cells were washed three times with PBS. Cells were then fixed with 4 % w/v *p*-formaldehyde in PBS at 4 °C for 30 minutes. Next, cells were stained with crystal violet staining solution (1% w/v crystal violet in 20 % methanol and 80 % distilled water) for 15 minutes. Staining solution was removed and cells were air-dried at room temperature. Virus infected areas were counted and plaque forming units (PFU) per mL calculated.

$$\text{PFU per mL} = \frac{\text{number of plaques } \times \text{reciprocal dilution factor}}{\text{volume inoculum in mL}}$$

**Cytotoxicity Assay:** The influence of the inhibitors at the highest used concentration of 5 µM on the viability of the used A549 cells was determined by using the CellTiter-Glo® assay (Promega GmbH, Mannheim, Germany) according to the instructions of the manufacturer (Figure S4). In brief, A549 cells were seeded in 96-well plates in DMEM supplemented with 10% fetal calf serum, penicillin/streptomycin and L-glutamine at 37 °C, 5 % CO2 until 95 % confluency. Culture medium was exchanged to serum-free infection medium (DMEM, 0.1 % bovine serum albumin, penicillin/streptomycin, L-glutamine) with 5 µM inhibitor or an equivalent concentration of DMSO as control. Cells were then incubated at 37 °C, 5 % CO2 for 72 h until analysis. All treatment conditions were performed as technical triplicates. A slight toxicity was only observed for inhibitor **27.**

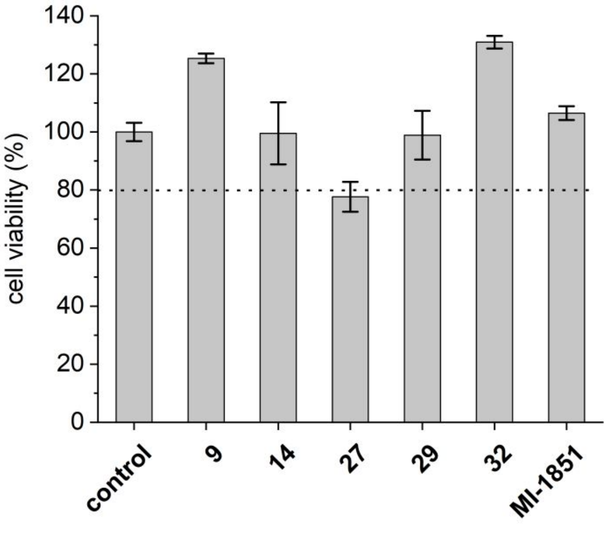


**Figure S4.** Cytotoxicity of the used inhibitors at a concentration of 5 µM after 72 hours incubation with A549 cells. The test was performed by the CellTiter-Glo® assay. The control contains 0.05 % of DMSO, which is identical to the DMSO content at the highest tested inhibitor concentration of 5 µM. The dotted line represents a cell viability of 80 %, a lower cell viability is a sign for toxicity.

## 9. References


(1) Ivanova, T.; Hardes, K.; Kallis, S.; Dahms, S. O.; Than, M. E.; Künzel, S.; Böttcher-Friebertshäuser, E.; Lindberg, I.; Jiao, G. S.; Bartenschlager, R.; Steinmetzer, T. Optimization of Substrate-Analogue Furin Inhibitors. *ChemMedChem* **2017**, *12* (23), 1953–1968. DOI: 10.1002/cmdc.201700596.

(2) Suchý, M.; Elmehriki, A. A. H.; Hudson, R. H. E. A remarkably simple protocol for the N-formylation of amino acid esters and primary amines. *Org. Lett.* **2011**, *13* (15), 3952–3955. DOI: 10.1021/ol201475j. Published Online: Jun. 28, 2011.

(3) Lange, R. W.; Bloch, K.; Heindl, M. R.; Wollenhaupt, J.; Weiss, M. S.; Brandstetter, H.; Klebe, G.; Falcone, F. H.; Böttcher-Friebertshäuser, E.; Dahms, S. O.; Steinmetzer, T. Fragment-Based Design, Synthesis, and Characterization of Aminoisoindole-Derived Furin Inhibitors. *ChemMedChem* **2024**, *19* (9), e202400057. DOI: 10.1002/cmdc.202400057. Published Online: Mar. 11, 2024.
